# Tandem processes promoted by a hydrogen shift in 6-arylfulvenes bearing acetalic units at *ortho* position: a combined experimental and computational study

**DOI:** 10.3762/bjoc.12.28

**Published:** 2016-02-11

**Authors:** Mateo Alajarin, Marta Marin-Luna, Pilar Sanchez-Andrada, Angel Vidal

**Affiliations:** 1 Departamento de Química Orgánica, Universidad de Murcia, Facultad de Química, Regional Campus of International Excellence “Campus Mare Nostrum”, Espinardo, 30100 Murcia (Spain); 2 University Centre of Defence at the Spanish Air Force Academy, Base Aerea de San Javier, C/ Coronel López Peña s/n, 30720, Santiago de la Ribera, Murcia, Spain

**Keywords:** acetal, benzindenes, DFT calculations, fulvene, hydrogen shift

## Abstract

6-Phenylfulvenes bearing (1,3-dioxolan or dioxan)-2-yl substituents at *ortho* position convert into mixtures of 4- and 9-(hydroxy)alkoxy-substituted benz[*f*]indenes as result of cascade processes initiated by a thermally activated hydrogen shift. Structurally related fulvenes with non-cyclic acetalic units afforded mixtures of 4- and 9-alkoxybenz[*f*]indenes under similar thermal conditions. Mechanistic paths promoted by an initial [1,4]-, [1,5]-, [1,7]- or [1,9]-H shift are conceivable for explaining these conversions. Deuterium labelling experiments exclude the [1,4]-hydride shift as the first step. A computational study scrutinized the reaction channels of these tandem conversions starting by [1,5]-, [1,7]- and [1,9]-H shifts, revealing that this first step is the rate-determining one and that the [1,9]-H shift is the one with the lowest energy barrier.

## Introduction

Fulvenes (also known as pentafulvenes), [1–4] a unique class of trienes, have intrigued chemists for decades due to their theoretical interest [5–6] and synthetic applications [7–15]. In this latter sense, fulvenes can be involved in multiple modes of cyclization processes such as [4 + 2] [7–8], [6 + 2] [9–11], and [6 + 3] [12–15] cycloaddition reactions resulting in the construction of diverse fused ring systems. Other classical pericyclic processes that may potentially occur in fulvene fragments (electrocyclic and ene reactions, sigmatropic rearrangements and shifts) have received less attention, most probably with the only exception of the Claisen rearrangement [16]. Notably, thermally promoted H-shifts remain, to the best of our knowledge, completely unexplored in fulvene frameworks [17–18].

**Scheme 1:**
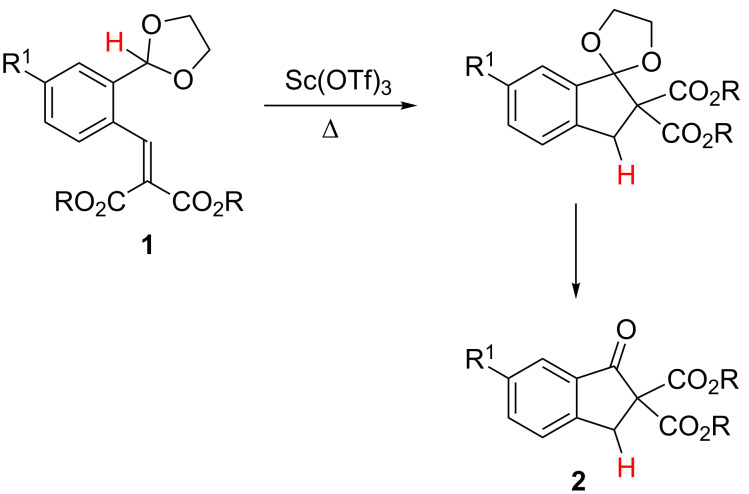
Lewis acid-catalyzed [1,4]-H transfer/1,5-electrocyclization tandem processes of benzylidenemalonates **1** leading to indan-1-ones **2**.

A part of our recent research focused on showing the special ability of cyclic acetalic functions (1,3-dioxolanes, thiolanes, oxathiolanes, dioxanes, dithianes, oxathianes) for promoting the migration of its acetalic H atom in a hydride-like manner. As result, we have disclosed a variety of tandem processes initiated by [1,5]- and [1,4]-hydride shifts from the acetalic carbon atom toward electrophilic molecular fragments [19–27]. Thus, we have reported that *ortho*-(1,3-dioxolan-2-yl)benzylidenemalonates **1** undergo tandem hydride shift/cyclization sequences leading to the corresponding indan-1-one-2,2-dicarboxylates **2**. Remarkably, the first step of these processes consists of an uncommon [1,4]-hydride shift of the acetalic H atom following the activation of the benzylidenemalonate fragment by scandium(III) triflate as the catalyst (Scheme 1) [27].

Because fulvenes are widely used as the direct precursors of cyclopentadienyl anions following the addition of nucleophiles, including the hydride anion, to its exocyclic sp^2^ carbon atom [28–29], we wondered whether acetalic functions could be employed as internal H donors in intramolecular hydride-like shifts, analogous to that highlighted in Scheme 1, toward fulvene frameworks.

**Scheme 2:**
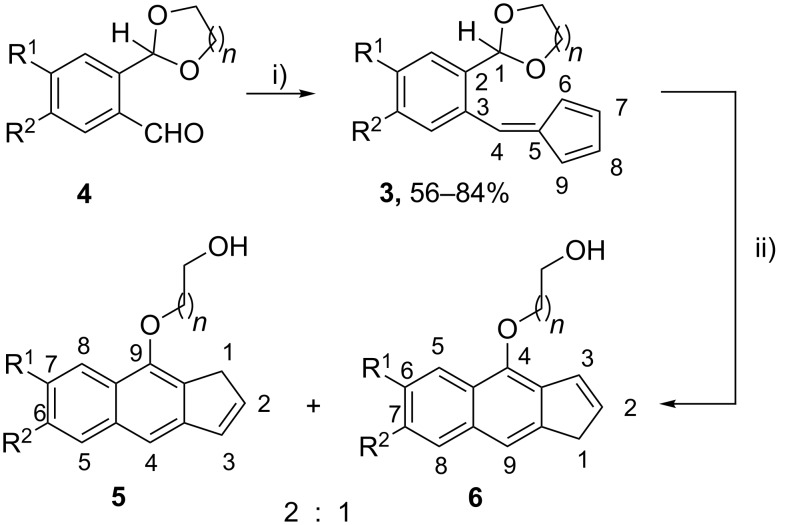
Preparation of benz[*f*]indenes **5** and **6**. Reagents and conditions: i) cyclopentadiene, pyrrolidine, anhydrous methanol, rt, 10 h; ii) DMSO, microwave, 120 °C, 120 W, 20–40 min.

With this goal in mind we designed the unknown acetal-fulvenes **3** (Scheme 2) as potential candidates for assaying the [1,4]-hydride shift of its acetalic H atom toward the exocyclic C4 carbon atom of the fulvene fragment (note the numbering in the Scheme 2). At this point it is worth noting that other possible H migrations were not ruled out at the outset of this investigation. For example, C5 and C7 may be the respective termini of sigmatropic [1,5]-H or [1,7]-H shifts, whereas C6 could be also prone to participate in a less common [1,9]-H shift [30–32]. The variety of potential intramolecular H migrations in these reactive species might well justify by itself the research here disclosed.

## Results and Discussion

### Experimental study

The starting acetal-fulvenes **3** were prepared by the condensation of substituted 2-(1,3-dioxolan-2-yl)benzaldehydes **4** with cyclopentadiene following a well-established synthetic methodology [33]. With the aim of promoting the desired hydride transfer by thermal activation, we first heated the parent acetal-fulvene **3a** under a variety of reaction conditions (benzene 110 °C sealed tube; toluene 120 °C sealed tube; DMF 120 °C) but unfortunately without success. Only when a DMSO solution of **3a** was heated at 120 °C for 7 h the acetal-fulvene converted into a complex mixture from which we were able to isolate the benz[*f*]indenes **5a** and **6a**, in a relative 2:1 ratio and a poor global yield (34%). We next tested the same and similar processes in a microwave apparatus. As presumed, conversions of a series of acetal-fulvenes **3a–f** under 120 W microwave irradiation at 120 °C in DMSO required much shorter reaction times (20–40 min) and led in all cases to the isolation of the respective benz[*f*]indenes **5** and **6**, in a 2:1 ratio (Scheme 2). The overall yield of the isomeric mixtures **5** + **6** did not improve significantly with respect to the conventional thermal conditions previously used with **3a**, ranging from medium to low as depicted in Table 1. Despite our chromatographic (column, thin-layer) efforts, additional pure products other than **5** and **6** could not be isolated from the complex final reaction mixtures.

**Table 1:** Benz[*f*]indenes **5** and **6**.

Compounds	*n*	R^1^	R^2^	Irradiation time (min)	**5** + **6** Yield (%)
					
**5a**, **6a**	1	H	H	25	34
**5b**, **6b**	1	OCH_3_	H	40	30
**5c**, **6c**	1	OCH_2_O		25	45
**5d**, **6d**	2	H	H	25	57
**5e**, **6e**	2	OCH_3_	H	40	46
**5f**, **6f**	2	OCH_2_O		20	53

The structural determination of the reaction products, the 9- and 4-hydroxyalkoxy regioisomers **5** and **6**, was easily accomplished by using the habitual analytical and spectroscopic techniques, whereas the distinction between each two regioisomers is basically supported by ^1^H NMR NOE difference experiments. For the major isomers **5** enhancements of the signals due to the *H*-C2 and C*H*_2_-OAr protons were observed when the methylenic protons were irradiated. In contrast, similar irradiation experiments with the minor isomers **6** revealed enhancements of *H*-C2 and *H*-C9.

**Scheme 3:**
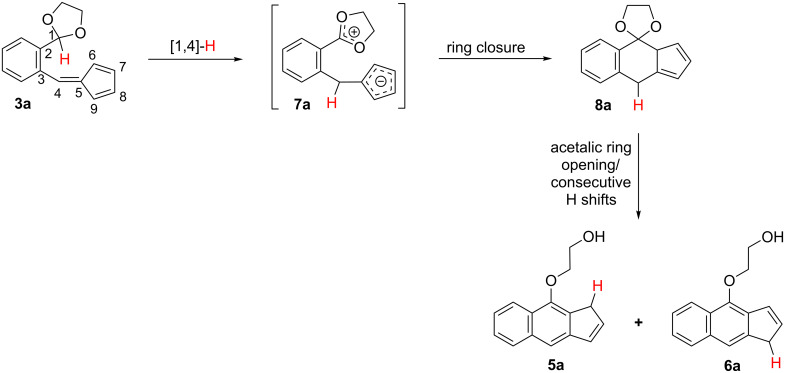
Postulated reaction path for the conversion **3a** → **5a** + **6a** initiated by a [1,4]-hydride shift.

As the H atom at the acetalic carbon of the initial fulvenes **3** apparently appears at the methylene group of both isomeric reaction products, it seems reasonable to hypothesize that the conversions **3 → 5** + **6** are initiated by an H migration from the acetalic carbon toward the fulvene substructure. If this migration is a [1,4]-hydride shift to the exocyclic C4 carbon atom of the fulvene fragment, as initially postulated, this step (exemplified for the simplest fulvene **3a** in Scheme 3) would lead to the dipolar intermediate **7a** which reasonably would undergo further cyclization to **8a** thus building the benz[*f*]indene substructure present in the final reaction products. For the conversion of intermediate **8a** into the final mixture of **5a** and **6a** several mechanistic paths are conceivable, each one involving additional H shifts and a fragmentation step, the opening of the dioxolane ring by a formal β-elimination reaction.

**Scheme 4:**
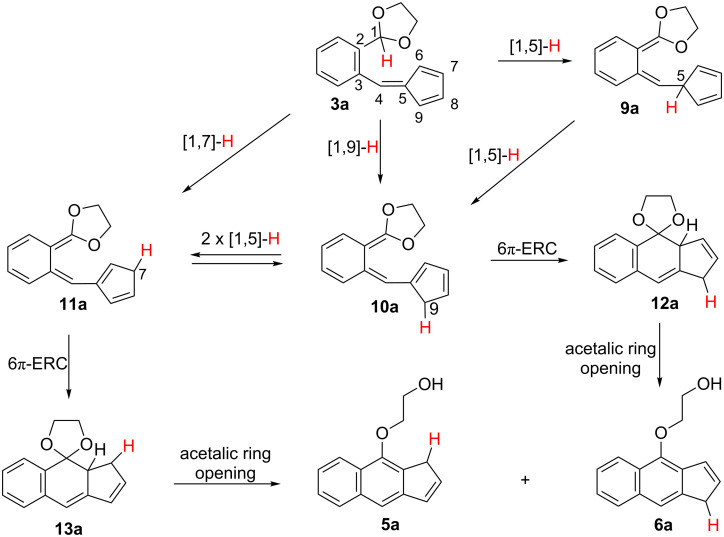
Alternative mechanistic paths for the conversion **3a** → **5a** + **6a** initiated by [1,5]-, [1,7]- or [1,9]-H shifts.

A number of additional mechanistic alternatives arise by considering that the experimentally observed transformations of **3** are initiated by other H shifts alternative to the initially expected [1,4] one. Thus, a [1,5]-H shift from the acetalic carbon of **3a** to the C5 carbon atom of the cyclopentadiene ring, would lead to the transient *ortho*-quinodimethane structure **9a**, which might transform into two similar intermediates, **10a** and **11a**, by a sequence of consecutive [1,5]-H shifts around the cyclopentadiene ring. Next, intermediates **10a** and **11a** would undergo 6π-electrocyclic ring closures (6π-ERC) to the respective dihydrobenzindenes **12a** and **13a**. Finally, these two species would experiment the acetalic ring opening by a formal β-elimination proccess leading to the respective final products **5a** and **6a** (Scheme 4).

This mechanistic scheme is further complicated when considering that *ortho*-quinodimethane intermediates **10a** and **11a** could also result from the respective [1,9]-H and [1,7]-H shifts occurring in the starting acetal-fulvene **3a** (Scheme 4). Thus, the different mechanistic paths represented in Scheme 4 for explaining the conversion **3a** → **5a** + **6a** share several common steps, essentially differing in their first hydrogen shift, [1,5]-, [1,7]- or [1,9]-H.

**Scheme 5:**
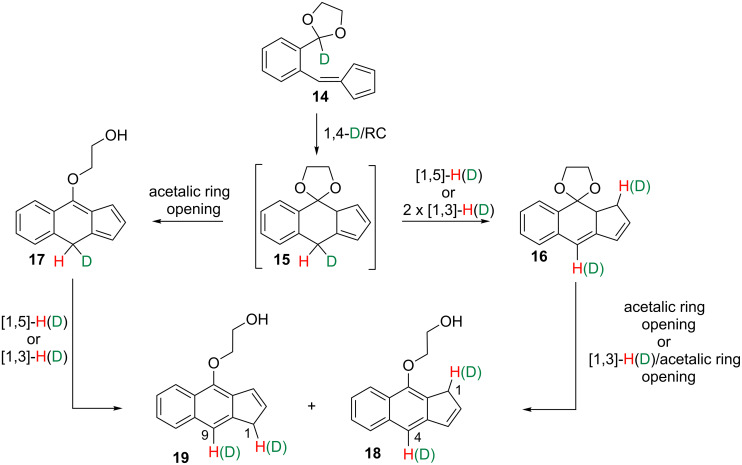
Postulated outcome of the conversion **14 → 18** + **19** initiated by a [1,4]-deuteride shift.

Obviously, any attempt to discern which one is the actual reaction path (if only one!) among the range of potential mechanistic alternatives for these transformations seems a huge task. Seeking for additional experimental data in order to approach such objective, we reasoned that the mechanism initiated by a [1,4]-hydride shift, as summarized in Scheme 3, could be differentiated from those starting by [1,5]-, [1,7]- or [1,9]-H shifts, represented in Scheme 4, by deuterium labelling experiments. Thus, if the conversion of the deuterated acetal-fulvene **14**, in which deuterium replaces the proton at the acetalic carbon of **3a**, was actually initiated by a [1,4]-deuteride shift, the transformation of the dihydrobenzo[*f*]indenic species **15**, which should form in first instance, would yield the final benz[*f*]indenes via intermediates **16** and **17**. These are labelled with deuterium at C4 of the major product **18** and at C9 of the minor one **19** as well as probably at additional positions of the fused five-membered ring (Scheme 5).

**Scheme 6:**
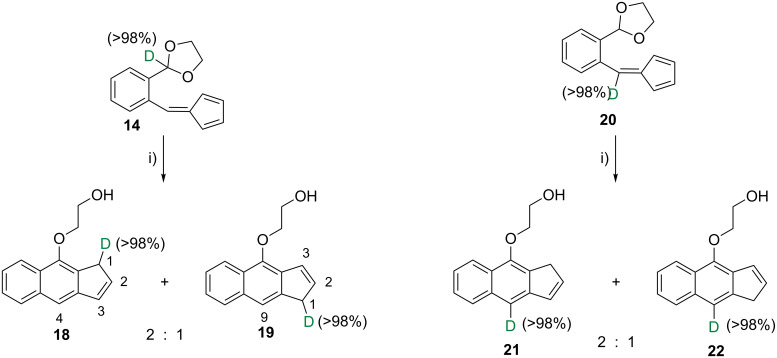
Preparation of deuterated benz[*f*]indenes **18** + **19** and **21** + **22**. Reagents and conditions*:* i) DMSO, microwave, 120 °C, 120 W, 40 min.

With this in mind we prepared the monodeuterated acetal-fulvene **14** (see Supporting Information File 1) and submitted it to the habitual reaction conditions. As result, a mixture of the monodeuterated benz[*f*]indenes **18** and **19** was obtained, again in a relative 2:1 ratio (Scheme 6). ^1^H NMR analyses showed that only protons, not deuteriums, were linked to the C4 atom of **18** and to C9 of **19**. Instead, one deuterium atom is found at the methylene group of each regioisomer.

Moreover, we carried out a second labelling experiment by using now as starting material the monodeuterated acetal-fulvene **20** bearing the deuterium atom at C4. This species converted into a 2:1 mixture of the monodeuterated regioisomers **21** and **22** as the only isolated reaction products (Scheme 6). In both compounds, the deuteration percentage at their respective C4 and C9 positions was determined, by ^1^H NMR analyses, to be higher than 98%.

This latter result shows that the deuterium atom attached at C4 in the original acetal-fulvene does not migrate in the course of the reaction. In combination, these two labelling experiments are conclusive for discarding an initial [1,4]-deuteride (or hydride) shift as the first step of the previously discussed conversions since, in such a case, we should have found deuterium linked to C4/C9 of the benzindenes **18**/**19** resulting from the first labelling experiment.

**Scheme 7:**
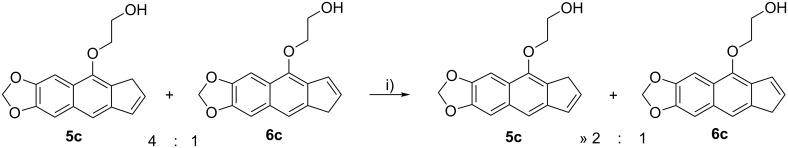
Reagents and conditions*:* i) triethylamine (10%), DMSO, rt, 2 h.

Next we tried to understand why the regioisomeric benz[*f*]indenes were, in all our reactions, produced in a ratio close to 2:1. It is well known that 1*H*-indenes are prone to undergo isomerization by H or group migrations at its cyclopentadiene ring [34–47]. Consequently, we postulated that such an isomeric ratio would correspond to the thermodynamic equilibrium between both isomers, **5** and **6**, established by two consecutive [1,5]-H shifts around its five-membered ring. To test this hypothesis, we heated a 4:1 mixture of isomeric **5c** and **6c** in deuterated DMSO solution at 120 °C for 24 h. In this way we could verify by ^1^H NMR analyses of reaction aliquots that the initial isomeric ratio remained constant over time and heating. Interestingly, the initial 4:1 ratio changed to 2:1 at the end of a related experiment carried out by stirring a DMSO solution of the same isomeric mixture in the presence of a catalytic amount of triethylamine at room temperature for 2 h (Scheme 7).

This result seems to indicate that the experimentally recurrent 2:1 proportion between the regioisomeric products **5** and **6**, reached by equilibration in the latter experiment, should be due to the presence of adventitious minor amounts of basic species either in the DMSO solutions of the experiments or in the course of the processing of the crude reaction mixtures and the purification steps.

**Scheme 8:**
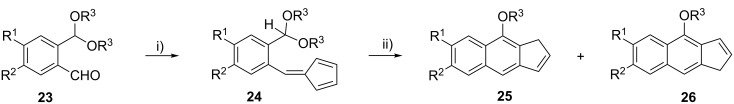
Preparation of benz[*f*]indenes **25** and **26**. Reagents and conditions: i) cyclopentadiene, pyrrolidine, anhydrous methanol, rt, 10 h; ii) DMSO, microwave, 120 °C, 120 W, 20–40 min.

Besides, we also explored the thermally induced transformations of related fulvenes bearing non-cyclic acetalic units (Scheme 8 and Table 2). To this end, benzaldehydes **23** were transformed into fulvenes **24** by the usual procedure, whereas its microwave heating (DMSO, 120 °C, 120 W) yielded a mixture of the benz[*f*]indenes **25** and **26** in the habitual 2:1 ratio. These conversions most probably occur, in mechanistic terms, similarly to those of the acetal-fulvenes **3** (Scheme 4), although in the present cases with the formal β-elimination of a methanol or ethanol molecule.

**Table 2:** Acetal-fulvenes **24** and benz[*f*]indenes **25** and **26**.

Compounds	R^1^	R^2^	R^3^	**24** Yield (%)	Irradiation time (min)	**25** + **26** Yield (%)
						
**24a**–**26a**	H	H	CH_3_	68	10	54
**24b**–**26b**	OCH_3_	H	CH_3_	71	15	42
**24c**–**26c**	OCH_2_O		CH_3_	81	15	47
**24d**–**26d**	H	H	CH_3_CH_2_	74	15	60

These results show that non-cyclic acetalic units are as effective as the cyclic ones on achieving the conversion of acetal-fulvenes into the corresponding benz[*f*]indenes under microwave irradiation.

### Computational study

**Scheme 9:**
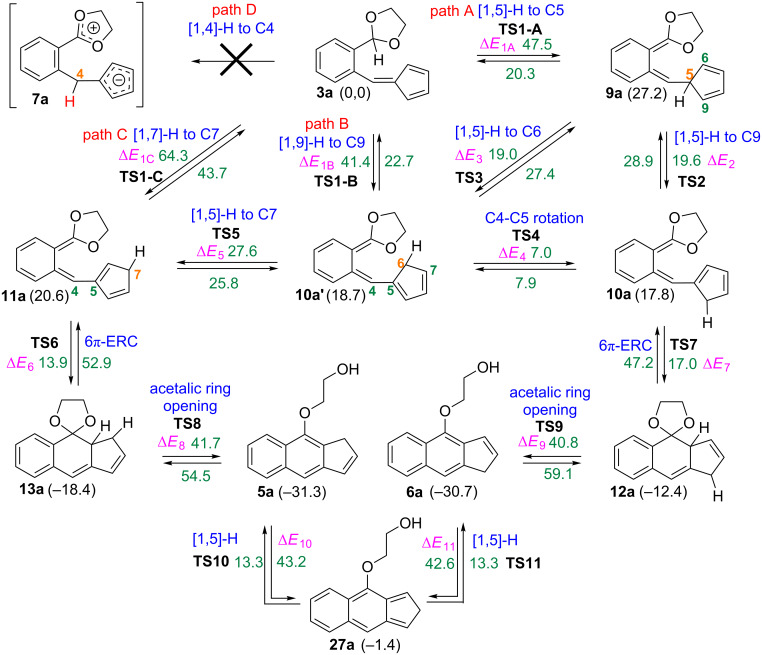
Mechanistic paths for the conversion of fulvene **3a** into the benz[*f*]indenes **5a** and **6a** showing the energy barriers of each step in kcal·mol^−1^ as computed at the B3LYP/6-31+G** theoretical level (between parentheses the relative electronic energies of the minima in kcal·mol^−1^).

**Figure 1:**
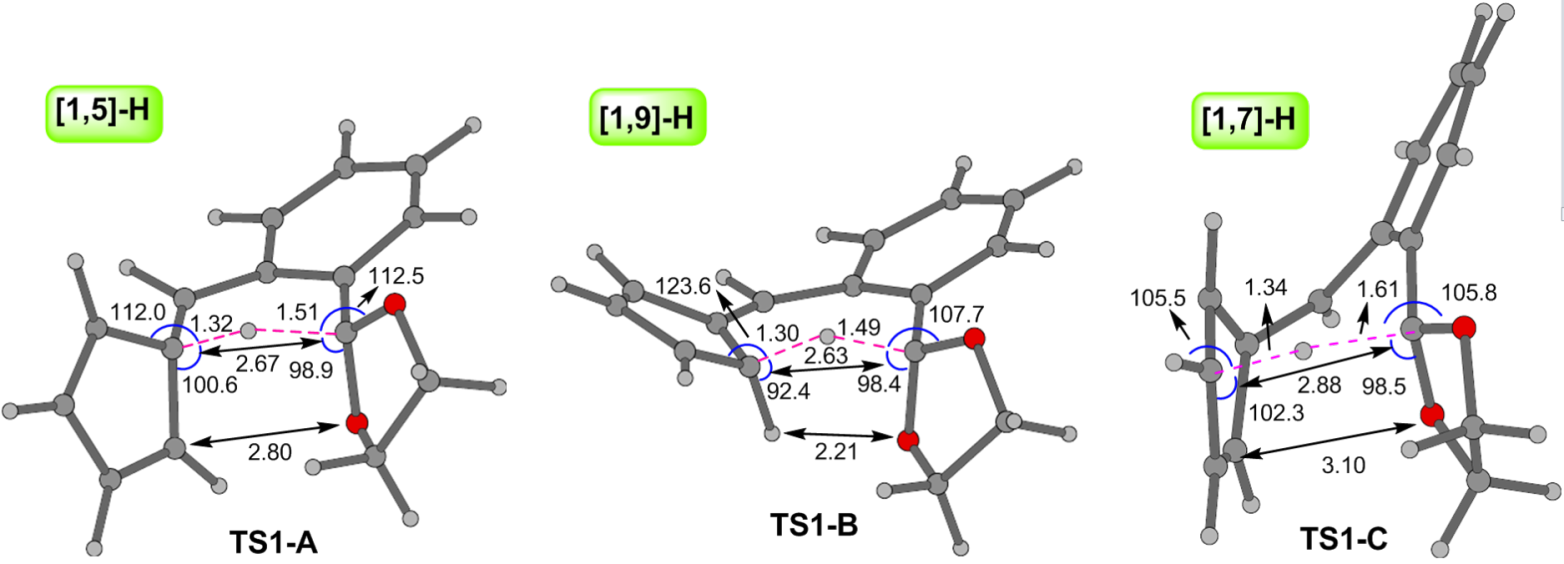
Optimized geometry of transition structures **TS1-A**, **TS1-B**, and **TS1-C** computed at the B3LYP/6-31+G** theoretical level. Distances in Ångstroms, bond angles in degrees.

With the aim of scrutinizing the putative reaction paths leading from the fulvene **3a** to the isomeric benz[*f*]indenes **5a** and **6a** we have carried out a computational study at the B3LYP/6-31+G** theoretical level. Scheme 9 shows the diversity of the computed reaction paths leading from reactants to products. The geometries of the located transition structures associated to the first mechanistic step of each path, the H shift, are shown in Figure 1 [48].

We anticipated three general reaction channels, paths A–C (see Scheme 9). In fact, these pathways only differ in the first step. Path A starts by a [1,5]-H shift, path B by a [1,9]-H shift and path C by a [1,7]-H shift. This overall mechanistic scheme is somewhat complicated due to the number of steps of each reaction path and by the fact that some stationary points belong to more than one of these three pathways. Obviously we also envisaged a fourth mechanistic alternative, path D, just that initially conceived starting by the [1,4]-hydride shift of the acetalic H atom to the exocyclic C4 carbon atom of the fulvene unit. All our efforts aimed to locate its corresponding transition structure were unsuccessful. Nevertheless, this latter mechanistic alternative was discarded by the isotopic labelling experiments commented above.

In the following paragraphs we intend to discuss in a simplified way the results of our calculations on the potential surface of the transformations summarized in Scheme 9.

The first step of path A consists of a [1,5]-H shift from the acetalic carbon atom to C5 (see the numbering in Scheme 9). We located the transition structure **TS1-A**, connecting the fulvene **3a** with the *ortho*-quinodimethane intermediate **9a**. As expected **TS1-A** shows the typical geometry of a suprafacial hydrogen shift (see Figure 1), the computed energy barrier associated to this step being fairly high, 47.5 kcal·mol^−1^. Intermediate **9a** could then experiment two alternative [1,5]-H shifts of its H-C5 proton, migrating either to C6 or to C9, its two vicinal carbon atoms at the cyclopentadiene ring. For the [1,5]-H shift to C9 we located the transition structure **TS2**, 19.6 kcal·mol^−1^ above in energy than **9a**, connecting it with its isomer **10a**. For the alternative [1,5]-H shift to C6 we located the transition structure **TS3**, 19.0 kcal·mol^−1^ above in energy than **9a**, leading to the isomeric structure **10a’** which is in fact a rotamer of **10a**. These two energy barriers are reasonably low and should be easily surmountable under the experimental reaction conditions. Additionally, we were able to locate a transition structure, **TS4**, connecting **10a** and **10a’** by rotation around the C4–C5 single bond. The computed barrier for the conversion of **10** into **10a’** via **TS4** is only 7.0 kcal·mol^−1^, whereas the one for the reverse transformation is 7.9 kcal·mol^−1^. Accordingly, equilibration between **10a** and **10a’** is predicted to occur rapidly by C4–C5 bond rotation rather than by two consecutive [1,5]-H shifts via the isomeric intermediate **9a** (see Scheme 9).

Intermediate **10a’** can also convert into a third *ortho*-quinodimethane isomer **11a** via the transition structure **TS5** by another [1,5]-H shift from C6 to its vicinal C7 carbon atom at the cyclopentadiene ring. The computed energy barrier for this step is 27.6 kcal·mol^−1^, significantly higher than those corresponding to the similar [1,5]-H shifts via **TS2** and **TS3** commented above (19.6 and 19.0 kcal·mol^−1^). The lower barriers of these two latter transition structures are attributable to its more extended conjugation in comparison with the partially cross-conjugated **TS5**.

A series of reaction steps starting from intermediates **10a** and **11a** can lead respectively to the final benzindenes **5a** and **6a**. Thus, intermediate **11a** undergoes a disrotatory 6π-electrocyclic ring closure [49] via the transition structure **TS6** to give the tricyclic species **13a**. By an analogous electrocyclic ring closure through **TS7**, compound **10a** is converted into the isomeric spirotricycle **12a**. The computed energy barriers for these processes are relatively small, 13.9 and 17.0 kcal·mol^−1^ respectively. Again the differences in the extent of the electronic conjugation in these electrocyclization transition states can give account of the relative stabilities of **TS6** and **TS7**.

Two transition structures, **TS8** and **TS9** were located for the respective transformations of **13a** and **12a** into the final benzindenes **5a** and **6a**, involving each one the opening of the acetalic ring with simultaneous transfer of an hydrogen to one of the oxygen atoms (in other words, a concerted β-elimination along a C–C single bond), with the concomitant aromatization of the central ring. The computed energy barriers for these concerted β-eliminations are high, 41.7 and 40.8 kcal·mol^−1^ respectively [50].

Concerning the alternative reaction paths B and C, we have located essentially the same stationary points that in path A with the sole difference of the respective first mechanistic steps. Path B starts with a [1,9]-H sigmatropic rearrangement through **TS1-B** leading to intermediate **10a’**, which then transforms via the mechanistic paths commented above. The geometry of **TS1-B** (see Figure 1) is in accordance with a suprafacial transfer of the H atom between the acetalic carbon and C6. The calculated energy barrier associated to this step is 41.4 kcal·mol^−1^, 6.1 kcal·mol^−1^ lower in energy than the initial [1,5]-H shift of path A. This difference could be rationalized attending to the geometries of both transition structures **TS1-A** and **TS1-B**, more specifically to the distance between the two carbon atom termini of the H migration, shorter in **TS1-B** (2.63 Å) than in **TS1-A** (2.67 Å). Therefore, **TS1-B** is earlier than **TS1-A**. The geometry of **TS1-B** also accounts for its greater conjugation as the spatial positioning of the cyclopentadiene ring allows its orbital overlapping with the rest of the π system. Moreover, **TS1-B** is less sterically congested and also less distorted than **TS1-A** (see the bond distances and bond angles displayed in Figure 1).

For the first step of path C we have located a transition structure, **TS1-C**, connecting fulvene **3a** with the intermediate **11a** by a [1,7]-H sigmatropic shift (see Scheme 9). The computed energy barrier is very high, 64.3 kcal·mol^−1^. This large value is probably due to the heptatrienic fragment not being able of adopting the helical all *s*-*cis* conformation, optimal for an antarafacial [1,7]-H shift, as result of the conformational restrictions imposed by the cyclopentadiene ring (Figure 1). As a consequence, the distance between the two carbon atom termini of the H migration is considerably long (2.88 Å), thus accounting for the high computed energy barrier.

To summarize so far, by comparing the energy barriers associated to the three alternative H shifts, this study predicts that path B is the one involving the lowest energy barrier and, in accordance, the calculations predict that the transformation of fulvene **3a** into the benzindenes **5a** and **6a** should take place via an initial [1,9]-H shift.

Moreover, we also considered that **5a** and **6a** could equilibrate by two consecutive [1,5]-H shifts occurring at the five-membered ring. By exploring the potential energy surface associated to these transformations we were able to locate transition structures **TS10** and **TS11** connecting **5a** and **6a** through the intermediate **27a** (see Scheme 9). The computed energy barriers for the conversions **5a** → **27a**, and **6a** → **27a** are fairly high, 43.2 and 42.6 kcal·mol^−1^, respectively, as expected on going from a fully aromatic central ring to an *ortho*-quinoid structure, whereas those calculated for the reverse conversions are considerably lower (13.3 kcal·mol^−1^ in both cases).

By analysing the overall picture showing the different mechanistic paths connecting **3a** with the final benzindenes **5a** and **6a** we can extract the following conclusions:

1) On going from **3a** to the two tricyclic intermediates **12a** and **13a**, the rate determining reaction step is predicted to be the first one, i.e. the initial hydrogen migration, and this study predicts that a [1,9]-H shift is less costly in terms of energy than a [1,5]-H or [1,7]-H one. The more extended conjugation, the lower steric hindrance and the shorter C–C distance between the two carbon atoms termini of the H migration in **TS1-B**, the transition structure of the [1,9]-H shift, can account for its lower energy when compared with those of the alternative two other H shifts. Nevertheless, the higher electronic conjugation in **TS1-B**, in comparison with those of **TS1-A** and **TS1-C**, could be also decisive in accounting for the differences in the respective energy barriers.

2) Concerning the two key polyenes **10a** and **11a**, precursors of the tricycles **12a** and **13a**, respectively, the computed energy barriers of this study show that i) **10a** most probably forms by an easy rotational isomerization of **10a’**, instead of the alternative path involving the [1,5]-H shift from **9a**; and ii) **11a** will form mainly from **10a’** rather than directly from **3a**. That is, **10a** and **11a** should form via intermediate **10a’** resulting from the [1,9]-H shift**,** which then transforms into **11a** by a [1,5]-H shift (barrier of 27.6 kcal·mol^−1^) or equilibrates to its rotamer **10a** (barrier of 7.0 kcal·mol^−1^).

3) The two 6π-electrocyclic ring closures converting respectively **10a** and **11a** into **12a** and **13a** involve low energy barriers, that corresponding to the conversion of **11a** into **13a** being lower than that of **10a** into **12a** (13.9 and 17.0 kcal·mol^−1^, respectively).

4) The overall processes **3a** → **5a** and **3a** → **6a** are exothermic by 31.3 and 30.7 kcal·mol^−1^, respectively. The interconversion between **5a** and **6a** is predicted to take place via two consecutive [1,5]-H shifts at the pentagonal ring through transient intermediate **27a**.

As a final point, we have also explored the potential energy surface associated to these conversions by considering the effect of the solvent used in the experimental study, DMSO. The computed energy barriers in the gas phase and in DMSO are depicted in Table 3.

**Table 3:** Electronic energy barriers in kcal·mol^–1^ for the conversions of acetal fulvene **3a** into indenes **5a** and **6a** calculated at the B3LYP/6-31+G** theoretical level.^a^

**3a** → **5a** + **6a**	Gas	DMSO
		
Δ*E*_1A_	47.5	47.3
Δ*E*_2_	19.6	19.0
Δ*E*_3_	19.0	17.6
Δ*E*_1B_	41.4	39.9
Δ*E*_4_	7.0	6.9
Δ*E*_5_	27.6	26.6
Δ*E*_1C_	64.3	63.6
Δ*E*_6_	13.9	14.4
Δ*E*_7_	17.0	17.5
Δ*E*_8_	41.7	36.8
Δ*E*_9_	40.8	37.3
Δ*E*_10_	43.2	42.3
Δ*E*_11_	42.6	41.4

^a^See Scheme 9 for the notation of the energy barriers.

In general, the values of the energy barriers do not vary noticeably in DMSO when compared with those in gas phase. Only Δ*E*_8_ and Δ*E*_9_ are appreciably lower in DMSO with respect to those in gas phase by 3.9 and 3.5 kcal·mol^−1^, respectively (Table 3). Consequently, according to these calculations, the rate determining step in the transformations **3a** → **5a** and **3a** → **6a** in DMSO should be the first one, i.e. the [1,9]-H shift, with an energy barrier slightly lower than that calculated in the gas phase.

In summary, this computational study shows that the conversion of fulvene **3a** into the benzindenes **5a** and **6a** could take place by a variety of alternative reaction paths according to a complicated mechanistic scheme. By analysing in detail the energy barriers computed for each mechanistic step, the energetically preferred path starts with a [1,9]-H sigmatropic rearrangement of the acetalic hydrogen atom leading to an *ortho*-quinodimethane intermediate, further transforming into the isomeric final products by two alternative reaction channels. These two latter pathways may involve up to three consecutive steps such as [1,5]-H shifts, 6π-electrocyclic ring closures, C–C rotations and formal β-eliminations. The interconversion between the isomeric benzindenes **5a** and **6a** could also occur by means of two consecutive [1,5]-H shifts through an unstable benzisoindene intermediate.

## Conclusion

The ability of benzofulvenes bearing 1,3-dioxolane or -dioxane units in *ortho* position for undergoing cascade processes initiated by an H shift step has been tested. Such acetal-fulvenes, under thermal activation, transformed into mixtures of the corresponding 4- and 9-(hydroxy)alkoxy-substituted benz[*f*]indenes in a 1:2 ratio. Analogous fulvenes bearing non-cyclic dialkoxymethyl units when submitted to similar thermal conditions also afforded 1:2 mixtures of the respective 4 and 9-alkoxybenz[*f*]indenes. Such 1:2 ratio has been interpreted as the one corresponding to the thermodynamic equilibrium established between both isomers. Mechanistic paths initiated by an initial [1,4]-, [1,5]-, [1,7]- or [1,9]-H shift are conceivable for explaining these cascade transformations leading to benz[*f*]indenes. The results of deuterium labelling experiments excluded a [1,4]-hydride shift as the initial step. The reaction of the unsubstituted 1,3-dioxolane-fulvene has been computationally studied by DFT methods. The results of this study revealed that the first mechanistic step, the H shift, is the rate-determining one and that, among the alternative [1,5]-, [1,7]- or [1,9]-H migrations, the energy barrier of the [1,9]-H shift is the lowest one, a fact that is rationalised attending to some key structural and electronic characteristics of the respective transition states. The calculations have also shown that the tandem conversions of the starting fulvenes into benz[*f*]indenes are exergonics, the 9-substituted regioisomer being the thermodynamically-controlled major product, in accordance with the experimental results.

## Supporting Information

File 1 Experimental part.

File 2 Computational part.
