# Supplementary material for: Tandem processes promoted by a hydrogen shift in 6-arylfulvenes bearing acetalic units at ortho position: a combined experimental and computational study
Source: Beilstein J Org Chem. 2016 Feb 11;12:260–70. doi: 10.3762/bjoc.12.28 (PMC4778534; doi:10.3762/bjoc.12.28)

**Supporting Information**

**for**

# Tandem processes promoted by a hydrogen shift in 6-arylfulvenes bearing acetalic units at *ortho* position: a combined experimental and computational study

Mateo Alajarin[1], Marta Marin-Luna[1], Pilar Sanchez-Andrada*[2] and Angel Vidal[1]

Address: [1]Departamento de Química Orgánica, Universidad de Murcia, Facultad de Química, Regional Campus of International Excellence "Campus Mare Nostrum", Espinardo, 30100 Murcia (Spain) and [2]University Centre of Defence at the Spanish Air Force Academy, Base Aerea de San Javier, C/ Coronel López Peña s/n, 30720, Santiago de la Ribera, Murcia, Spain

Email: Pilar Sanchez-Andrada - pilar.sanchez@cud.upct.es

*Corresponding author

## Experimental part

### General Methods

All melting points are uncorrected. Infrared (IR) spectra were recorded as Nujol emulsions or neats. $^{1}$H NMR spectra were recorded in $CDCl_3$, $CD_2Cl_2$ or DMSO-$d_6$ at 300 or 400 MHz. $^{13}$C NMR spectra were recorded in $CDCl_3$, $CD_2Cl_2$ or DMSO-$d_6$ at 75 or 100 MHz. The chemical shifts are expressed in ppm, relative to $Me_4Si$ at $\delta$ = 0.00 ppm for $^{1}$H, while the chemical shifts for $^{13}$C are reported relative to the resonance of $CDCl_3$ $\delta$ = 77.1 ppm, $CD_2Cl_2$ $\delta$ = 54.0 ppm or DMSO-$d_6$ = 39.5 ppm. Mass spectra were recorded on a HPLC/MS TOF 6220 Agilent Technologies apparatus.

### Materials

2-(1,3-Dioxolan-2-yl)benzaldehyde (**4a**) [1], 2-(1,3-dioxolan-2-yl)-4-methoxybenzaldehyde (**4b**) [2]**,** 6-(1,3-dioxolan-2-yl)-1,3-benzodioxole-5-carboxaldehyde (**4c**) [3], 2-(1,3-dioxan-2-yl)benzaldehyde (**4d**) [4], 6-(1,3-dioxan-2-yl)-1,3-benzodioxole-

5-carboxaldehyde (**4f**) [4], 2-(dimethoxymethyl)benzaldehyde (**23a**) [5], 2-(dimethoxymethyl)-4-methoxybenzaldehyde (**23b**) [6], 6-(dimethoxymethyl)-1,3-benzodioxole-5-carboxaldehyde (**23c**) [7], 2-(diethoxymethyl)benzaldehyde (**23d**) [8] and 2-(1,3-dioxolan-2-yl-2*d*)benzaldehyde [9] were prepared following published experimental procedures.

**Preparation of 2-(1,3-dioxan-2-yl)-4-methoxybenzaldehyde (4e)**

*n*-BuLi [5.8 mL, 2.6 M in hexane, 14.4 mmol] was added dropwise to a solution of 2-(2-bromo-5-methoxyphenyl)-1,3-dioxane (3.4 g, 12.0 mmol) in anhydrous tetrahydrofuran (50 mL), at −78 °C under an atmosphere of nitrogen. The solution was stirred at −78 °C for 30 min. Then, a solution of *N*,*N*-dimethylformamide (1.12 mL, 14.4 mmol) in tetrahydrofuran (10 mL) was added dropwise. The reaction mixture was stirred at −78 °C for 15 min, warmed to room temperature and the stirring was continued for 3 h. After quenching the reaction by the addition of water (25 mL), the mixture was extracted with ethyl acetate (2 × 30 mL). The combined organic layers were washed with water (2 × 100 mL) and dried over anhydrous $MgSO_4$. The solvent was removed under reduced pressure and the resulting oil was purified by silica gel column chromatography, using hexanes/diethyl ether (3:2, v/v) as eluent.

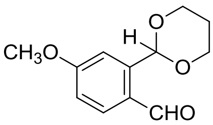


**2-(1,3-Dioxan-2-yl)-4-methoxybenzaldehyde 4e:** (65%); mp 105-107 ºC (colourless prims, diethyl ether); $\nu_{max}$ (Neat)/cm$^{-1}$ 1684 (vs), 1601 (s), 1089 (s); $\delta_H$ (400 MHz, $CDCl_3$)1.44-1.49 (1H, m), 2.19-2.28 (1H, m), 3.87 (3H, m), 4.00-4.07 (2H, m), 4.24-4.28 (2H, m), 6.08 (1H, m), 6.94 (1H, dd, *J* = 2.8, 8.4Hz), 7.23 (1H, d, *J* = 2.8 Hz), 7.85 (1H, d, *J* = 8.4 Hz), 10.3 (1H, s); $\delta_C$ (100 MHz, $CDCl_3$) 25.7, 55.6, 67.2, 99.1, 112.1, 114.6, 126.8 (s), 133.0, 142.1 (s), 163.9 (s), 190.8 (s); HRMS (ESI) *m/z* calcd for $C_{12}H_{15}O_4$ $[M+H]^+$ 223.0965; found: 223.0963.

**Procedure for the preparation of fulvenes 3 and 24**

To a solution of the appropriate benzaldehyde **4** or **23** (3 mmol) in dry methanol (15 mL) were added cyclopentadiene (0.4 g, 6 mmol) and pyrrolidine (0.43 g, 6 mmol). The reaction mixture was stirred at room temperature overnight. Then, the reaction mixture was acidified with acid acetic (1 mL), diluted with water (40 mL) and extracted with diethyl ether (3 × 20 mL). The organic layer was washed with brine (2 × 10 mL), dried over

anhydrous $MgSO_4$, filtered and evaporated under reduced pressure. The resulting residue was purified by silica gel column chromatography.

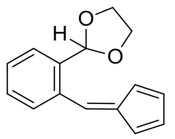

**Fulvene 3a:** eluent for column chromatography: hexanes/diethyl ether (9:1 v/v); (65%); red oil; $\nu_{max}$ (Neat)/cm$^{-1}$ 1626 (vs), 1600 (s), 1572 (s); $\delta_H$ (300 MHz, DMSO-d$_6$, 100 ºC) 3.95-4.09 (4H, m), 5.99 (1H, s), 6.37-6.43 (2H, m), 6.50-6.53 (1H, m), 6.61-6.64 (1H, m), 7.39-7.49 (3H, m), 7.58-7.61 (1H, m), 7.67 (1H, s); $\delta_C$ (75 MHz, DMSO-d$_6$, 100 ºC) 64.2, 100.8, 119.9, 125.9, 126.0, 127.8, 128.0, 130.2, 130.7, 134.2, 134.6 (s), 135.0, 136.4 (s), 145.3 (s); HRMS (ESI) *m/z* calcd for $C_{15}H_{15}O_2$ [M+H]$^+$ 227.1067; found: 227.1067.

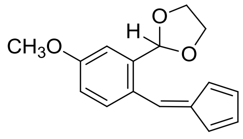

**Fulvene 3b:** eluent for column chromatography: hexanes/diethyl ether (9:1 v/v); (75%); red oil; $\nu_{max}$ (Neat)/cm$^{-1}$ 1601 (vs), 1496 (s), 1287 (s); $\delta_H$ (400 MHz, $CDCl_3$) 3.87 (3H, s), 4.04-4.17 (4H, m), 6.02 (1H, s), 6.36-6.37 (1H, m ), 6.53-6.55 (2H, m), 6.64-6.65 (1H, m), 6.95 (1H, dd, *J* = 2.8, 8.4 Hz), 7.21 (1H, d, *J* = 2.8 Hz), 7.53 (1H, d, *J* = 8.4 Hz), 7.60 (1H, s); $\delta_C$ (100 MHz, $CDCl_3$) 55.4, 65.4, 101.5, 111.6, 114.6, 120.9, 126.5, 128.1 (s), 130.8, 133.9, 134.7, 135.6, 138.4 (s), 145.2 (s), 160.3 (s); HRMS (ESI) *m/z* calcd for $C_{16}H_{17}O_3$ [M+H]$^+$ 257.1172; found: 257.1182.

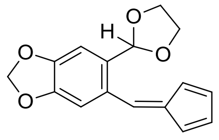

**Fulvene 3c:** eluent for column chromatography: hexanes/diethyl ether (9:1 v/v); (65%); red oil; $\nu_{max}$ (Neat)/cm$^{-1}$ 1602 (m), 1502 (vs), 1484 (vs); $\delta_H$ (400 MHz, $CDCl_3$) 4.01-4.16 (4H, m), 5.97 (2H, s), 6.01 (1H, s), 6.32-6.35 (1H, m), 6.52-6.53 (2H, m), 6.63-6.65 (1H, m), 7.04 (1H, s), 7.14 (1H, s), 7.52 (1H, s); $\delta_C$ (100 MHz, $CDCl_3$) 65.4, 101.1, 101.6, 106.7, 111.8, 120.6, 126.6, 129.9 (s), 131.1, 131.6 (s), 135.1, 145.7 (s), 148.1 (s), 148.3 (s); HRMS (ESI) *m/z* calcd for $C_{16}H_{15}O_4$ [M+H]$^+$ 271.0965; found: 271.0976.

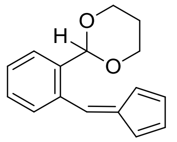

**Fulvene 3d:** eluent for column chromatography: hexanes/diethyl ether (9:1 v/v); (56%); red oil; $\nu_{max}$ (Neat)/cm$^{-1}$ 1602 (w), 1466 (m), 1376 (m); $\delta_H$ (400 MHz, $CDCl_3$) 1.44-1.47 (1H, m), 2.20-2.32 (1H, m), 3.98 (2H, td, *J* = 2.4, 12.0 Hz), 4.28 (2H, dd, *J* = 4.0, 10.8 Hz), 5.67 (1H, s), 6.38 (1H, dt, *J* = 1.7, 5.2 Hz), 6.47-6.49 (1H, m), 6.56-6.58 (1H, m), 6.62-6.65 (1H, m), 7.38-7.40 (2H, m), 7.46-7.48 (1H, m), 7.63 (1H, s), 7.67-7.69 (1H, m); $\delta_C$ (100 MHz, $CDCl_3$) 25.8, 67.6, 100.2, 121.4,

126.3, 126.4, 128.7, 128.9, 131.6, 132.0, 134.8, 134.9 (s), 136.2, 137.4 (s), 146.5 (s); HRMS (ESI) *m/z* calcd for $C_{16}H_{17}O_2$ $[M+H]^+$ 241.1223; found: 241.1217.

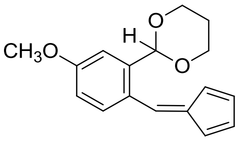


**Fulvene 3e:** eluent for column chromatography: hexanes/diethyl ether (9:1 v/v); (84%); red oil; $\nu_{max}$ (Neat)/$cm^{-1}$ 1602 (vs), 1498 (s), 1466 (m); $\delta_H$ (300 MHz, $CDCl_3$) 1.44-1.49 (1H, m), 2.20-2.33 (1H, m), 3.87 (3H, s), 3.99 (2H, td, *J* = 1.8, 12.0 Hz), 4.28 (2H, dd, *J* = 4.8, 10.8 Hz), 5.67 (1H, s), 6.38 (1H, dt, *J* = 1.5, 5.1 Hz), 6.52-6.56 (2H, m), 6.62-6.66 (1H, m), 6.93 (1H, dd, *J* = 2.7, 8.4 Hz), 7.25 (1H, d, *J* = 2.7 Hz), 7.48 (1H, d, *J* = 8.4 Hz), 7.55 (1H, s); $\delta_C$ (75 MHz, $CDCl_3$) 25.7, 55.5, 67.6, 99.7, 111.3, 114.9, 121.1, 126.5, 127.3 (s), 130.8, 133.7, 134.5, 136.0, 139.1 (s), 145.1 (s), 160.4 (s); HRMS (ESI) *m/z* calcd for $C_{17}H_{19}O_3$ $[M+H]^+$ 271.1329; found: 271.1339.

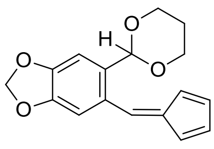


**Fulvene 3f:** eluent for column chromatography: hexanes/diethyl ether (9:1 v/v); (74%); red oil; $\nu_{max}$ (Neat)/$cm^{-1}$ 1602 (vs), 1503 (vs), 1486 (vs); $\delta_H$ (300 MHz, $CDCl_3$) 1.40-1.45 (1H, m), 2.14-2.30 (1H, m), 3.96 (2H, td, *J* = 1.8, 12.3 Hz), 4.25 (2H, dd, *J* = 4.8, 10.8 Hz), 5.63 (1H, s), 5.98 (2H, s), 6.37-6.39 (1H, m), 6.55-6.57 (2H, m), 6.66-6.68 (1H, m), 7.05 (1H, s), 7.23 (1H, s), 7.51 (1H, s); $\delta_C$ (75 MHz, $CDCl_3$) 25.5, 67.3, 99.2, 101.4, 106.9, 111.3, 120.6, 126.4, 128.6 (s), 130.9, 132.7 (s), 134.8, 135.4, 145.4 (s), 147.6 (s), 148.2 (s); HRMS (ESI) *m/z* calcd for $C_{17}H_{17}O_4$ $[M+H]^+$ 285.1121; found: 285.1131.

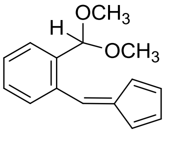


**Fulvene 24a:** eluent for column chromatography: hexanes/diethyl ether (9:1 v/v); (68%); red oil; $\nu_{max}$ (Neat)/$cm^{-1}$ 1626 (vs), 1599 (vs), 1484 (vs); $\delta_H$ (400 MHz, $CDCl_3$) 3.31 (6H, s), 5.52 (1H, s), 6.37-6.39 (1H, m), 6.46-6.49 (1H, m), 6.54-6.56 (1H, m), 6.62-6.65 (1H, m), 7.37-7.40 (2H, m), 7.48-7.50 (1H, m), 7.62-7.63 (2H, m); $\delta_C$ (100 MHz, $CDCl_3$) 53.0, 101.5, 121.1, 126.5, 127.0, 128.4, 131.5, 132.2, 135.0, 135.3 (s), 136.2, 136.8 (s), 146.3 (s); HRMS (ESI) *m/z* calcd for $C_{14}H_{13}O$ $[M+H-OCH_4]^+$ 197.0966; found: 197.0959.

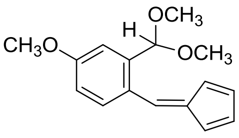


**Fulvene 24b:** eluent for column chromatography: hexanes/diethyl ether (9:1 v/v); (71%); red oil; $\nu_{max}$ (Neat)/$cm^{-1}$ 1600 (vs), 1493 (vs), 1305 (vs); $\delta_H$ (400 MHz, $CDCl_3$) 3.33 (6H, s), 3.87 (3H, s), 5.55 (1H, s), 6.37-6.39 (1H, m), 6.53-6.54 (2H, m), 6.64-6.65 (1H, m), 6.93 (1H, dd, *J* = 2.4, 8.4 Hz),

7.23 (1H, d, *J* = 2.3 Hz), 7.52 (1H, d, *J* = 8.4 Hz), 7.60 (1H, s); $\delta_C$ (100 MHz, $CDCl_3$) 53.0, 55.4, 101.2, 112.6, 114.0, 120.8, 126.6, 127.8 (s), 130.7, 133.8, 134.6, 136.0, 138.7 (s), 145.0 (s), 160.1 (s); HRMS (ESI) *m/z* calcd for $C_{15}H_{10}O_2$ $[M+H-OCH_4]^+$ 227.1062; found: 227.1067.

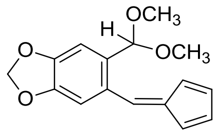


**Fulvene 24c:** eluent for column chromatography: hexanes/diethyl ether (9:1 v/v); (81%); red oil; $\nu_{max}$ (Neat)/cm$^{-1}$ 1601 (m), 1502 (s), 1482 (vs); $\delta_H$ (400 MHz, $CDCl_3$) 3.30 (6H, s), 5.50 (1H, s), 6.01 (2H, s), 6.34-6.36 (1H, m), 6.51-6.53 (2H, m), 6.63-6.67 (1H, m), 7.04 (1H, s), 7.16 (1H, s), 7.52 (1H, s); $\delta_C$ (100 MHz, $CDCl_3$) 52.9, 100.7, 101.6, 107.6, 111.8, 120.6, 126.8, 129.3 (s), 131.0, 132.2 (s), 135.0, 135.6, 145.3 (s), 147.5 (s), 148.2 (s); HRMS (ESI) *m/z* calcd for $C_{15}H_{13}O_3$ $[M+H-OCH_4]^+$ 241.0865; found: 241.0867.

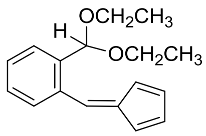


**Fulvene 24d:** eluent for column chromatography: hexanes/diethyl ether (9:1 v/v); (74%); red oil; $\nu_{max}$ (Neat)/cm$^{-1}$ 1626 (m), 1481 (m), 1373 (m); $\delta_H$ (400 MHz, $CDCl_3$) 1.21 (6H, t, *J* = 7.2 Hz), 3.50-3.63 (4H, m), 5.67 (1H, s), 6.39-6.40 (1H, m), 6.47-6.49 (1H, m), 6.55-6.57 (1H, m), 6.63-6.65 (1H, m), 7.37-7.40 (2H, m), 7.48-7.50 (1H, m), 7.67-7.69 (2H, m); $\delta_C$ (100 MHz, $CDCl_3$) 15.2, 61.3, 99.7, 121.2, 126.4, 126.7, 128.3, 128.5, 131.4, 132.1, 134.9, 135.2 (s), 136.5, 137.8 (s), 146.1 (s); HRMS (ESI) *m/z* calcd for $C_{15}H_{15}O$ $[M+H-OC_2H_6]^+$ 211.1123; found: 211.1124.

**Procedure for the preparation of benz[*f*]indenes 5, 6, 25 and 26**

A solution of the fulvene **3** or **24** (1 mmol) in dimethylsulfoxide (5 mL) was heated by microwave at 120 °C and at 120 W for 20–40 min. Then, the reaction mixture was poured into water (10 mL) and extracted with dichloromethane (3 × 10 mL). The organic layer was washed with water (3 × 10 mL) and dried over anhydrous $MgSO_4$. The solvent was evaporated and the resulting material was purified by column chromatography on silica gel.

**Benz[*f*]indenes 5a and 6a:** eluent for column chromatography: hexanes/diethyl ether (4:1 v/v); (34%).

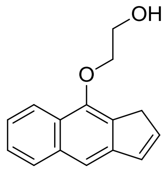


**9-(2-Hydroxy)ethoxybenz[*f*]indene (5a):** yellow oil; $\nu_{max}$ (Neat)/cm$^{-1}$ 3384 (s), 1683 (m), 1579 (s); $\delta_H$ (300 MHz, $CDCl_3$) 2.36 (1H, br s), 3.60 (2H, t, *J*

= 2.0 Hz), 4.05 (2H, t, $J$ = 4.4 Hz), 4.33 (2H, t, $J$ = 4.4 Hz), 6.61 (1H, dt, $J$ = 2.0, 5.6 Hz), 6.95 (1H, dt, $J$ = 2.0, 5.6 Hz), 7.44-7.49 (2H, m), 7.60 (1H, s), 7.85-7.87 (1H, m), 8.19-8.22 (1H, m); $\delta_C$ (75 MHz, $CDCl_3$) 36.5, 62.5, 73.8, 115.1, 121.8, 124.9, 125.7, 126.1 (s), 128.1, 128.7 (s), 132.2, 134.9 (s), 135.3, 145.2 (s), 149.4 (s); HRMS (ESI) *m/z* calcd for $C_{15}H_{15}O_2$ $[M+H]^+$ 227.1067; found: 227.1067.

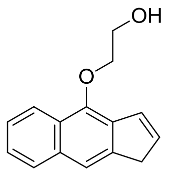


**4-(2-Hydroxy)ethoxybenz[*f*]indene (6a):** yellow oil; $\nu_{max}$ (Neat)/$cm^{-1}$ 3384 (m), 1579 (m), 1398 (m); $\delta_H$ (300 MHz, $CDCl_3$) 2.09 (1H, br s), 3.56-3.57 (2H, m), 4.05 (2H, t, $J$ = 5.0 Hz), 4.30 (2H, t, $J$ = 5.0 Hz), 6.59 (1H, dt, $J$ = 2.1, 5.7 Hz), 7.16-7.19 (1H, m), 7.41-7.50 (2H, m), 7.69 (1H, s), 7.82-7.85 (1H, m), 8.20-8.23 (1H, m); $\delta_C$ (75 MHz, $CDCl_3$) 38.7, 62.5, 75.8, 118.6, 121.8, 125.2, 125.3, 127.1 (s), 128.0, 128.5, 133.3 (s) 133.4 (s), 135.0, 143.0 (s), 146.6 (s); HRMS (ESI) *m/z* calcd for $C_{15}H_{15}O_2$ $[M+H]^+$ 227.1067; found: 227.1068.

**Benz[*f*]indenes 5b and 6b:** eluent for column chromatography: hexanes/diethyl ether (9:1 v/v), (30%).

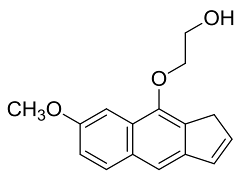


**9-(2-Hydroxy)ethoxy-7-methoxybenz[*f*]indene (5b):** yellow oil; $\nu_{max}$ (Neat)/$cm^{-1}$ 3454 (m), 1618 (vs), 1500 (s); $\delta_H$ (300 MHz, $CDCl_3$) 2.00 (1H, br s), 3.59 (2H, t, $J$ = 1.8 Hz), 3.94 (3H, s), 4.05 (2H, t, $J$ = 5.0 Hz), 4.30 (2H, t, $J$ = 5.0 Hz), 6.53-6.56 (1H, m), 6.91-6.94 (1H, m), 7.13 (1H, dd, $J$ = 2.7, 9.0 Hz), 7.53 (1H, s), 7.54 (1H, d, $J$ = 2.7 Hz), 7.76 (1H, d, $J$ = 9.0 Hz); $\delta_C$ (75 MHz, $CDCl_3$) 36.4, 55.4, 62.6, 73.6, 100.6, 115.1, 118.1, 127.3 (s), 129.6, 129.9 (s), 130.3 (s), 132.2, 134.0, 143.1 (s), 148.7 (s), 157.4 (s); HRMS (ESI) *m/z* calcd for $C_{16}H_{17}O_3$ $[M+H]^+$ 257.1172; found: 257.1179.

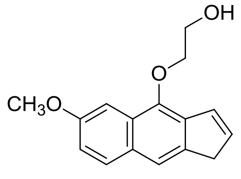


**4-(2-Hydroxy)ethoxy-6-methoxybenz[*f*]indene (6b):** yellow oil; $\nu_{max}$ (Neat)/$cm^{-1}$ 3423 (s), 1617 (vs), 1501 (s); $\delta_H$ (300 MHz, $CDCl_3$) 1.86 (1H, br s), 3.53-3.54 (2H, m), 3.95 (3H, s), 4.05 (2H, t, $J$ = 4.8 Hz), 4.28 (2H, t, $J$ = 4.8 Hz), 6.58-6.61 (1H, m), 7.11 (1H, dd, $J$ = 2.7, 9.0 Hz), 7.15-7.17 (1H, m), 7.54 (1H, d, $J$ = 2.7 Hz), 7.62 (1H, s), 7.73 (1H, d, $J$ = 9.0 Hz); $\delta_C$ (75 MHz, $CDCl_3$) 38.6, 55.4, 62.6, 75.5, 100.2, 117.8, 118.4, 128.1 (s), 128.4, 128.9 (s), 129.5, 134.0 (s), 135.2, 140.5 (s), 145.9 (s), 157.5 (s); HRMS (ESI) *m/z* calcd for $C_{16}H_{17}O_3$ $[M+H]^+$ 257.1172; found: 257.1182.

**Benz[*f*]indenes 5c and 6c:** eluent for column chromatography: hexanes/diethyl ether (9:1 v/v), (45%).

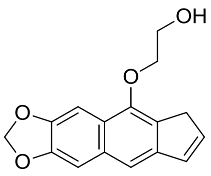


**9-(2-Hydroxy)ethoxy-6,7-(methylenedioxy)benz[*f*]indene (5c):** yellow oil; $\nu_{max}$ (Neat)/cm$^{-1}$ 3317 (m), 1498 (s), 1298 (s); $\delta_H$ (400 MHz, $CDCl_3$) 2.11 (1H, br s), 3.56 (2H, t, *J* = 2.0 Hz), 4.04 (2H, t, *J* = 4.8 Hz), 4.29 (2H, t, *J* = 4.8 Hz), 6.04 (2H, s), 6.55 (1H, dt, *J* = 2.0, 5.6 Hz), 6.89 (1H, dt, *J* = 2.0, 5.6 Hz), 7.14 (1H, s), 7.43 (1H, s), 7.51 (1H, s); $\delta_C$ (100 MHz, $CDCl_3$) 36.4, 62.5, 73.6, 98.7, 101.1, 104.3, 114.3, 122.7 (s), 127.9 (s), 131.8 (s), 132.1, 134.3, 144.0 (s), 147.2 (s), 147.5 (s), 149.2 (s); HRMS (ESI) *m/z* calcd for $C_{16}H_{15}O_4$ [M+H]$^+$ 271.0965; found: 271.0973.

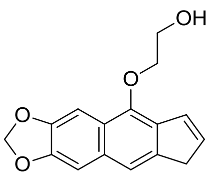


**4-(2-Hydroxy)ethoxy-6,7-(methylenedioxy)benz[*f*]indene (6c):** yellow oil; $\nu_{max}$ (Neat)/cm$^{-1}$ 3503 (m), 1498 (m), 1462 (vs); $\delta_H$ (400 MHz, $CDCl_3$) 2.01 (1H, br s), 3.50-3.52 (2H, m), 4.04 (2H, t, *J* = 4.8 Hz), 4.24 (2H, t, *J* = 4.8 Hz), 6.04 (2H, s), 6.52 (1H, dd, *J* = 2.4, 5.6 Hz), 7.11-7.12 (2H, m), 7.51-7.52 (2H, m); $\delta_C$ (100 MHz, $CDCl_3$) 38.6, 62.5, 75.6, 98.5, 101.1, 104.2, 117.9, 123.6 (s), 128.4, 130.3 (s), 132.3 (s), 134.0, 141.8 (s), 146.4 (s), 147.2 (s), 147.4 (s); HRMS (ESI) *m/z* calcd for $C_{16}H_{15}O_4$ [M+H]$^+$ 271.0965; found: 271.0972.

**Benz[*f*]indenes 5d and 6d:** eluent for column chromatography: hexanes/diethyl ether (4:1 v/v), (57%).

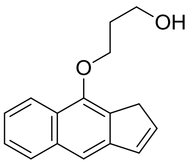


**9-(3-Hydroxy)propoxybenz[*f*]indene (5d):** white oil; $\nu_{max}$ (Neat)/cm$^{-1}$ 3373 (s), 1692 (m), 1579 (vs); $\delta_H$ (300 MHz, $CDCl_3$) 2.02 (1H, br s), 2.17 (2H, quint, *J* = 5.7 Hz), 3.63 (2H, t, *J* = 2.1 Hz), 4.03 (2H, t, *J* = 6.0 Hz), 4.40 (2H, t, *J* = 6.0 Hz), 6.61 (1H, dt, *J* = 2.1, 5.5 Hz), 6.95 (1H, dt, *J* = 2.1, 5.5 Hz), 7.42-7.48 (2H, m), 7.57 (1H, s), 7.83-7.86 (1H, m), 8.16-8.19 (1H, m); $\delta_C$ (75 MHz, $CDCl_3$) 33.1, 36.6, 61.0, 70.9, 114.9, 121.9, 124.8, 125.6, 126.2 (s), 128.0, 128.3 (s), 132.2, 134.9 (s), 135.3, 145.3 (s), 149.7 (s); HRMS (ESI) *m/z* calcd for $C_{16}H_{17}O_2$ [M+H]$^+$ 241.1223; found: 241.1230.

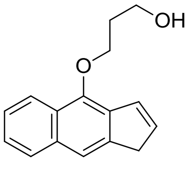


**4-(3-Hydroxy)propoxybenz[*f*]indene (6d)** white oil; $\nu_{max}$ (Neat)/cm$^{-1}$ 3386 (m), 1581 (m), 1363 (vs); $\delta_H$ (300 MHz, $CDCl_3$) 2.18 (2H, quint, *J* = 6.0 Hz), 2.30 (1H, br s), 3.55 (2H, br s), 4.04 (2H, t, *J* = 6.0 Hz), 4.34

(2H, t, *J* = 6.0 Hz), 6.58 (1H, dt, *J* = 2.1, 5.4 Hz), 7.17-7.19 (1H, m), 7.42-7.51 (2H, m), 7.67 (1H, s), 7.82-7.85 (1H, m), 8.19-8.22 (1H, m); $\delta_C$ (75 MHz, $CDCl_3$) 33.1, 38.6, 61.0, 73.0, 118.3, 121.9, 125.0, 125.2, 127.0 (s), 127.8, 128.6, 133.0 (s), 133.3 (s), 134.7, 142.9 (s), 146.9 (s); HRMS (ESI) *m/z* calcd for $C_{16}H_{17}O_2$ $[M+H]^+$ 241.1223; found: 241.1231.

**Benz[*f*]indenes 5e and 6e**: eluent for column chromatography: hexanes/diethyl ether (4:1 v/v), (46%).

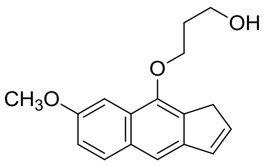


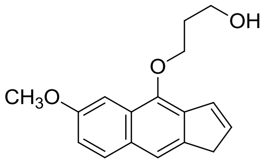


**9-(3-Hydroxy)propoxy-7-methoxybenz[*f*]-indene (5e) and 4-(3-hydroxy)propoxy-6-methoxybenz[*f*]indene (6e):** white oil; $\nu_{max}$ (Neat)/cm$^{-1}$ 3417 (m), 1617 (vs), 1500 (vs); $\delta_H$ (300 MHz, $CDCl_3$) 2.10-2.21 (4H, m)$_{minor+major}$, 2.49 (2H, br s)$_{minor+major}$, 3.50-3.51 (2H, m)$_{minor+major}$, 3.57 (2H, t, *J* = 2.1 Hz)$_{major}$, 3.94 (3H, s)$_{major}$, 3.95 (3H, s)$_{minor}$, 4.00-4.03 (4H, m)$_{minor+major}$, 4.28-4.35 (4H, m)$_{minor+major}$, 6.51-6.58 (2H, m)$_{minor+major}$, 6.91 (1H, dt, *J* = 2.1, 5.4 Hz)$_{major}$, 7.10-7.16 (2H, m)$_{minor+major}$, 7.49-7.51 (4H, m)$_{minor+major}$, 7.57 (1H, s)$_{minor}$, 7.71 (1H, d, *J* = 9.0 Hz)$_{minor}$, 7.73 (1H, d, *J* = 9.0 Hz)$_{major}$; $\delta_C$ (75 MHz, $CDCl_3$) 33.1, 36.4, 38.5, 55.3, 60.7, 60.8, 70.3, 72.5, 100.3, 100.5, 114.7, 117.6, 117.8, 118.1, 127.1 (s), 127.9 (s), 128.5, 128.8 (s), 129.2 (s), 129.3, 129.5, 130.1 (s), 132.1, 133.6 (s), 133.9, 134.9, 140.4 (s), 143.1 (s), 146.0 (s), 148.8 (s), 157.1 (s), 157.3 (s); HRMS (ESI) *m/z* calcd for $C_{17}H_{18}NaO_3$ $[M+Na]^+$ 293.1154; found: 293.1153.

**Benz[*f*]indenes 5f and 6f:** eluent for column chromatography: hexanes/diethyl ether (4:1 v/v), (53%).

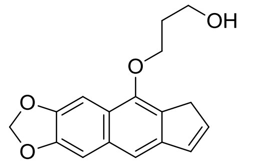


**9-(3-Hydroxy)propoxy-6,7-(methylenedioxy)benz[*f*]indene (5f):** white oil; $\nu_{max}$ (Neat)/cm$^{-1}$ 3382 (m), 1617 (m), 1497 (s); $\delta_H$ (300 MHz, $CDCl_3$) 2.09-2.18 (3H, m), 3.57 (2H, t, *J* = 1.8 Hz), 4.00 (2H, t, *J* = 6.0 Hz), 4.33 (2H, t, *J* = 6.0 Hz), 6.02 (2H, s), 6.53 (1H, dt, *J* = 1.8, 5.7 Hz), 6.88 (1H, dt, *J* = 1.8, 5.7 Hz), 7.12 (1H, br s), 7.40 (1H, s), 7.47 (1H, s); $\delta_C$ (75 MHz, $CDCl_3$) 33.1, 36.5, 60.9, 70.6, 98.8, 101.0, 104.2, 122.7 (s), 127.6 (s), 131.8 (s), 132.1, 134.3, 144.1 (s), 147.0 (s), 147.4 (s), 149.5 (s); HRMS (ESI) *m/z* calcd for $C_{17}H_{16}NaO_4$ $[M+Na]^+$ 307.0946; found: 307.0950.

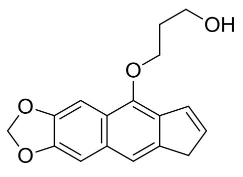

**4-(3-Hydroxy)propoxy-6,7-(methylenedioxy)benz[*f*]indene (6f):** white oil; $\nu_{max}$ (Neat)/cm$^{-1}$ 3384 (m), 1498 (s), 1460 (vs); $\delta_H$ (300 MHz, $CDCl_3$) 1.86 (1H, br s), 2.16 (2H, quint, *J* = 5.7 Hz), 3.49-3.51 (2H, m), 4.03 (2H, t, *J* = 5.7 Hz), 4.28 (2H, t, *J* = 5.7 Hz), 6.03 (2H, s), 6.51 (1H, dt, *J* = 2.1, 5.7 Hz), 7.10-7.12 (2H, m), 7.48 (1H, s), 7.50 (1H, s); $\delta_C$ (75 MHz, $CDCl_3$) 33.1, 38.6, 61.1, 72.9, 98.7, 101.1, 104.1, 117.8, 123.6 (s), 128.5, 130.3 (s), 132.2 (s), 133.8, 141.8 (s), 146.7 (s), 147.2 (s), 147.3 (s); HRMS (ESI) *m/z* calcd for $C_{17}H_{16}NaO_4$ [M+Na]$^+$ 307.0946; found: 307.0947.

**Benz[*f*]indenes 25a and 26a:** eluent for column chromatography: hexanes/diethyl ether (4:1 v/v), (54%).

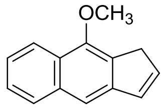

**9-Methoxybenz[*f*]indene (25a):** white oil; $\nu_{max}$ (Neat) /cm$^{-1}$ 1632 (m), 1579 (vs), 1499 (s); $\delta_H$ (300 MHz, $CDCl_3$) 3.64 (2H, t, *J* = 2.1 Hz), 4.10 (3H, s), 6.62 (1H, dt, *J* = 1.5, 5.4 Hz), 6.95 (1H, dt, *J* = 1.5, 5.7 Hz), 7.44-7.47 (2H, m), 7.57 (1H, s), 7.83-7.86 (1H, m), 8.19-8.22 (1H, m); $\delta_C$ (75 MHz, $CDCl_3$) 36.6, 60.2, 114.7, 122.1, 124.7, 125.7, 126.1 (s), 127.7 (s), 127.9, 132.3, 134.9 (s), 135.3, 145.4 (s), 150.9 (s); HRMS (ESI) *m/z* calcd for $C_{14}H_{13}O$ [M+H]$^+$ 197.0966; found: 197.0960.

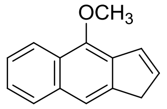

**4-Methoxybenz[*f*]indene (26a):** $\delta_H$ (300 MHz, $CDCl_3$) 3.57-3.59 (2H, br s), 4.13 (3H, s), 6.60 (1H, dt, *J* = 1.5, 4.2 Hz), 7.22-7.25 (1H, m), 7.47-7.52 (2H, m), 7.69 (1H, s), 7.85-7.89 (1H, m), 8.24-8.28 (1H, m); $\delta_C$ (75 MHz, $CDCl_3$) 38.5, 62.2, 118.1, 122.0, 124.9, 125.3, 126.9 (s), 127.8, 128.7, 132.4 (s), 133.4 (s), 134.5, 143.1 (s), 148.1 (s). HRMS (ESI) *m/z* calcd for $C_{14}H_{13}O$ [M+H]$^+$ 197.0966; found: 197.0962.

**Benz[*f*]indenes 25b and 26b:** eluent for column chromatography: hexanes/diethyl ether (4:1 v/v), (42%).

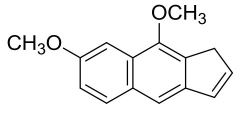

**7,9-Dimethoxybenz[*f*]indene (25b):** white oil; $\nu_{max}$ (Neat)/cm$^{-1}$ 1617 (s), 1501 (m), 1465 (m); $\delta_H$ (400 MHz, $CDCl_3$) 3.64 (2H, t, *J* = 2.0 Hz), 3.96 (3H, s), 4.11 (3H, s), 6.54-6.57 (1H, m), 6.92-6.94 (1H, m), 7.13 (1H, dd, *J* = 2.8, 8.8 Hz), 7.51 (1H, d, *J* = 2.8 Hz), 7.51 (1H, s), 7.75 (1H, d, *J* = 8.8 Hz); $\delta_C$ (100 MHz, $CDCl_3$) 36.5, 55.4, 59.9, 100.5, 114.5, 118.1, 127.0 (s), 128.5 (s), 129.4, 130.2 (s), 132.2, 134.0, 143.2 (s), 150.1 (s), 157.2 (s); HRMS (ESI) *m/z* calcd for $C_{15}H_{15}O_2$ [M+H]$^+$ 227.1072; found: 227.1062.

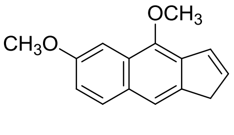


**4,6-Dimethoxybenz[*f*]indene (26b):** white oil; $\nu_{max}$ (Neat)/cm$^{-1}$ 1617 (vs), 1502 (s), 1466 (s); $\delta_H$ (300 MHz, $CDCl_3$) 3.52-3.54 (2H, m), 3.96 (3H, s), 4.09 (3H, s), 6.58 (1H, dt, *J* = 2.1, 5.7 Hz), 7.11 (1H, dd, *J* = 2.7, 9.0 Hz), 7.17-7.20 (1H, m), 7.51 (1H, d, *J* = 2.7 Hz), 7.60 (1H, s), 7.73 (1H, d, *J* = 9.0 Hz); $\delta_C$ (75 MHz, $CDCl_3$) 38.5, 55.4, 61.9, 100.3, 117.8, 117.9, 127.8 (s), 128.7, 128.8 (s), 129.3, 133.1 (s), 134.7, 140.6 (s), 147.4 (s), 157.4 (s); HRMS (ESI) *m/z* calcd for $C_{15}H_{15}O_2$ $[M+H]^+$ 227.1072; found: 227.1068.

**Benz[*f*]indenes 25c and 26c:** eluent for column chromatography: hexanes/diethyl ether (4:1 v/v), (47%).

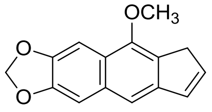


**9-Methoxy-6,7-(methylenedioxy)benz[*f*]indene (25c):** white oil; $\nu_{max}$ (Neat)/cm$^{-1}$ 1499 (m), 1464 (vs), 1299 (m); $\delta_H$ (400 MHz, $CDCl_3$) 3.60 (2H, t, *J* = 2.4 Hz), 4.06 (3H, s), 6.03 (2H, s), 6.55 (1H, dt, *J* = 2.0, 5.6 Hz), 6.90 (1H, dt, *J* = 2.0, 5.6 Hz), 7.13 (1H, s), 7.41 (1H, s), 7.50 (1H, s); $\delta_C$ (100 MHz, $CDCl_3$) 36.5, 60.1, 98.9, 101.1, 104.1, 114.2, 122.6 (s), 127.1 (s), 131.8 (s), 132.1, 134.3, 144.2 (s), 147.0 (s), 147.5 (s), 150.6 (s); HRMS (ESI) *m/z* calcd for $C_{15}H_{13}O_3$ $[M+H]^+$ 241.0865; found: 241.0851.

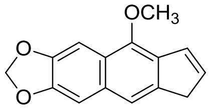


**4-Methoxy-6,7-(methylenedioxy)benz[*f*]indene (26c):** white oil; $\nu_{max}$ (Neat)/cm$^{-1}$ 1499 (m), 1465 (vs), 1300 (m); $\delta_H$ (300 MHz, $CDCl_3$) 3.50-3.51 (2H, m), 4.03 (3H, s), 6.03 (2H, s), 6.51 (1H, dt, *J* = 2.1, 5.7 Hz), 7.11-7.14 (2H, m), 7.50 (2H, m),; $\delta_C$ (75 MHz, $CDCl_3$) 38.6, 62.1, 98.8, 101.1, 104.1, 114.1 (s), 117.6, 123.5 (s), 128.6, 130.3 (s), 131.7 (s), 133.6, 141.9 (s), 147.3 (s), 148.0 (s); HRMS (ESI) *m/z* calcd for $C_{15}H_{13}O_3$ $[M+H]^+$ 241.0865; found: 241.0852.

**Benz[*f*]indenes 25d and 26d:** eluent for column chromatography: hexanes/diethyl ether (4:1 v/v), (60%).

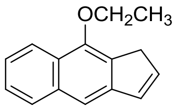


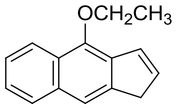


**9-Ethoxybenz[*f*]indene (25d) and 4-ethoxybenz[*f*]indene (26d):** white oil; $\nu_{max}$ (Neat)/cm$^{-1}$ 1607 (m), 1580 (vs), 1418 (vs); $\delta_H$ (300 MHz, $CDCl_3$) 1.55 (6H, t, *J* = 7.2 Hz)$_{minor+major}$, 3.58 (2H, br s)$_{minor}$, 3.64 (6H, t, *J* = 2.1 Hz)$_{major}$, 4.28-4.38 (4H, m)$_{minor+major}$, 6.56-6.60 (1H, m)$_{minor}$, 6.61-6.65 (1H, m)$_{major}$, 6.97 (1H, dt, *J* = 1.8, 5.4 Hz)$_{major}$, 7.17-7.20 (1H, m)$_{minor}$, 7.45-7.50 (4H, m)$_{minor+major}$, 7.59 (1H, s)$_{major}$, 7.68 (1H,

s)$_{minor}$, 7.83-7.88 (2H, m)$_{minor+major}$, 8.21-8.28 (2H, m)$_{minor+major}$; $\delta_C$ (75 MHz, $CDCl_3$) 16.0, 16.1, 36.7, 38.6, 68.3, 70.5, 114.6, 118.0, 122.3, 124.6, 124.8, 125.2, 125.5, 126.6 (s), 127.4 (s), 127.8, 127.9, 128.3 (s), 129.0, 132.2, 133.1 (s), 133.4 (s), 134.2, 134.9 (s), 135.2, 143.0 (s), 145.3 (s), 147.2 (s), 150.0 (s); HRMS (ESI) *m/z* calcd for $C_{15}H_{15}O$ $[M+H]^+$ 211.1123; found: 211.1124.

**Procedure for the preparation of 2-(1,3-dioxolan-2-yl)benzaldehyde-formyl-*d***

*n*-BuLi (12.3 mL, 2.6 M in hexane) was added dropwise to a solution of 2-(2-bromophenyl)-1,3-dioxolane (3.0 g, 12.3 mmol) in anhydrous tetrahydrofuran (50 mL), at −78 °C under an atmosphere of nitrogen. The mixture was stirred at −78 °C for 30 min. Then, a solution of *N*-formylpiperidine-$d_7$ (1.4 gr, 12.3 mmol) in tetrahydrofuran (10 mL) was added dropwise. The mixture was stirred at −78 °C for 15 min, warmed to room temperature and stirred for 3 h. Then, the reaction was quenched with the addition of water (25 mL) and extracted with ethyl acetate (2 × 30 mL). The combined organic layers were washed with water (2 × 100 mL) and dried over anhydrous $MgSO_4$. The solvent was removed under reduced pressure and the resulting oil was purified by silica gel column chromatography using hexanes/diethyl ether (3:2 v/v) as eluent.

**2-(1,3-Dioxolan-2-yl)benzaldehyde formyl-*d*:** (72%); yellow oil; $\nu_{max}$ (Neat)/cm$^{-1}$ 1673 (vs), 1599 (vs), 1407 (m); $\delta_H$ (400 MHz, $CDCl_3$) 4.07-4.16 (4H, m), 6.39 (1H, s), 7.52 (1H, t, $J$ = 7.6 Hz), 7.61 (1H, t, $J$ = 7.6 Hz), 7.72 (1H, dd, $J$ = 7.6, 1.2 Hz), 7.93 (1H, dd, $J$ = 7.6, 1.2 Hz); $\delta_C$ (100 MHz, $CDCl_3$) 65.4, 101.2, 127.0, 129.5, 130.1, 133.7, 134.4 (t, $J_{CD}$ = 3.5 Hz) (s), 139.1 (s), 191.5 (t, $J_{CD}$ = 27.2 Hz); HRMS (ESI) *m/z* calcd for $C_{10}H_{10}DO_3$ $[M+H]^+$ 180.0771; found: 180.0777.

**Procedure for the preparation of the fulvenes 14 and 20**

To a solution of the appropriate benzaldehyde (3 mmol) in anhydrous methanol (15 mL) were added cyclopentadiene (6 mmol) and pyrrolidine (6 mmol). The reaction mixture was stirred at room temperature overnight. Then, the reaction mixture was acidified with acetic acid (1 mL), diluted with water (40 mL) and extracted with diethyl ether (3 × 20 mL). The organic layer was washed with brine (2 × 10 mL), dried over anhydrous $MgSO_4$, filtered and evaporated under reduced pressure. The resulting residue was purified by silica gel column chromatography.

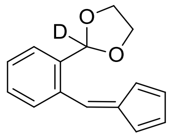

**Fulvene 14:** eluent for column chromatography: hexanes/diethyl ether (9:1 v/v); (77%); red oil; $\nu_{max}$ (Neat)/cm$^{-1}$ 1626 (w), 1482 (m), 1229 (s); $\delta_H$ (400 MHz, $CDCl_3$) 4.03-4.17 (4H, m), 6.37 (1H, td, $J$ = 2.0, 5.2 Hz), 6.48-6.50 (1H, m ), 6.55-6.57 (1H, m), 6.63-6.65 (1H, m), 7.38-7.44 (2H, m), 7.50-7.52 (1H, m), 7.63-7.65 (1H, m); $\delta_C$ (100 MHz, $CDCl_3$) 65.5, 101.6 (t, $J_{CD}$ = 25.5 Hz), 121.2, 126.2, 126.3, 128.8, 129.0, 131.7, 132.2, 135.1, 135.7 (s), 135.8, 136.4 (s), 146.6 (s); HRMS (ESI) *m/z* calcd for $C_{15}H_{14}DO_2$ $[M+H]^+$ 228.1135; found: 228.1136.

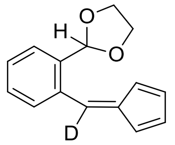

**Fulvene 20:** eluent for column chromatography: hexanes/diethyl ether (9:1 v/v); (77%); red oil; $\nu_{max}$ (Neat)/cm$^{-1}$ 1616 (m), 1484 (m), 1361 (s); $\delta_H$ (300 MHz, $CDCl_3$) 4.02-4.18 (4H, m), 6.02 (1H, s), 6.36-6.38 (1H, m ), 6.47-6.50 (1H, m), 6.54-6.56 (1H, m), 6.63-6.65 (1H, m), 7.38-7.42 (2H, m), 7.49-7.53 (1H, m), 7.62-7.65 (1H, m); $\delta_C$ (75 MHz, $CDCl_3$) 65.4, 102.1, 121.2, 126.3, 126.4, 128.7, 129.0, 131.6, 132.2, 135.1, 135.7 (s), 136.6 (s), 146.5 (s); HRMS (ESI) *m/z* calcd for $C_{15}H_{13}DO_2$ $[M+H]^+$ 228.1135; found: 228.1138.

**Procedure for the preparation of the benz[*f*]indenes 18, 19, 21 and 22**

A solution of the fulvene **14** or **20** (1 mmol) in dimethylsulfoxide (5 mL) was heated by microwave at 120 °C and at 120 W for 40 min. Then, the reaction mixture was poured into water (10 mL) and extracted with dichloromethane (3 × 10 mL). The organic layer was washed with water (3 × 10 mL) and dried over anhydrous $MgSO_4$. The solvent was evaporated and the resulting material was purified by column chromatography on silica gel.

**Benz[*f*]indenes 18 and 19:** eluent for column chromatography: hexanes/diethyl ether (7:3 v/v), (43%).

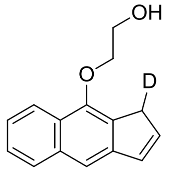

**9-(2-Hydroxy)ethoxybenz[*f*]indene-1-*d* (18):** white oil; $\nu_{max}$ (Neat)/cm$^{-1}$ 3383 (vs), 1579 (s), 1494 (m); $\delta_H$ (400 MHz, $CDCl_3$) 2.43 (1H, br s), 3.59-3.61 (1H, m), 4.06 (2H, t, $J$ = 4.1 Hz), 4.34 (2H, t, $J$ = 4.1 Hz), 6.61 (1H, dd, $J$ = 2.4, 5.6 Hz), 6.96 (1H, dd, $J$ = 2.4, 5.6 Hz), 7.45-7.48 (2H, m), 7.59 (1H, s), 7.84-7.87 (1H, m), 8.19-8.22 (1H, m); $\delta_C$ (75 MHz, $CDCl_3$) 36.2 (t, $J_{CD}$ = 19.7 Hz), 62.5, 73.9, 115.1, 121.8, 124.9, 125.7, 126.2 (s), 128.1, 128.6 (s), 132.3, 134.9 (s), 135.3, 145.3 (s), 149.5 (s); HRMS (ESI) *m/z* calcd for $C_{15}H_{14}DO_2$ $[M+H]^+$ 228.1135; found: 228.1140.

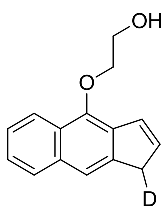


**4-(2-Hydroxy)ethoxybenz[*f*]indene-1-*d* (19):** white oil; $\nu_{max}$ (Neat)/cm$^{-1}$ 3384 (vs), 1579 (m), 1499 (m); $\delta_H$ (400 MHz, $CDCl_3$) 2.36-2.38 (1H, m), 3.50-3.57 (1H, m), 4.04-4.08 (2H, m), 4.29-4.31(2H, m), 6.59 (1H, dd, *J* = 2.0, 5.6 Hz), 7.18 (1H, dd, *J* = 2.0, 5.6 Hz), 7.42-7.50 (2H, m), 7.68 (1H, s), 7.83-7.85 (1H, m), 8.21-8.23 (1H, m); $\delta_C$ (100 MHz, $CDCl_3$) 38.4 (t, $J_{CD}$ = 19.6 Hz), 62.6, 75.8, 118.6, 121.9, 125.2, 125.4, 127.1 (s), 128.0, 128.6, 133.3 (s), 133.4 (s), 135.0, 143.0 (s), 146.6 (s); HRMS (ESI) *m/z* calcd for $C_{15}H_{14}DO_2$ $[M+H]^+$ 228.1135; found: 228.1139.

**Benz[*f*]indenes 21 and 22:** eluent for column chromatography: hexanes/diethyl ether (7:3 v/v), (43%).

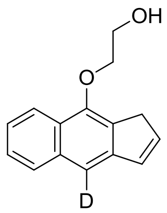


**9-(2-Hydroxy)ethoxybenz[*f*]indene-4-*d* (21):** white oil; $\nu_{max}$ (Neat)/cm$^{-1}$ 3384 (vs), 1578 (m), 1418 (vs); $\delta_H$ (400 MHz, $CDCl_3$) 2.39 (1H, br s), 3.60 (2H, t, *J* = 2.0 Hz), 4.05 (2H, t, *J* = 4.0 Hz), 4.32 (2H, t, *J* = 4.0 Hz), 6.61 (1H, dt, *J* = 2.0, 5.6 Hz), 6.95 (1H, dt, *J* = 2.0, 5.6 Hz), 7.43-7.48 (2H, m), 7.84-7.87 (1H, m), 8.19-8.22 (1H, m); $\delta_C$ (100 MHz, $CDCl_3$) 36.5, 62.5, 114.8 (t, $J_{CD}$ = 24.2 Hz), 121.8, 124.9, 125.7, 126.1 (s), 128.0, 128.7 (s), 132.1, 134.8 (s), 135.3, 145.1 (s), 149.4 (s); HRMS (ESI) *m/z* calcd for $C_{15}H_{14}DO_2$ $[M+H]^+$ 228.1135; found: 228.1140.

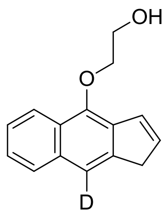


**4-(2-Hydroxy)ethoxybenz[*f*]indene-9-*d* (22):** white oil; $\nu_{max}$ (Neat)/cm$^{-1}$ 3384 (vs), 1580 (m), 1345 (vs); $\delta_H$ (400 MHz, $CDCl_3$) 2.28 (1H, br s), 3.57 (2H, t, *J* = 2.0 Hz), 4.06 (2H, t, *J* = 4.8 Hz), 4.30 (2H, t, *J* = 4.8 Hz), 6.59 (1H, dt, *J* = 2.4, 5.6 Hz), 7.18 (1H, dt, *J* = 2.4, 5.6 Hz), 7.42-7.50 (2H, m), 7.83-7.85 (1H, m), 8.21-8.24 (1H, m); $\delta_C$ (100 MHz, $CDCl_3$) 38.6, 62.5, 75.8, 118.2 (t, $J_{CD}$ = 24.1 Hz), 121.8, 125.2, 125.3, 127.0 (s), 127.9, 128.5, 133,2 (s), 133.3 (s), 135.0, 142.9 (s), 146.6 (s); HRMS (ESI) *m/z* calcd for $C_{15}H_{14}DO_2$ $[M+H]^+$ 228.1135; found: 228.1139.

**References:**

[1] Hartman, G. D.; Phillips, B. T.; Halczenko, W. *J. Org. Chem.* **1985**, *50*, 2423-2427.

[2] Remy, D. C.; King, S. W.; Cochran, D. *J. Org. Chem.* **1985**, *50*, 4120-4125.

[3] Dumoulin, D.; Lebrun, S.; Deniau, E.; Couture, A.; Grandclaudon, P. *Eur. J. Org. Chem.* **2009**, 3741-3752.

[4] Moody, C.J.; Warrellow, G. J. *J. Chem. Soc., Perkin Trans. 1* **1990**, 2929-2936.

[5] Meegalla, S. K.; Rodrigo, R. *J. Org. Chem.* **1991**, ***56***, 1882-1888.

[6] Friedrichsen, W.; Koening, B. M.; Hildebrandt, K. *Heterocycles* **1986**, *24*, 297-302.

[7] Rodrigo, R.; Knabe, S. M.; Taylor, N. J.; Rajapaksa, D.; Chernishenko, M. J. *J. Org. Chem.* **1986**, *51*, 3973-3978.

[8] Sanchez-Larios, E.; Holmes, J. M.; Daschner, C. L.; Gravel, M. *Synthesis* **2011**, 1896-1904.

[9] Alajarin, M.; Bonillo, B.; Marin-Luna, M; Sanchez-Andrada, P.; Vidal, A. *Chem. Eur. J.* **2013**, *19*, 16093-16103.

$^{1}$H NMR **5a**

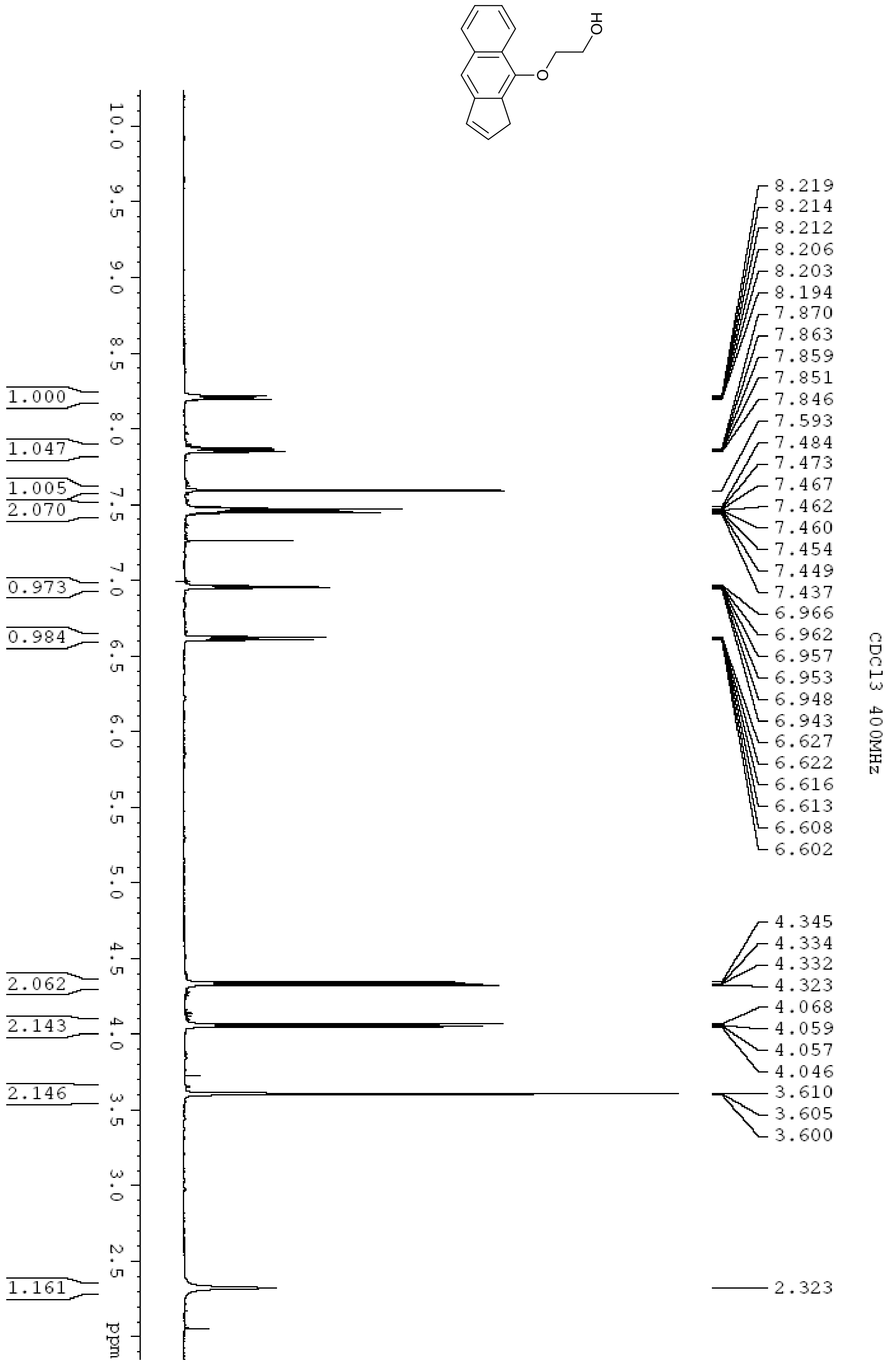

$^{13}$C NMR **5a**

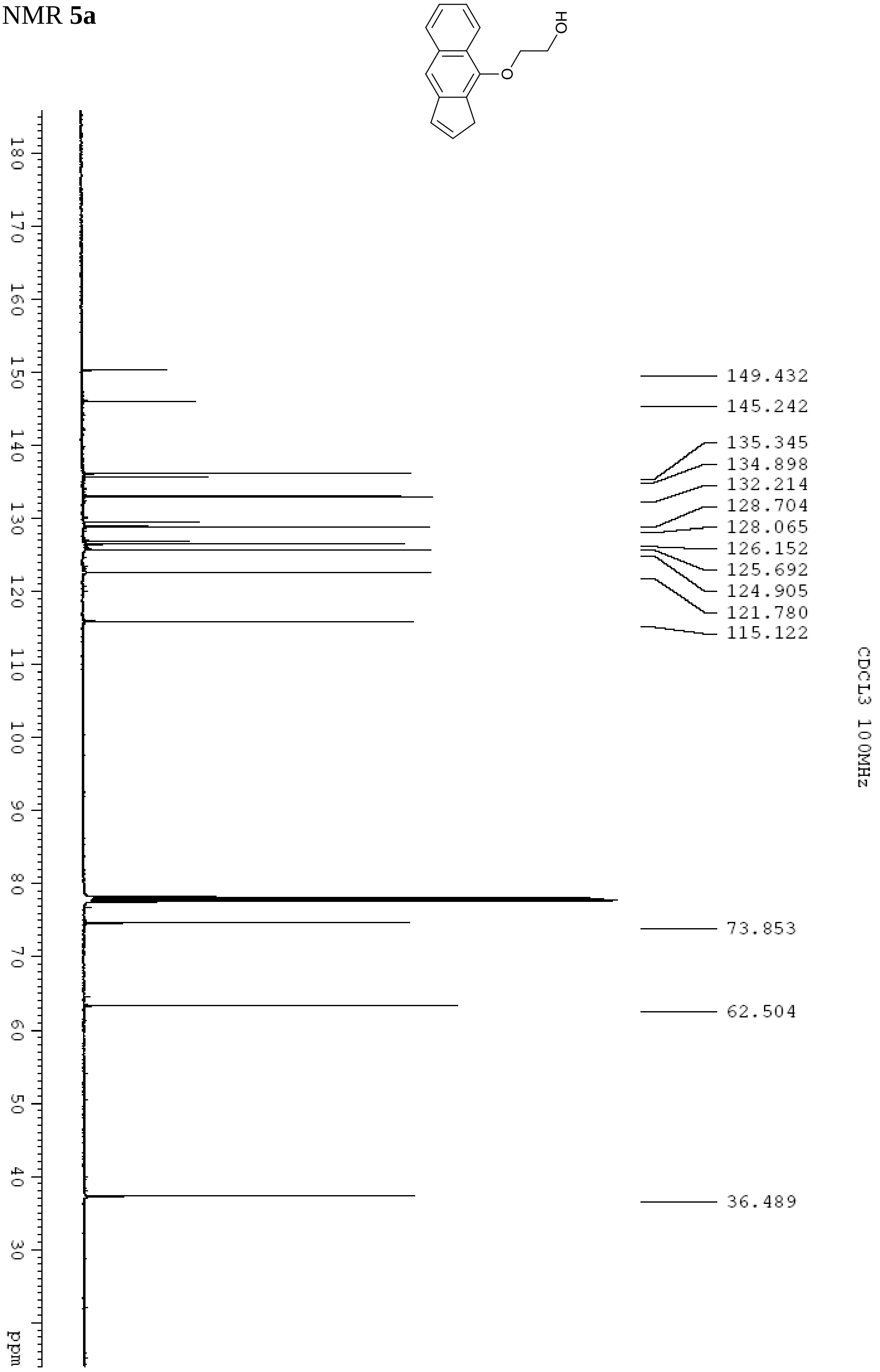

149.432
145.242
135.345
134.898
132.214
128.704
128.065
126.152
125.692
124.905
121.780
115.122
73.853
62.504
36.489
CDCL3 100MHz
ppm

$^{1}H$ NMR **6a**

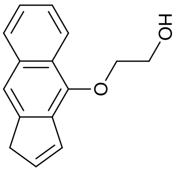

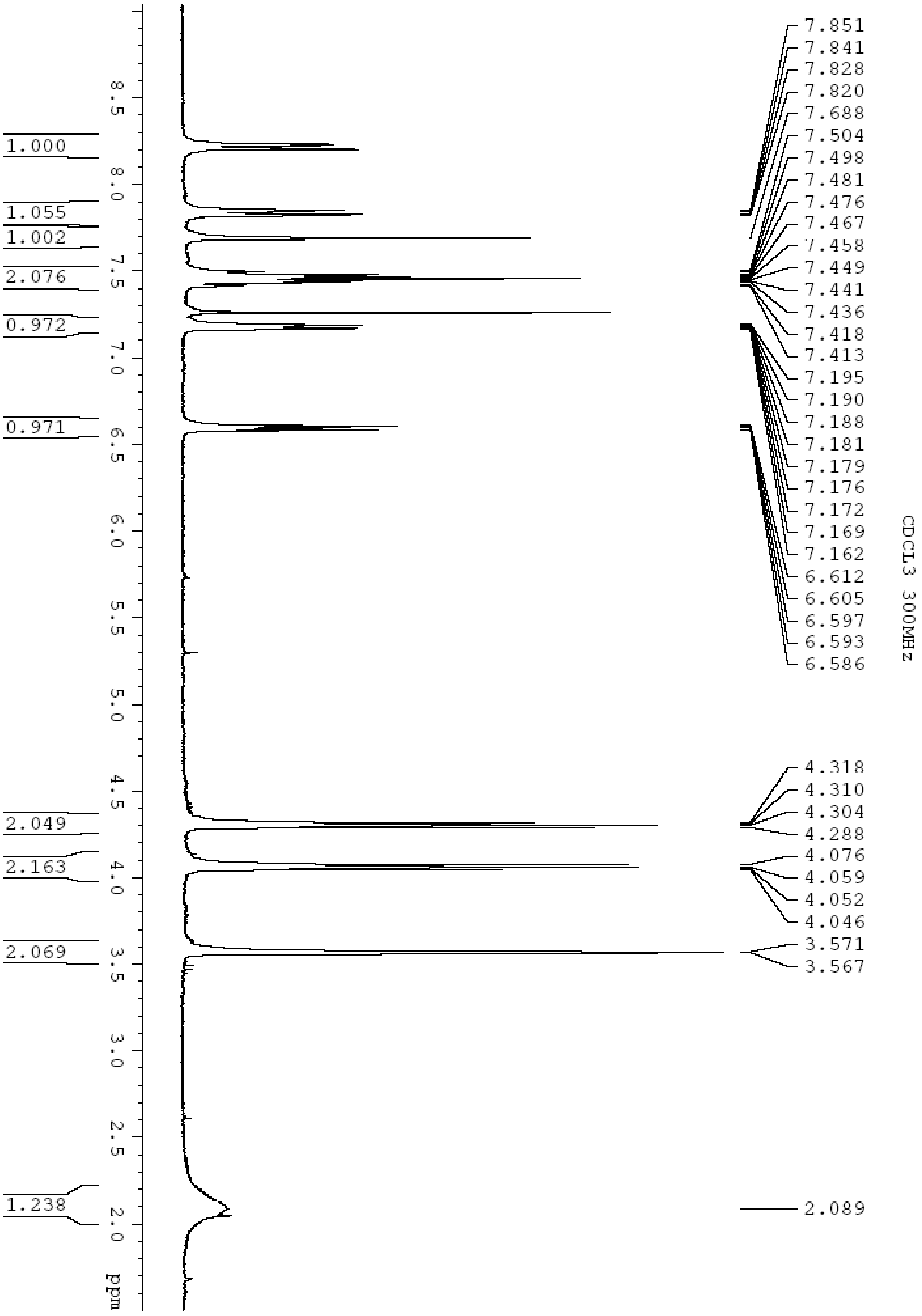

CDCL3 300MHz
7.851
7.841
7.828
7.820
7.688
7.504
7.498
7.481
7.476
7.467
7.458
7.449
7.441
7.436
7.418
7.413
7.195
7.190
7.188
7.181
7.179
7.176
7.172
7.169
7.162
6.612
6.605
6.597
6.593
6.586
4.318
4.310
4.304
4.288
4.076
4.059
4.052
4.046
3.571
3.567
2.089
1.000
1.055
1.002
2.076
0.972
0.971
2.049
2.163
2.069
1.238
8.5
8.0
7.5
7.0
6.5
6.0
5.5
5.0
4.5
4.0
3.5
3.0
2.5
2.0
ppm

$^{13}$C NMR **6a**

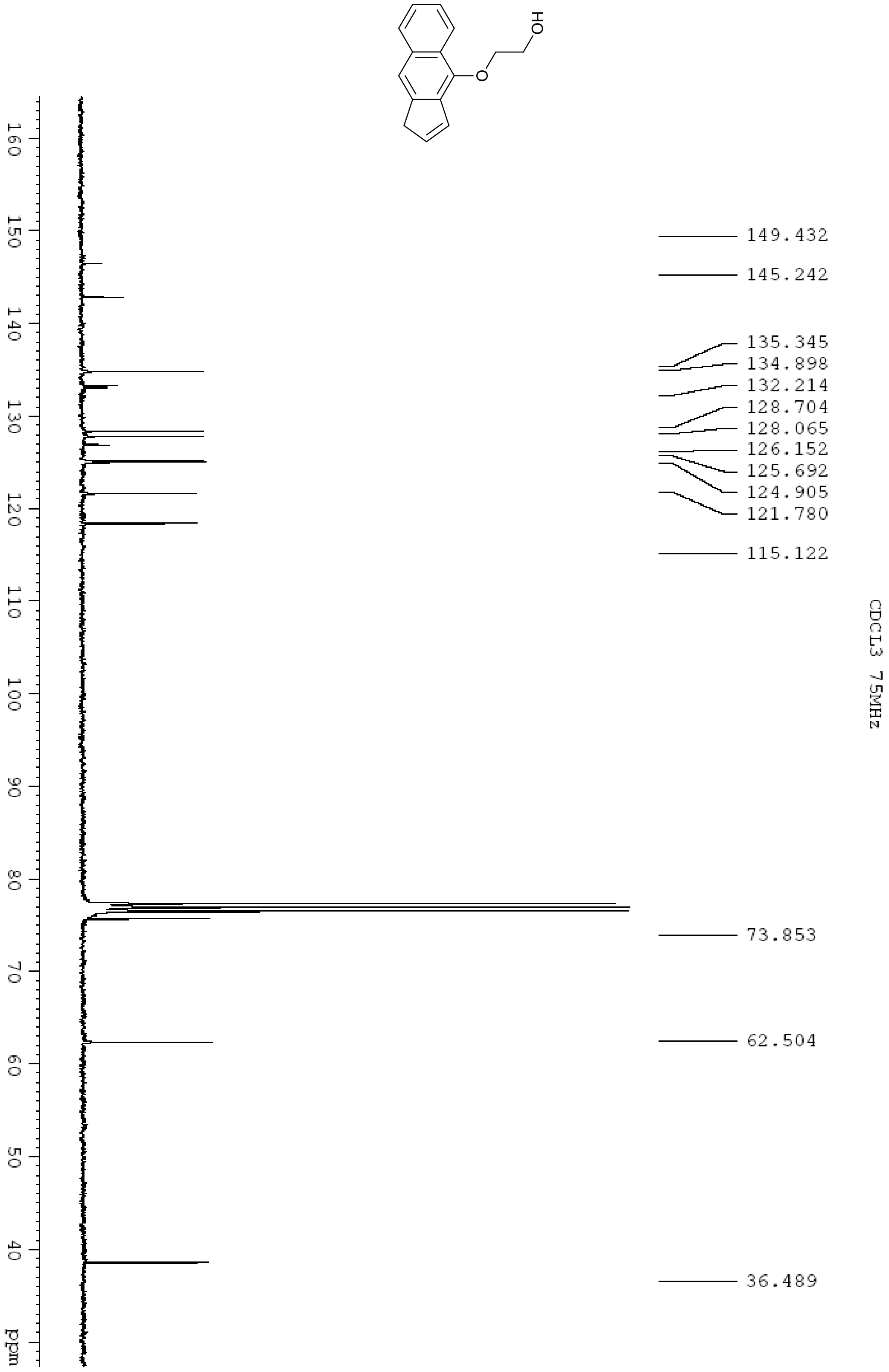

OH
O
149.432
145.242
135.345
134.898
132.214
128.704
128.065
126.152
125.692
124.905
121.780
115.122
73.853
62.504
36.489
CDCL3 75MHz
ppm

$^{1}$H NMR **5b**

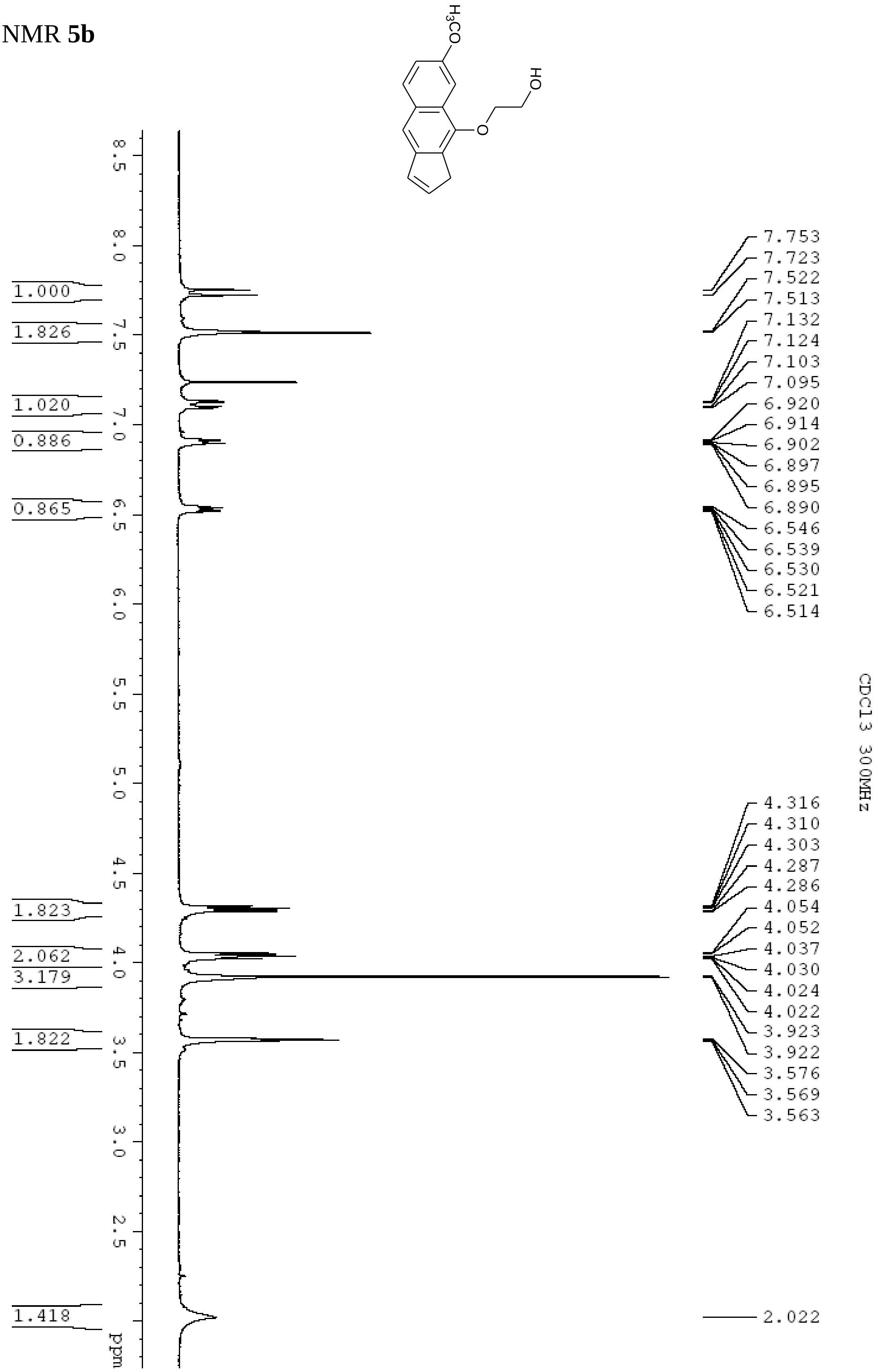
H3CO
HO
O
7.753
7.723
7.522
7.513
7.132
7.124
7.103
7.095
6.920
6.914
6.902
6.897
6.895
6.890
6.546
6.539
6.530
6.521
6.514
CDCl3 300MHz
4.316
4.310
4.303
4.287
4.286
4.054
4.052
4.037
4.030
4.024
4.022
3.923
3.922
3.576
3.569
3.563
2.022
1.000
1.826
1.020
0.886
0.865
1.823
2.062
3.179
1.822
1.418
ppm

$^{13}$C NMR **5b**

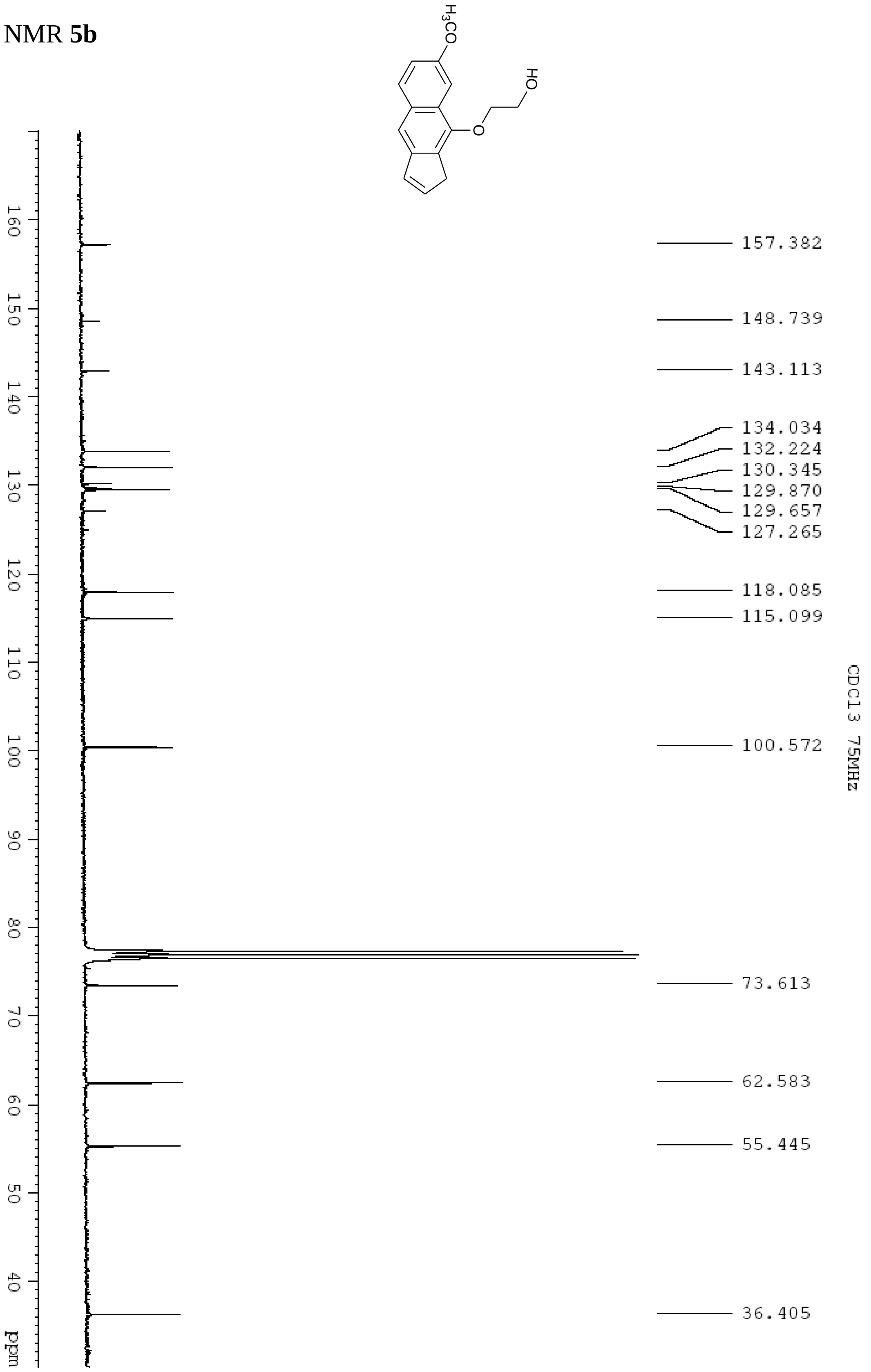
H3CO
HO
O
157.382
148.739
143.113
134.034
132.224
130.345
129.870
129.657
127.265
118.085
115.099
100.572
73.613
62.583
55.445
36.405
CDCl3 75MHz
160
150
140
130
120
110
100
90
80
70
60
50
40
ppm

$^{1}$H NMR **6b**

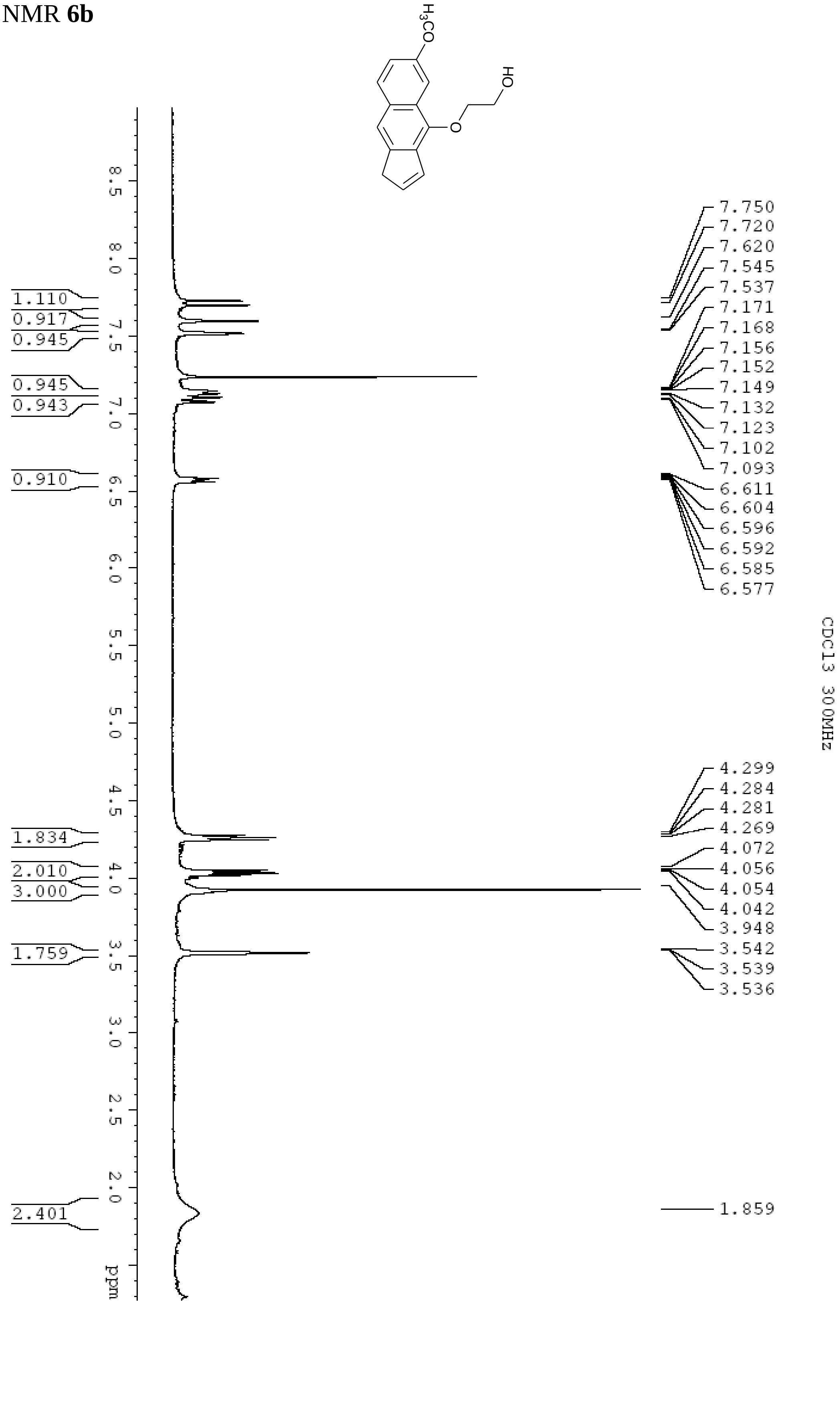

H3CO
HO
O
7.750
7.720
7.620
7.545
7.537
7.171
7.168
7.156
7.152
7.149
7.132
7.123
7.102
7.093
6.611
6.604
6.596
6.592
6.585
6.577
CDCl3 300MHz
4.299
4.284
4.281
4.269
4.072
4.056
4.054
4.042
3.948
3.542
3.539
3.536
1.859
1.110
0.917
0.945
0.945
0.943
0.910
1.834
2.010
3.000
1.759
2.401
ppm

$^{13}$C NMR **6b**

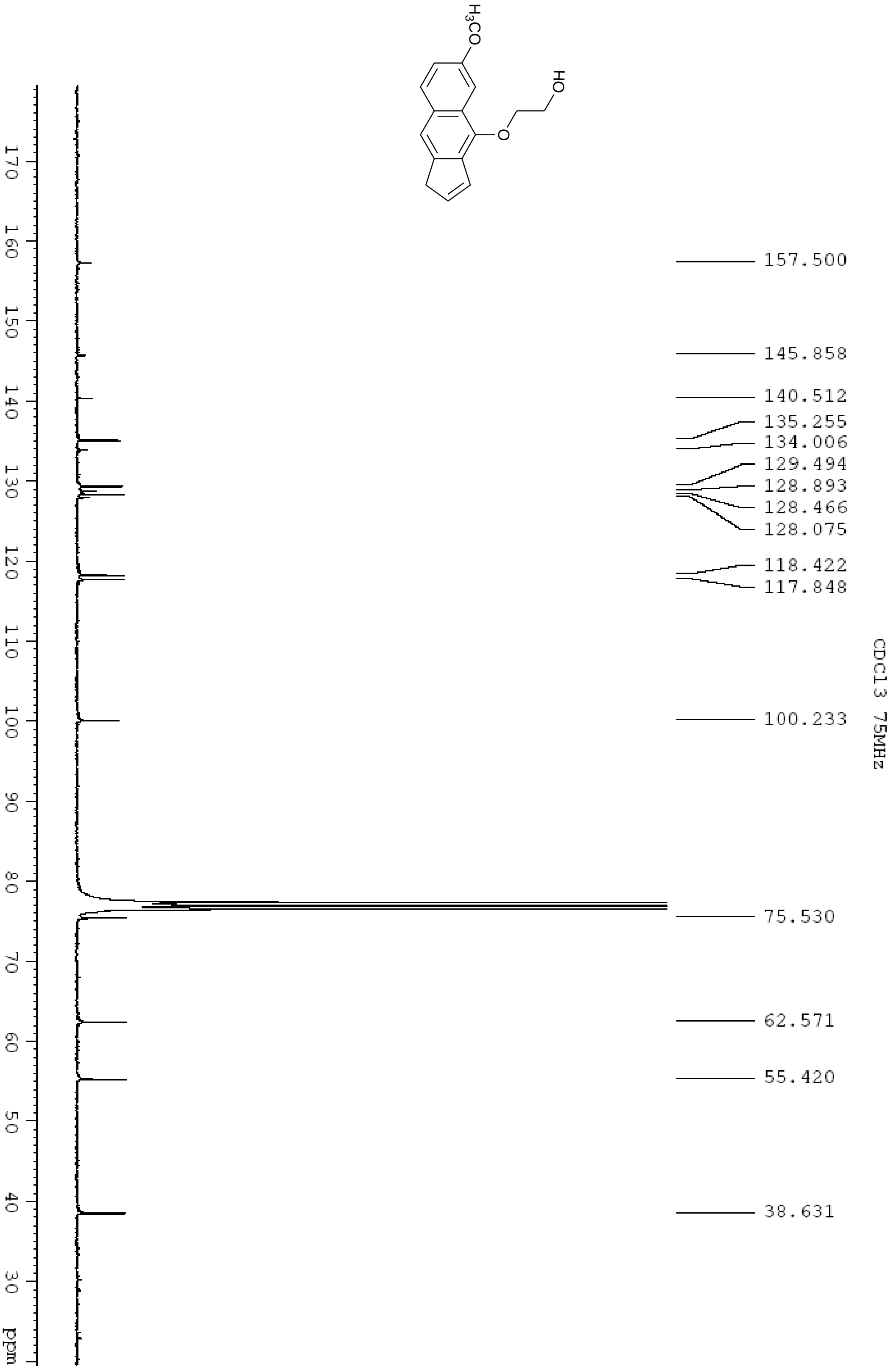
157.500
145.858
140.512
135.255
134.006
129.494
128.893
128.466
128.075
118.422
117.848
100.233
75.530
62.571
55.420
38.631
CDCl3 75MHz
ppm

$^{1}$H NMR **5c**

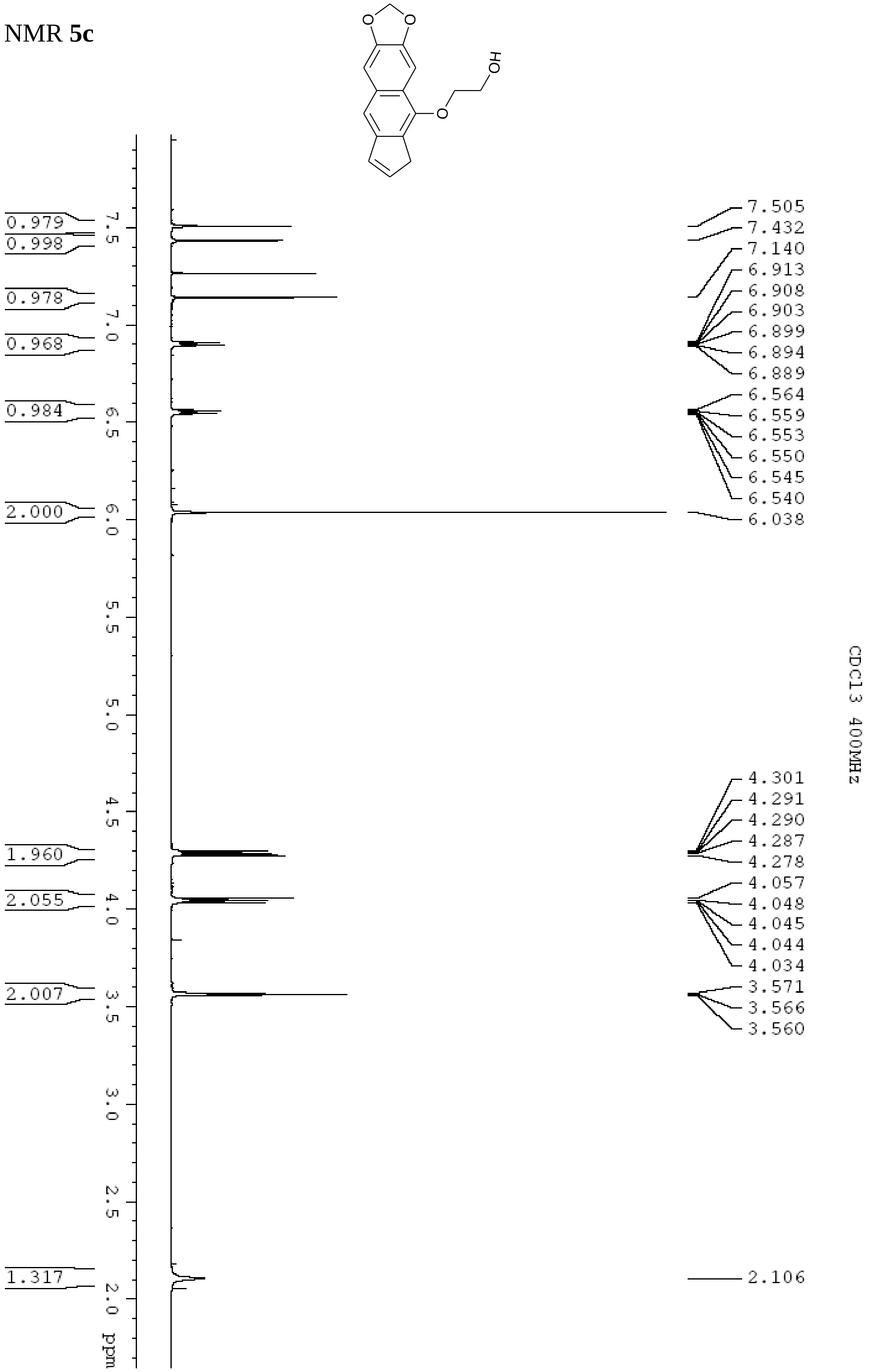
OH
O
O
O
7.505
7.432
7.140
6.913
6.908
6.903
6.899
6.894
6.889
6.564
6.559
6.553
6.550
6.545
6.540
6.038
4.301
4.291
4.290
4.287
4.278
4.057
4.048
4.045
4.044
4.034
3.571
3.566
3.560
2.106
0.979
0.998
0.978
0.968
0.984
2.000
1.960
2.055
2.007
1.317
7.5
7.0
6.5
6.0
5.5
5.0
4.5
4.0
3.5
3.0
2.5
2.0 ppm
CDCl3 400MHz

$^{13}C$ NMR **5c**

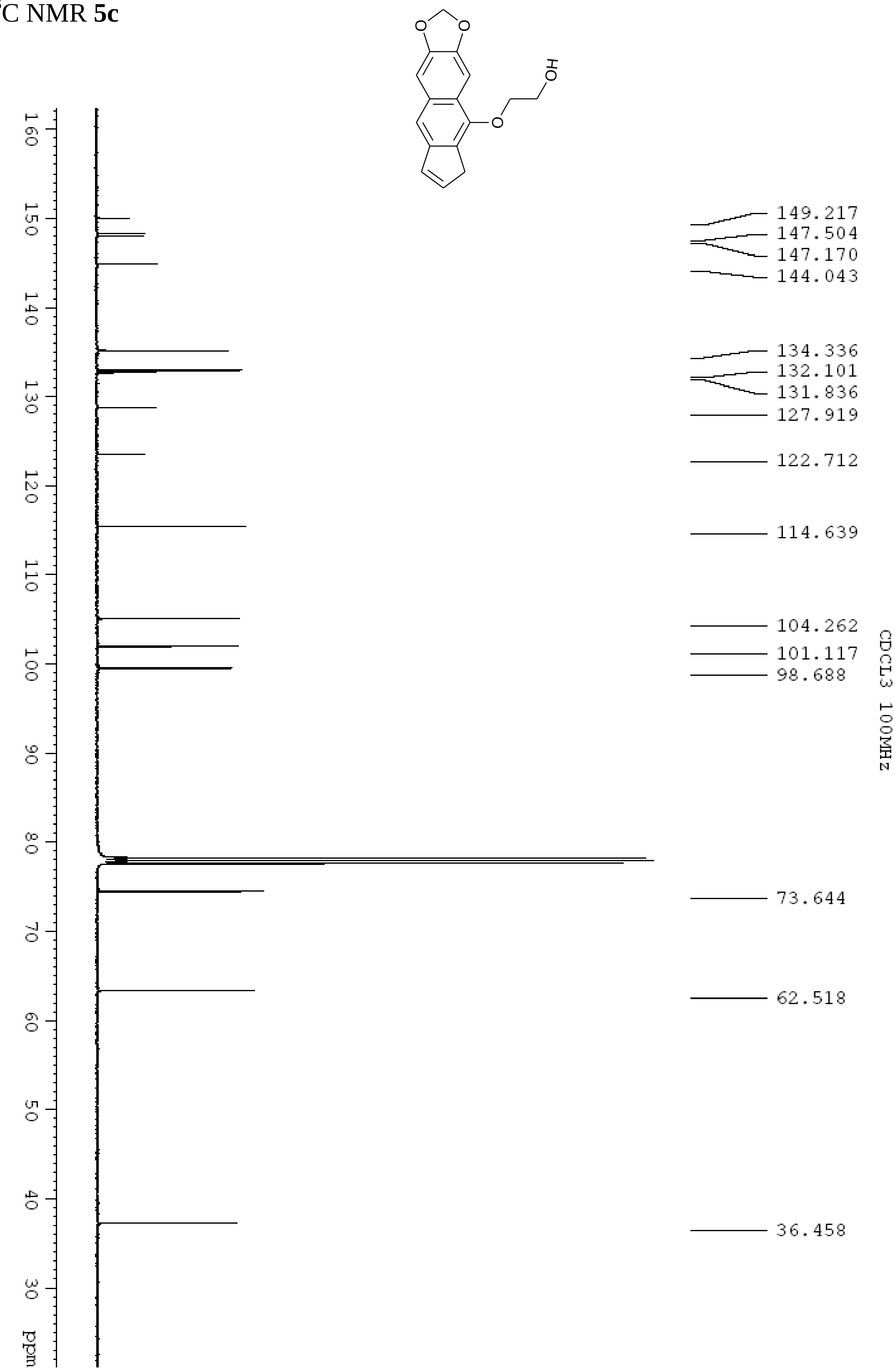
HO
O
149.217
147.504
147.170
144.043
134.336
132.101
131.836
127.919
122.712
114.639
104.262
101.117
98.688
73.644
62.518
36.458
CDCL3 100MHz
160
150
140
130
120
110
100
90
80
70
60
50
40
30
ppm

$^{1}$H NMR **6c**

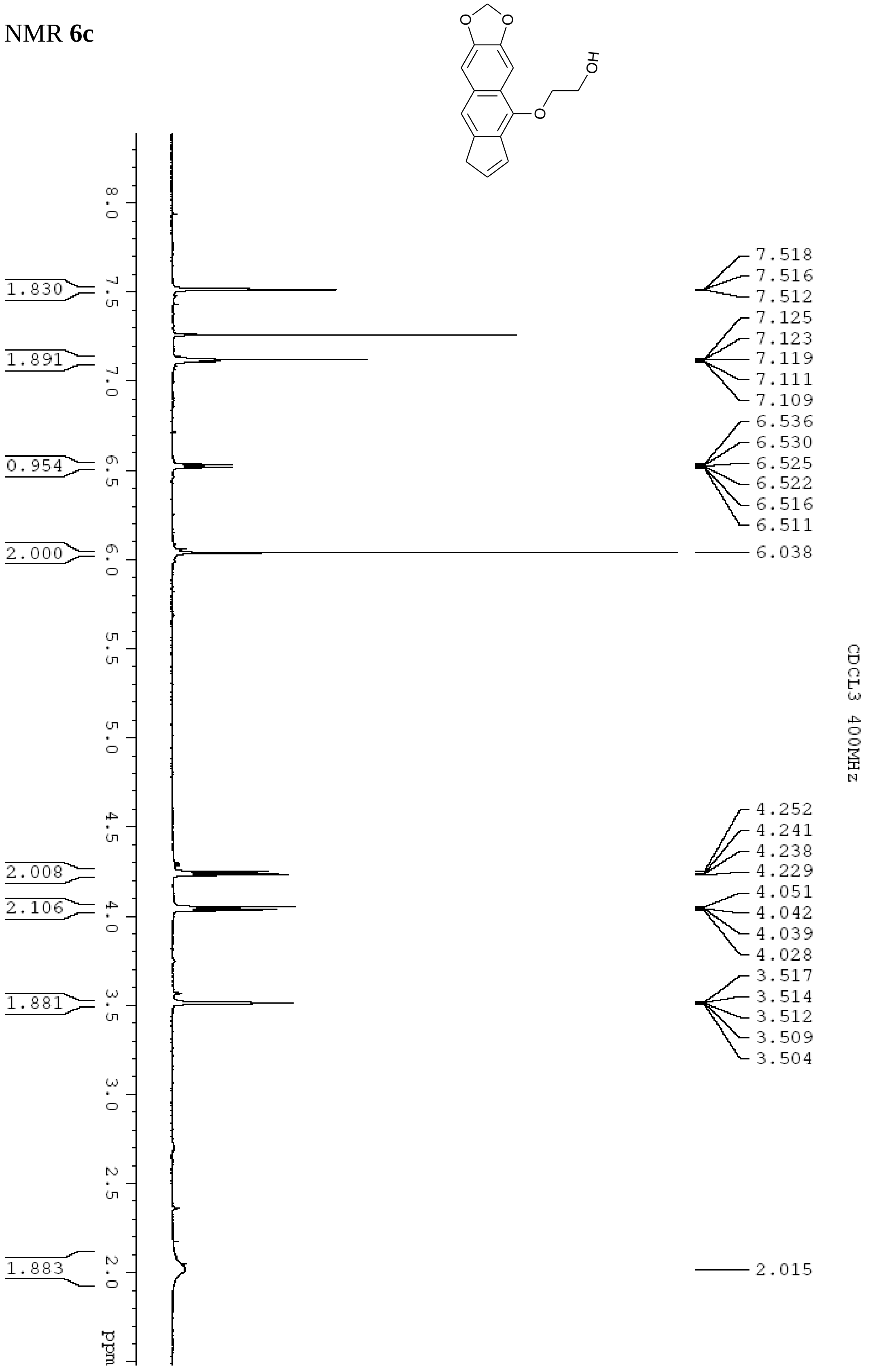
OH
O
7.518
7.516
7.512
7.125
7.123
7.119
7.111
7.109
6.536
6.530
6.525
6.522
6.516
6.511
6.038
CDCL3 400MHz
4.252
4.241
4.238
4.229
4.051
4.042
4.039
4.028
3.517
3.514
3.512
3.509
3.504
2.015
1.830
1.891
0.954
2.000
2.008
2.106
1.881
1.883
ppm

$^{13}C$ NMR **6c**

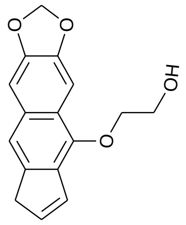

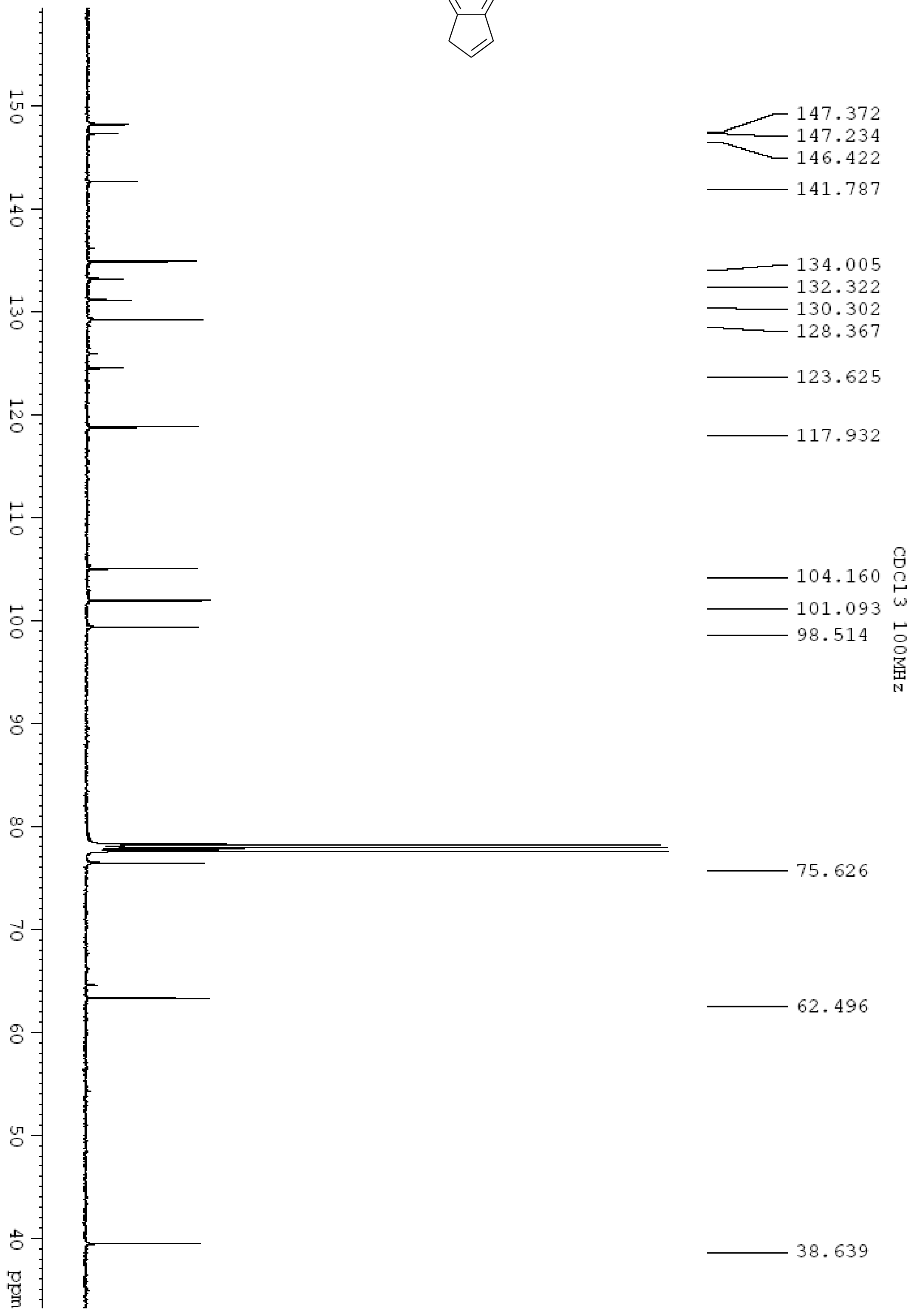
147.372
147.234
146.422
141.787
134.005
132.322
130.302
128.367
123.625
117.932
104.160
101.093
98.514
75.626
62.496
38.639
CDCl3 100MHz
150
140
130
120
110
100
90
80
70
60
50
40
ppm

$^{1}$H NMR **5d**

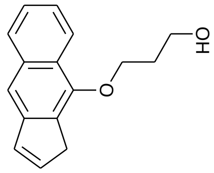
O
OH

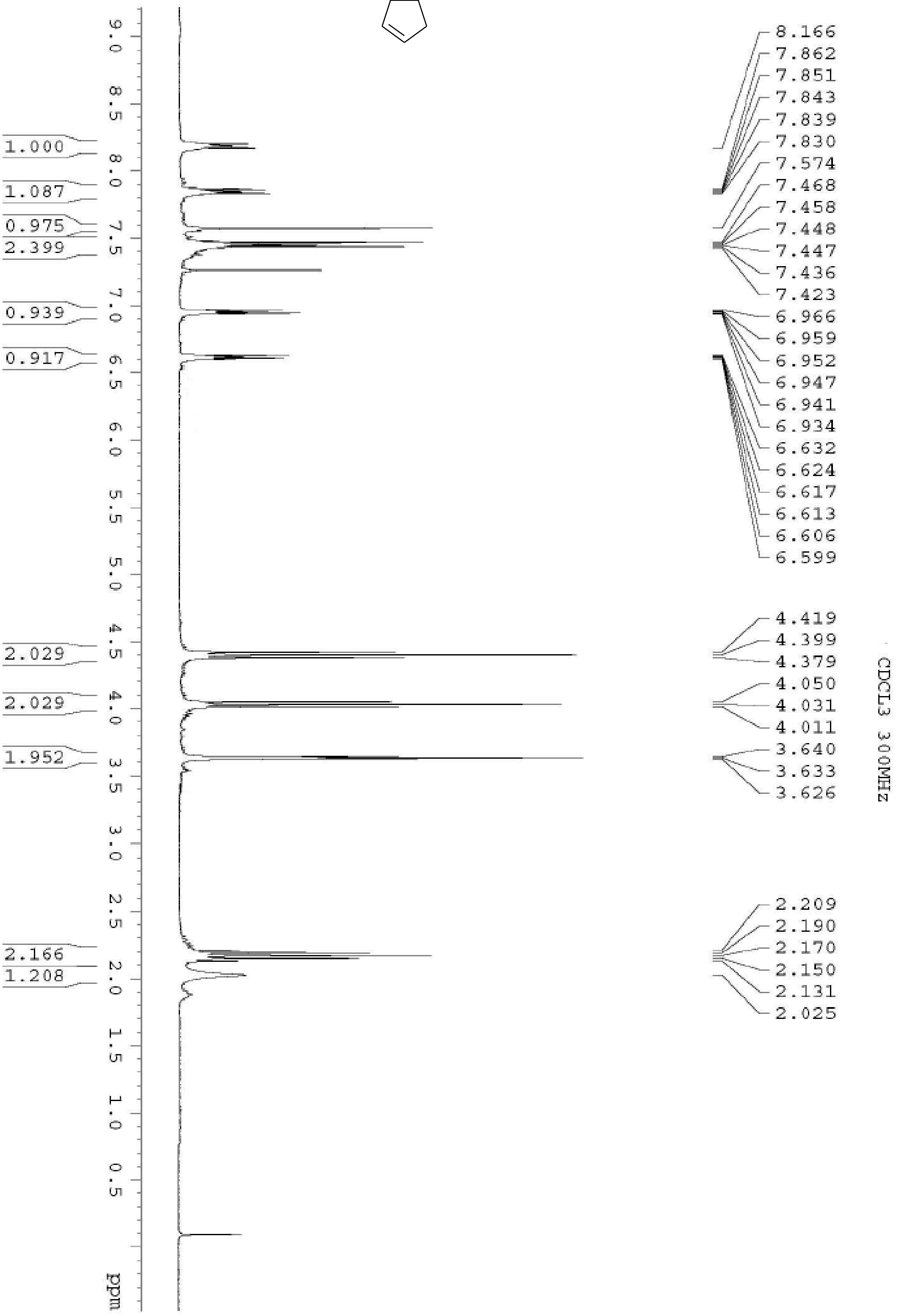
1.000
1.087
0.975
2.399
0.939
0.917
2.029
2.029
1.952
2.166
1.208
8.166
7.862
7.851
7.843
7.839
7.830
7.574
7.468
7.458
7.448
7.447
7.436
7.423
6.966
6.959
6.952
6.947
6.941
6.934
6.632
6.624
6.617
6.613
6.606
6.599
4.419
4.399
4.379
4.050
4.031
4.011
3.640
3.633
3.626
2.209
2.190
2.170
2.150
2.131
2.025
CDCL3 300MHz
ppm

$^{13}$C NMR **5d**

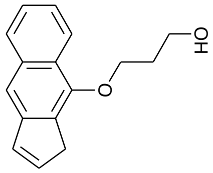
OH
O

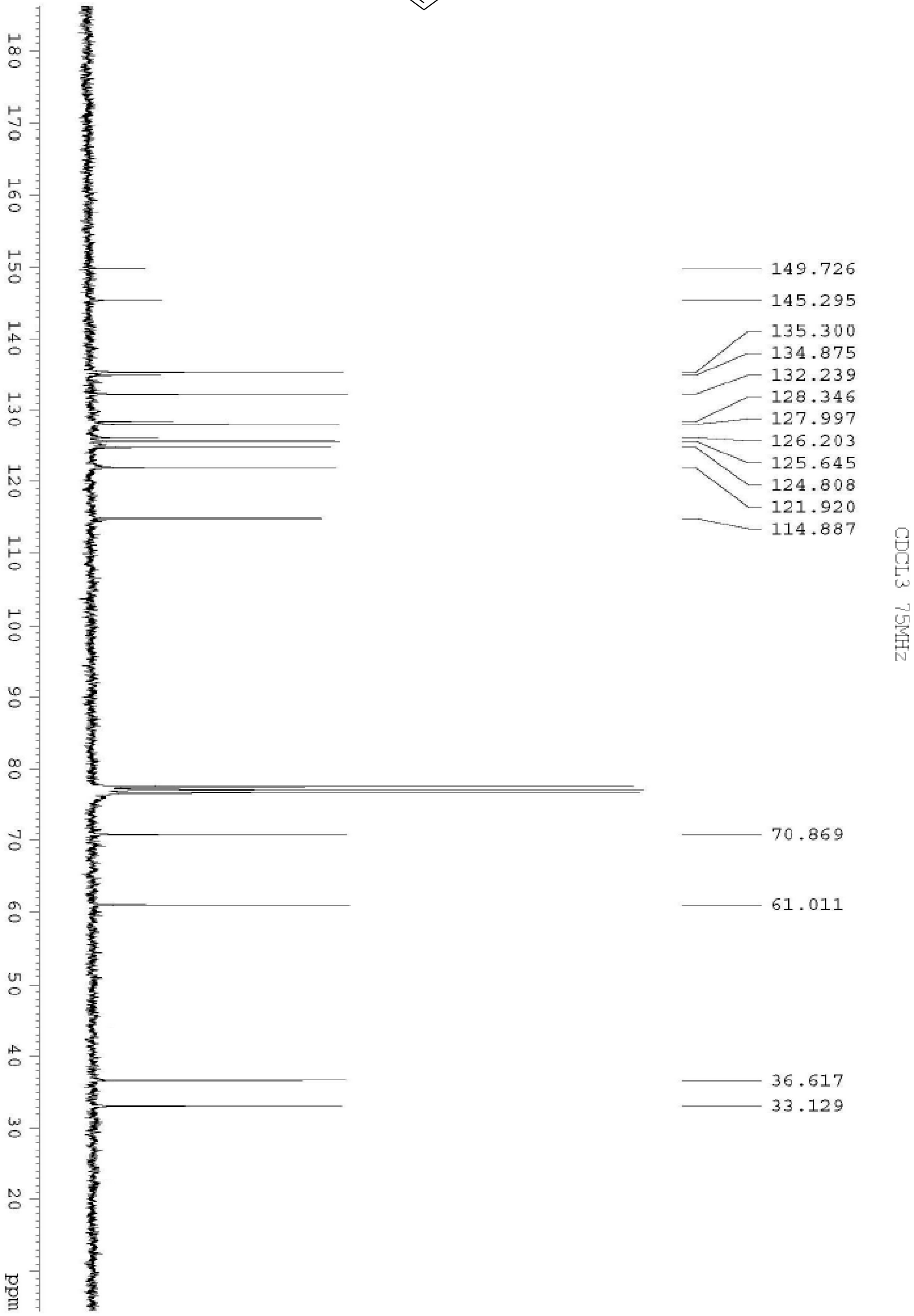
149.726
145.295
135.300
134.875
132.239
128.346
127.997
126.203
125.645
124.808
121.920
114.887
70.869
61.011
36.617
33.129
CDCL3 75MHz
ppm

$^{1}$H NMR **6d**

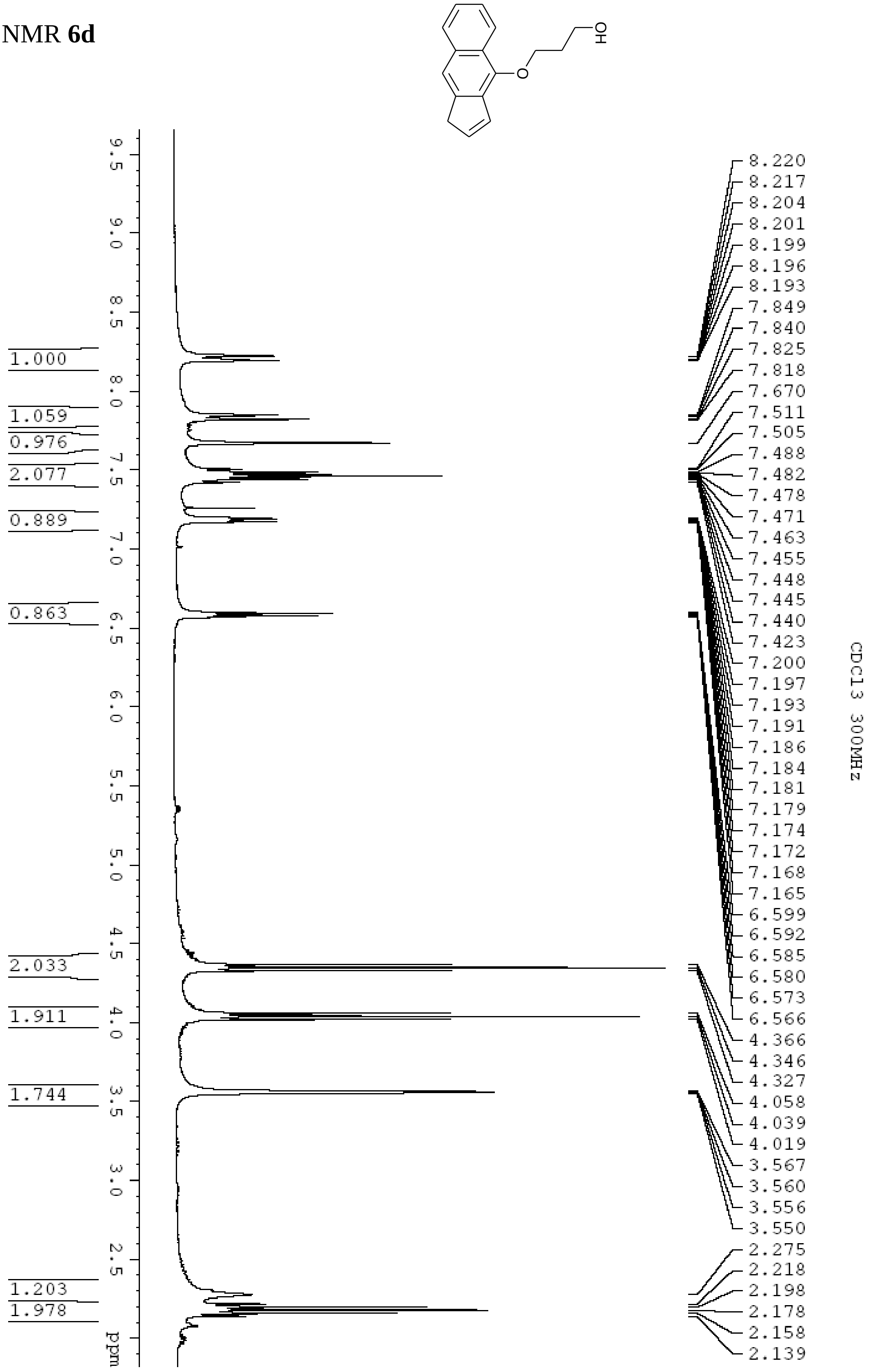
OH
O
CDCl3 300MHz
8.220
8.217
8.204
8.201
8.199
8.196
8.193
7.849
7.840
7.825
7.818
7.670
7.511
7.505
7.488
7.482
7.478
7.471
7.463
7.455
7.448
7.445
7.440
7.423
7.200
7.197
7.193
7.191
7.186
7.184
7.181
7.179
7.174
7.172
7.168
7.165
6.599
6.592
6.585
6.580
6.573
6.566
4.366
4.346
4.327
4.058
4.039
4.019
3.567
3.560
3.556
3.550
2.275
2.218
2.198
2.178
2.158
2.139
1.000
1.059
0.976
2.077
0.889
0.863
2.033
1.911
1.744
1.203
1.978
9.5
9.0
8.5
8.0
7.5
7.0
6.5
6.0
5.5
5.0
4.5
4.0
3.5
3.0
2.5
ppm

$^{13}$C NMR **6d**

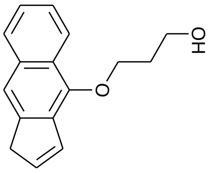
O
OH

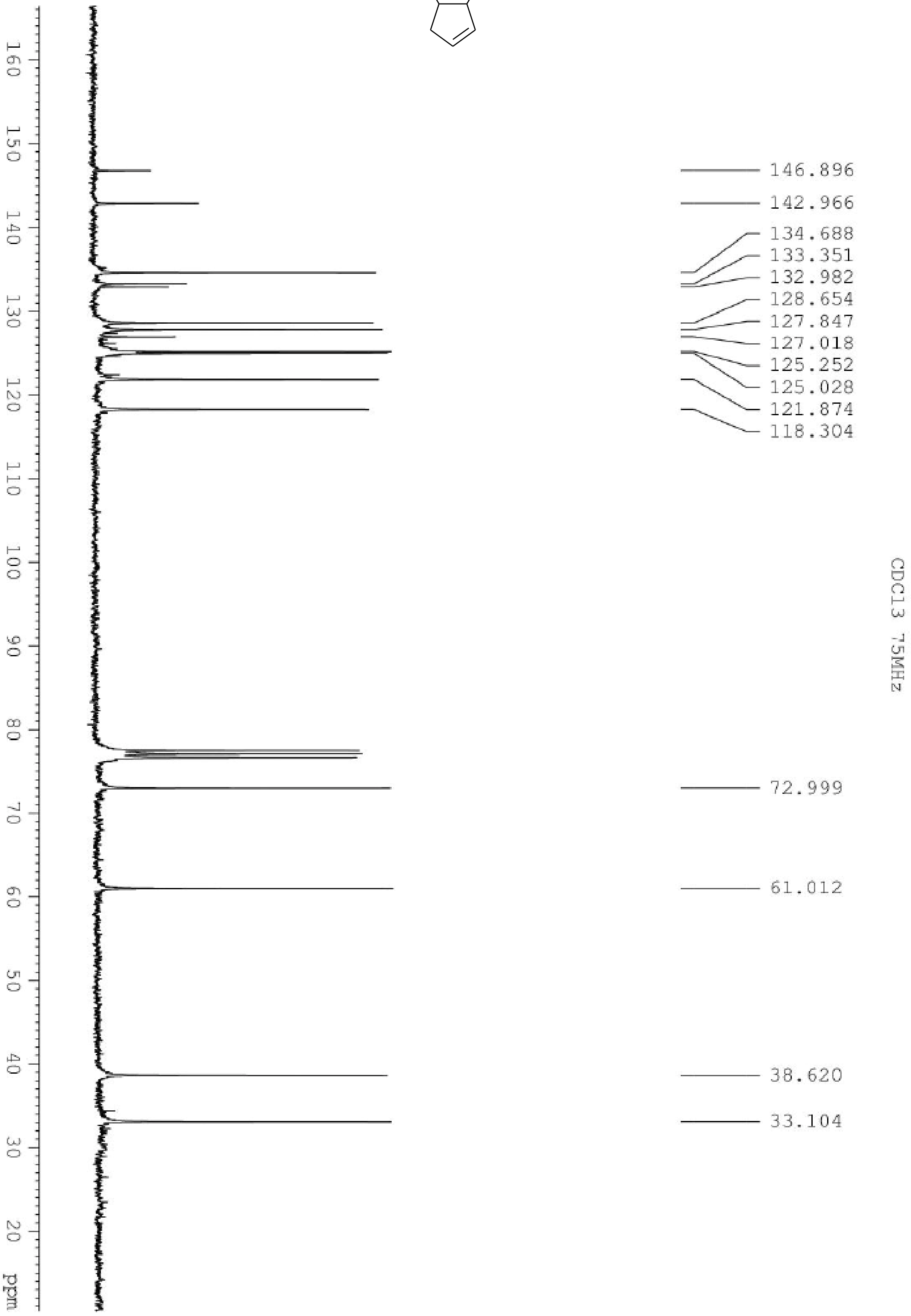
146.896
142.966
134.688
133.351
132.982
128.654
127.847
127.018
125.252
125.028
121.874
118.304
72.999
61.012
38.620
33.104
CDCl3 75MHz
ppm

$^{1}$H NMR **5e** + **6e**

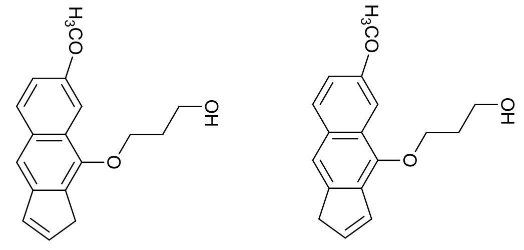
H3CO
OH
O
H3CO
OH
O

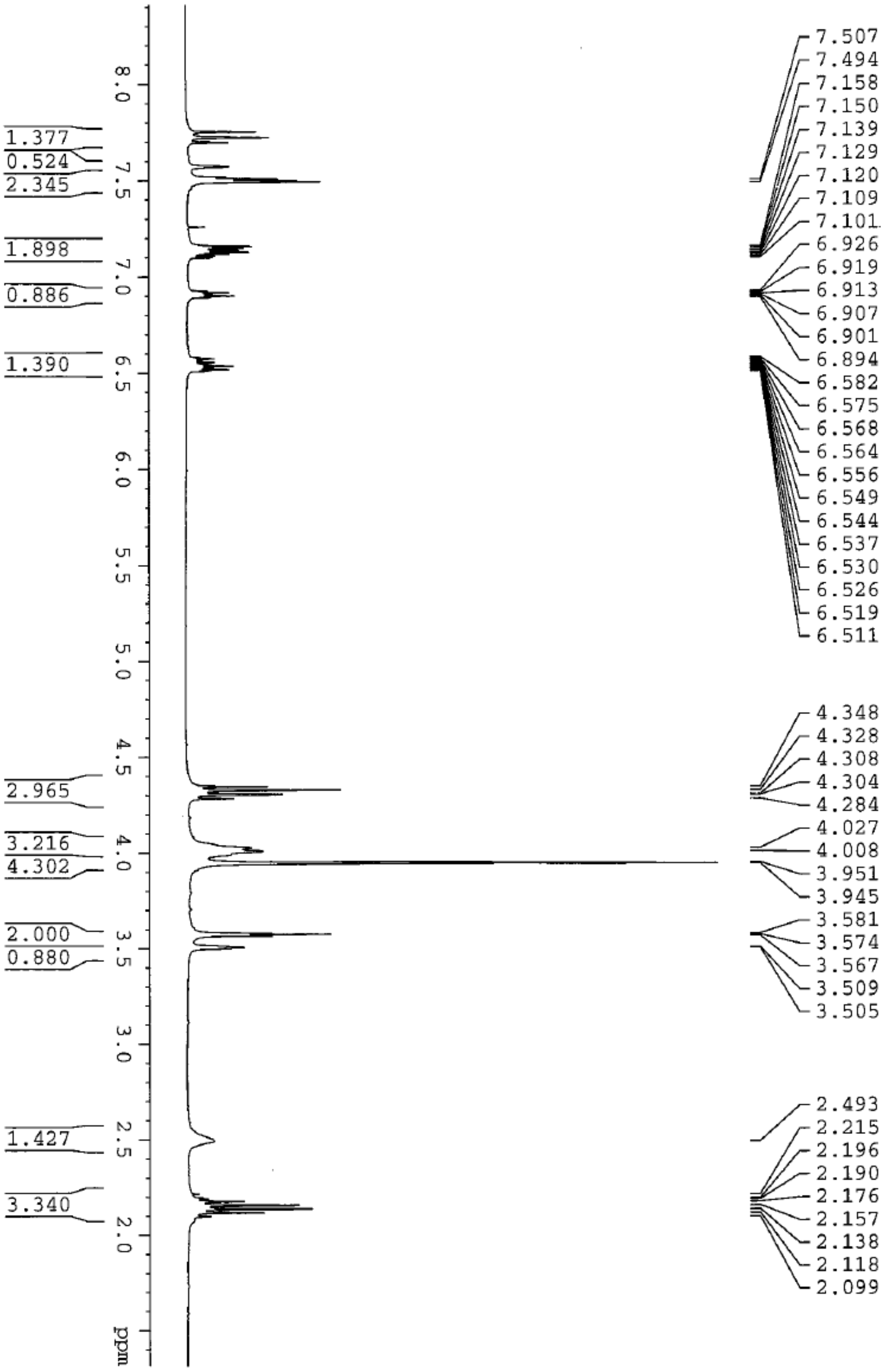
1.377
0.524
2.345
1.898
0.886
1.390
2.965
3.216
4.302
2.000
0.880
1.427
3.340
8.0
7.5
7.0
6.5
6.0
5.5
5.0
4.5
4.0
3.5
3.0
2.5
2.0
ppm
7.507
7.494
7.158
7.150
7.139
7.129
7.120
7.109
7.101
6.926
6.919
6.913
6.907
6.901
6.894
6.582
6.575
6.568
6.564
6.556
6.549
6.544
6.537
6.530
6.526
6.519
6.511
4.348
4.328
4.308
4.304
4.284
4.027
4.008
3.951
3.945
3.581
3.574
3.567
3.509
3.505
2.493
2.215
2.196
2.190
2.176
2.157
2.138
2.118
2.099

$^{13}$C NMR **5e** + **6e**

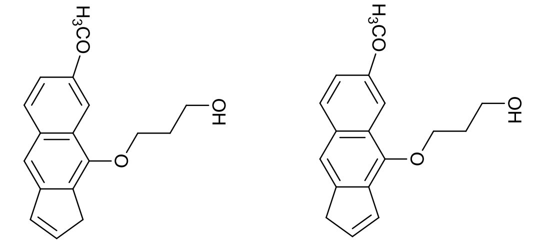
H3CO
OH
O
H3CO
OH
O

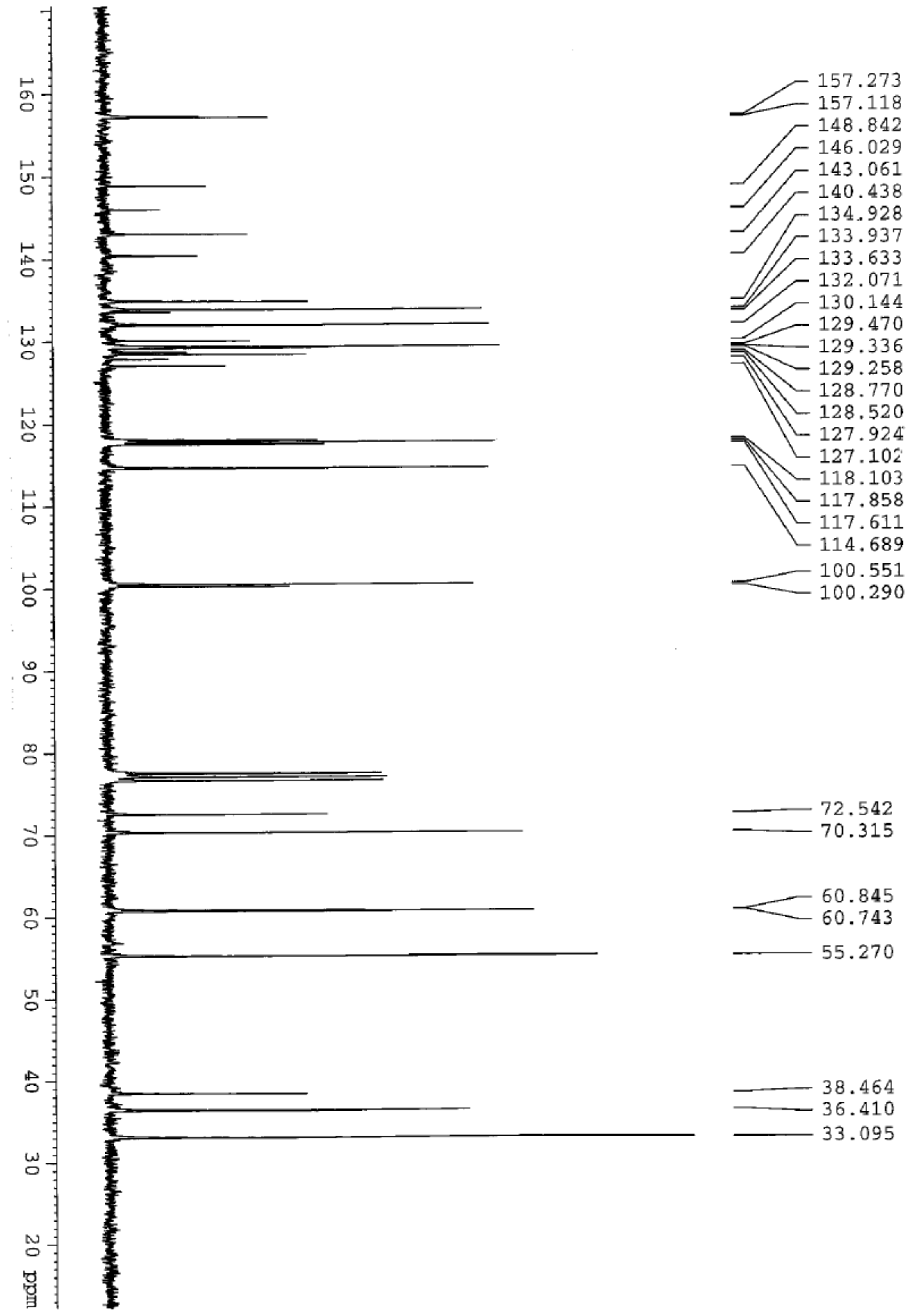
157.273
157.118
148.842
146.029
143.061
140.438
134.928
133.937
133.633
132.071
130.144
129.470
129.336
129.258
128.770
128.520
127.924
127.102
118.103
117.858
117.611
114.689
100.551
100.290
72.542
70.315
60.845
60.743
55.270
38.464
36.410
33.095
160
150
140
130
120
110
100
90
80
70
60
50
40
30
20 ppm

$^{1}$H NMR **5f**

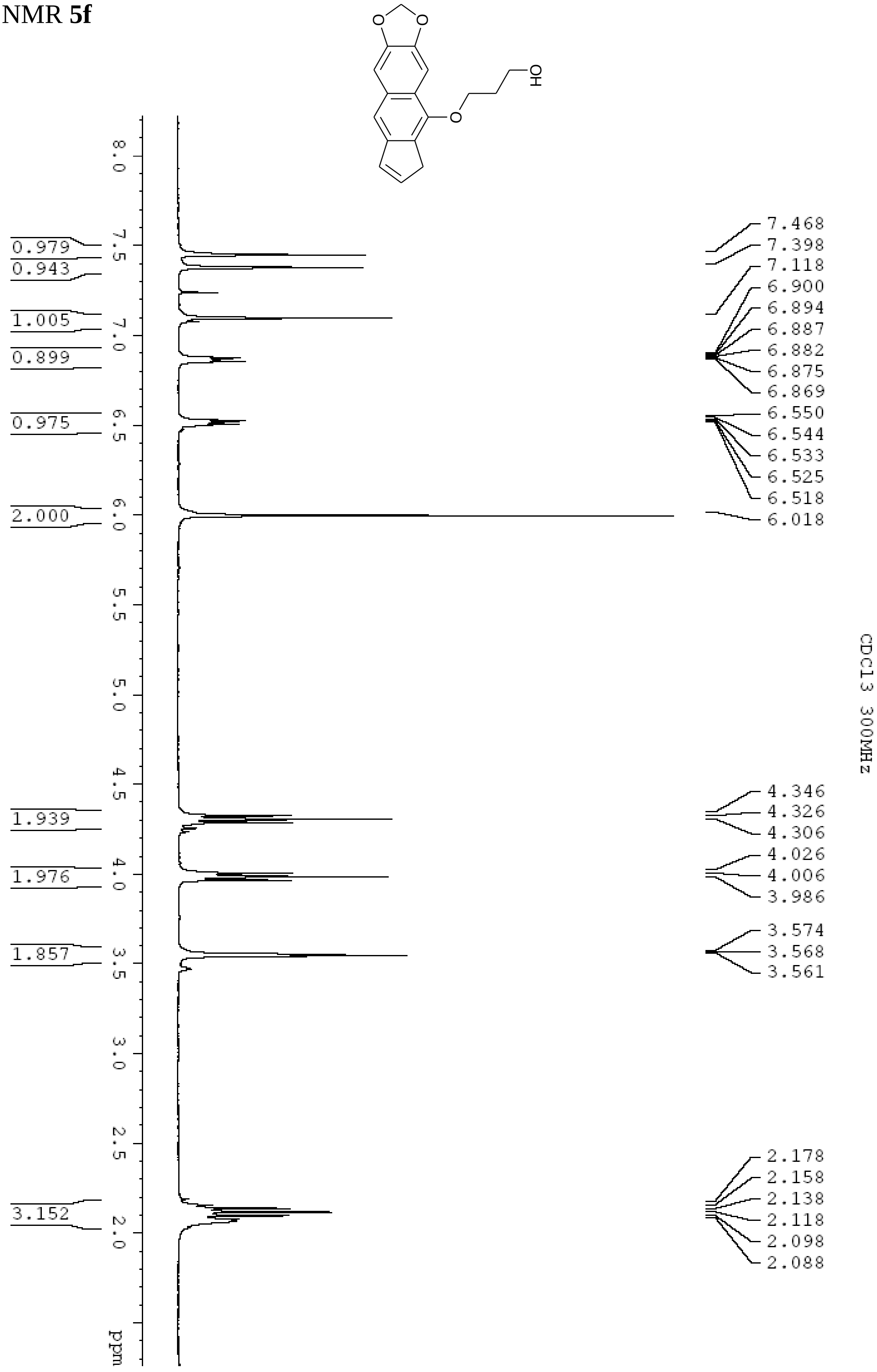

OH
O
CDCl3 300MHz
ppm
8.0
7.5
7.0
6.5
6.0
5.5
5.0
4.5
4.0
3.5
3.0
2.5
2.0
0.979
0.943
1.005
0.899
0.975
2.000
1.939
1.976
1.857
3.152
7.468
7.398
7.118
6.900
6.894
6.887
6.882
6.875
6.869
6.550
6.544
6.533
6.525
6.518
6.018
4.346
4.326
4.306
4.026
4.006
3.986
3.574
3.568
3.561
2.178
2.158
2.138
2.118
2.098
2.088

$^{13}$C NMR **5f**

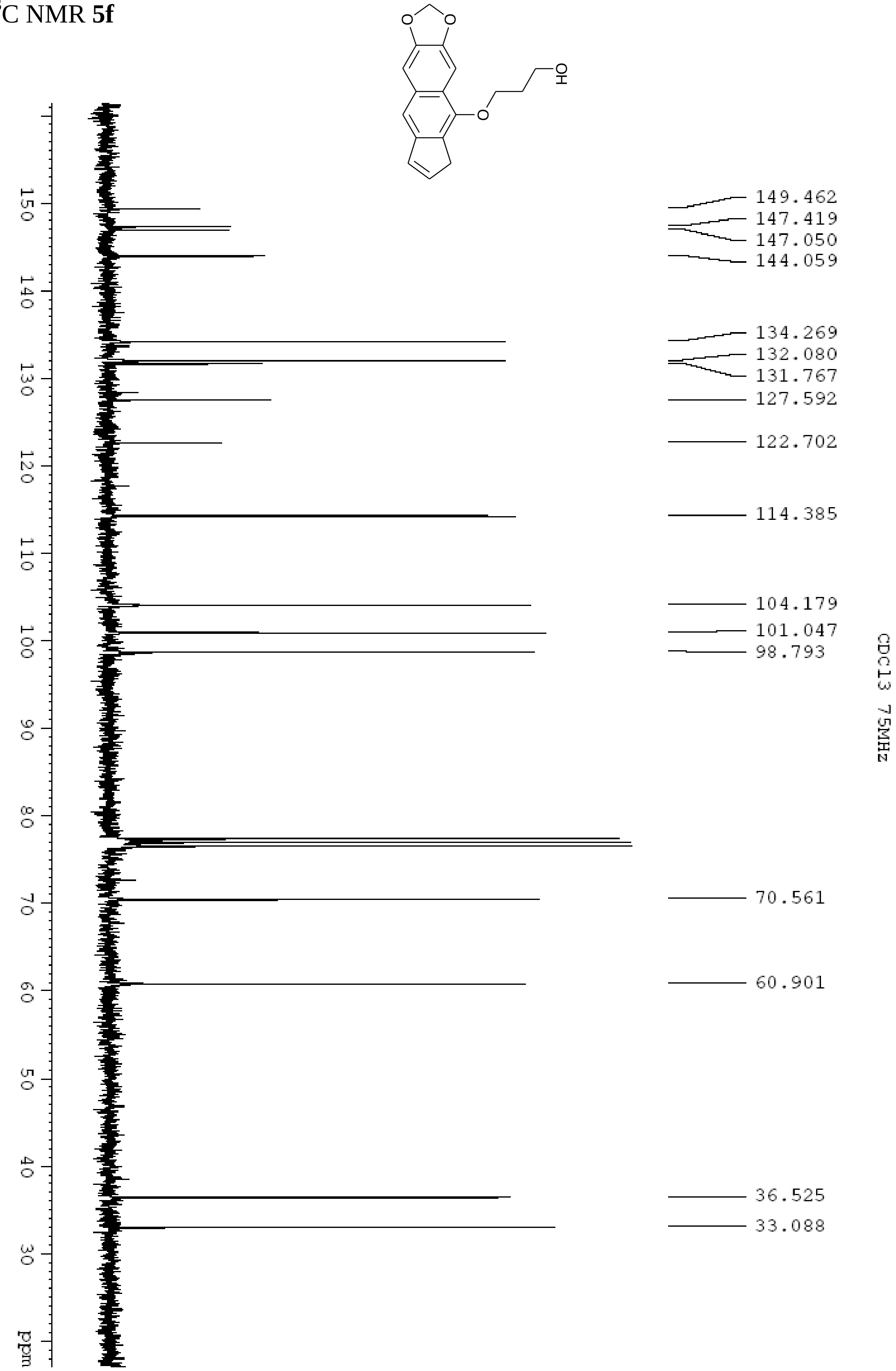

149.462
147.419
147.050
144.059
134.269
132.080
131.767
127.592
122.702
114.385
104.179
101.047
98.793
70.561
60.901
36.525
33.088
CDCl3 75MHz
ppm

$^{1}$H NMR **6f**

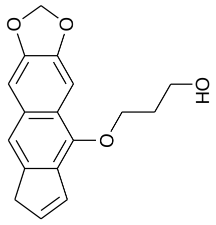
O
O
O
OH

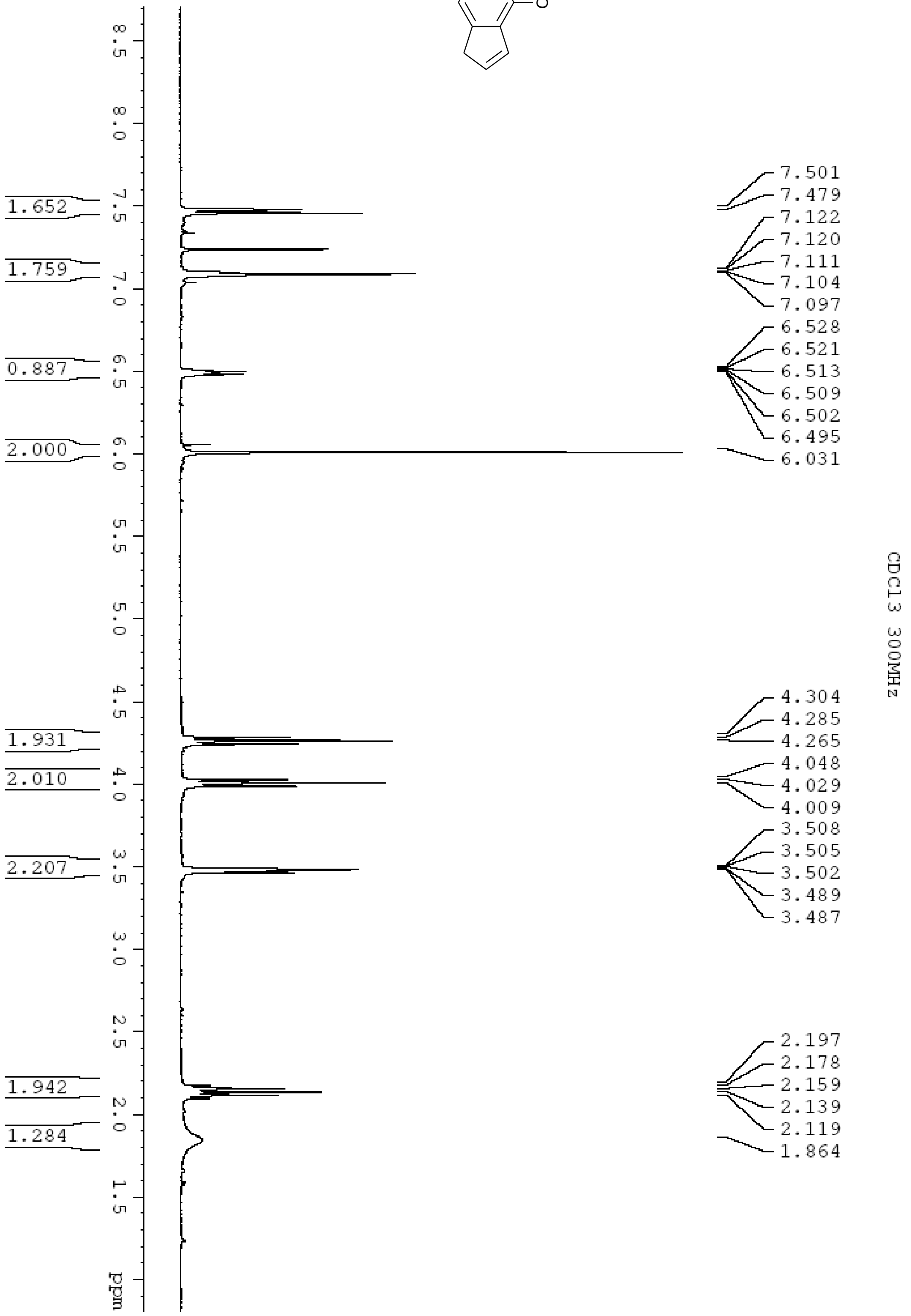
CDCl3 300MHz
ppm
8.5
8.0
7.5
7.0
6.5
6.0
5.5
5.0
4.5
4.0
3.5
3.0
2.5
2.0
1.5
1.652
1.759
0.887
2.000
1.931
2.010
2.207
1.942
1.284
7.501
7.479
7.122
7.120
7.111
7.104
7.097
6.528
6.521
6.513
6.509
6.502
6.495
6.031
4.304
4.285
4.265
4.048
4.029
4.009
3.508
3.505
3.502
3.489
3.487
2.197
2.178
2.159
2.139
2.119
1.864

$^{13}$C NMR **6f**

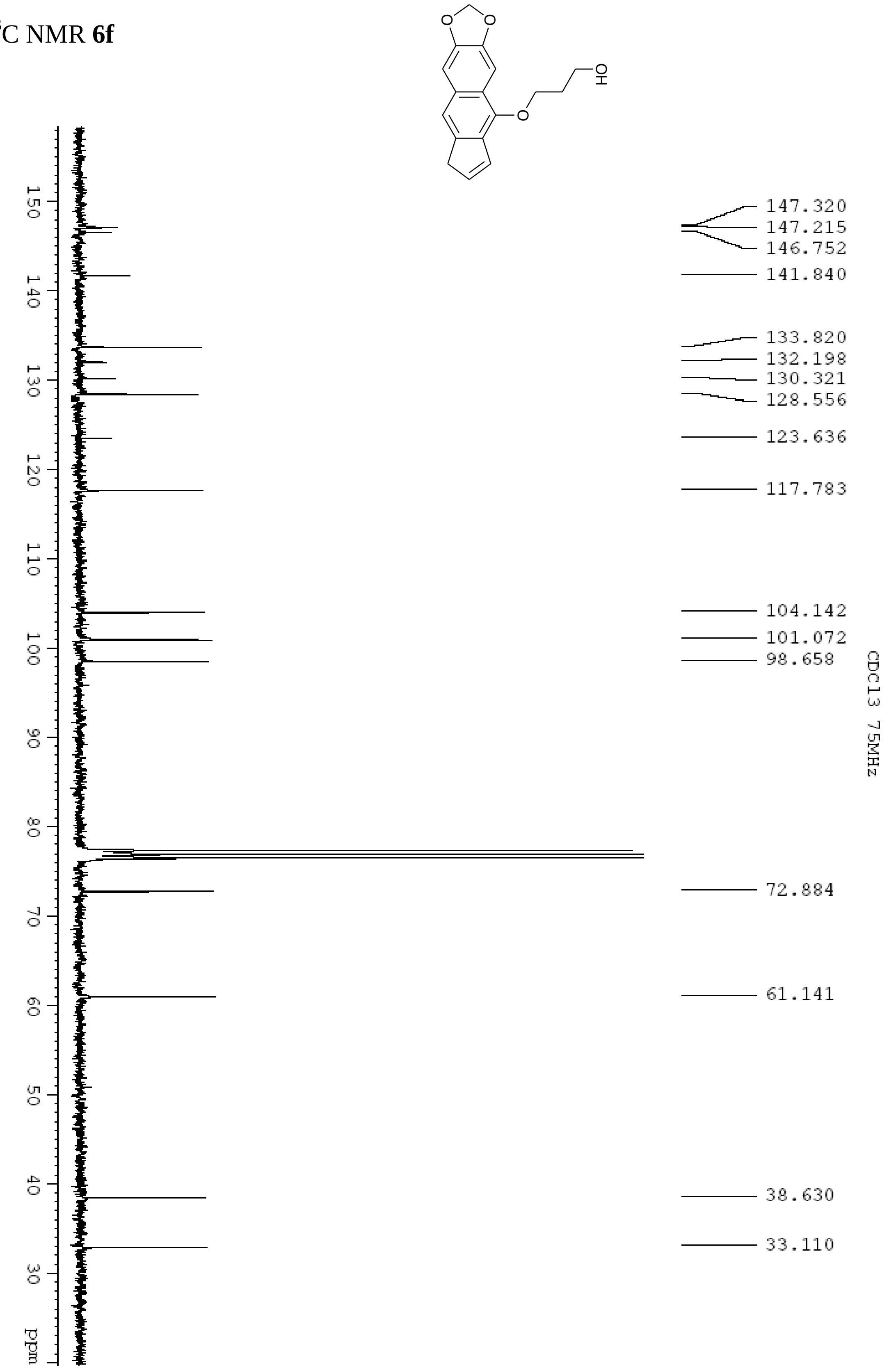
OH
O
147.320
147.215
146.752
141.840
133.820
132.198
130.321
128.556
123.636
117.783
104.142
101.072
98.658
72.884
61.141
38.630
33.110
CDCl3 75MHz
ppm

$^{1}$H NMR **25a**

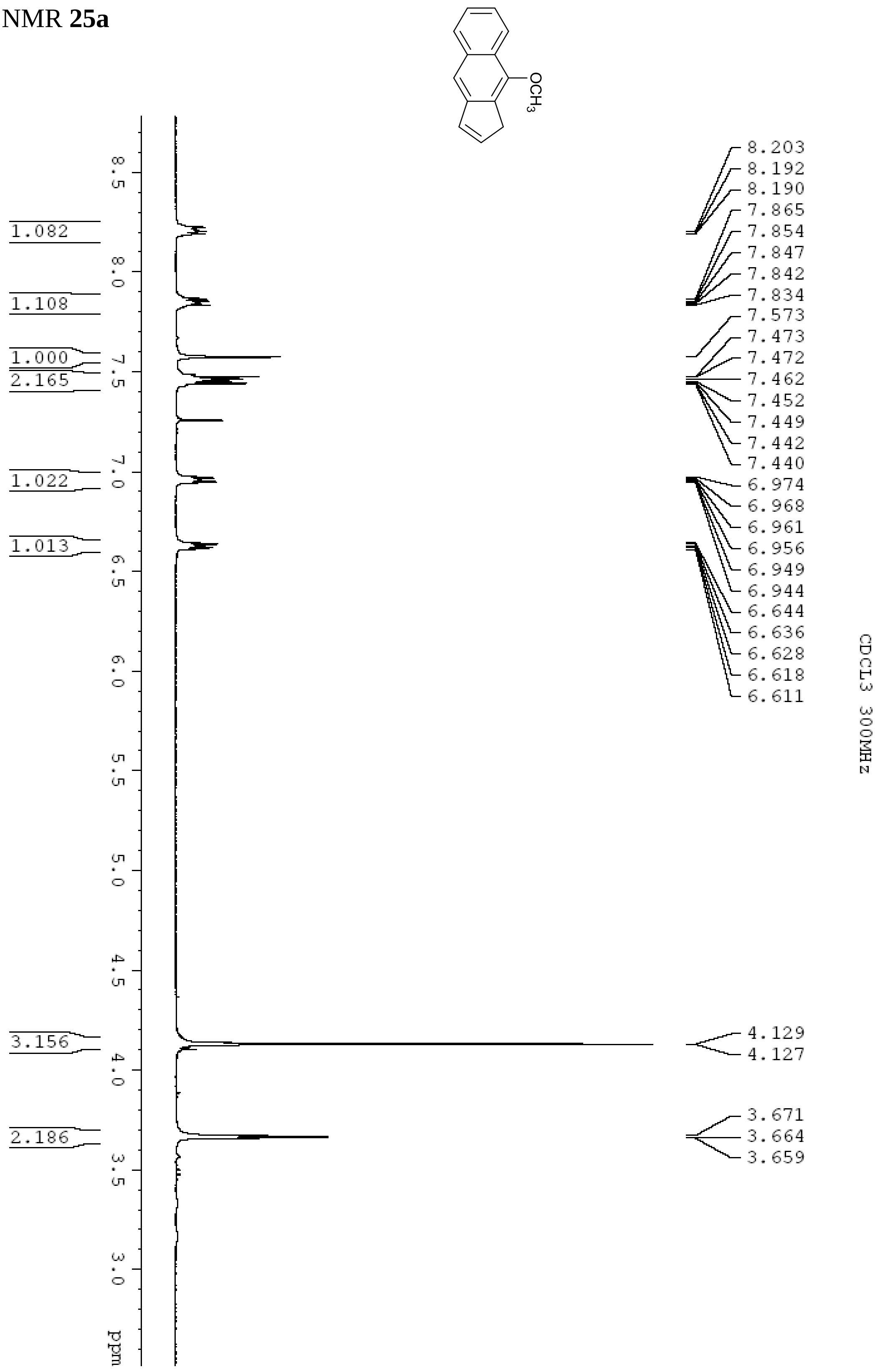
OCH3
8.203
8.192
8.190
7.865
7.854
7.847
7.842
7.834
7.573
7.473
7.472
7.462
7.452
7.449
7.442
7.440
6.974
6.968
6.961
6.956
6.949
6.944
6.644
6.636
6.628
6.618
6.611
4.129
4.127
3.671
3.664
3.659
CDCL3 300MHz
1.082
1.108
1.000
2.165
1.022
1.013
3.156
2.186
ppm

$^{13}C$ NMR **25a**

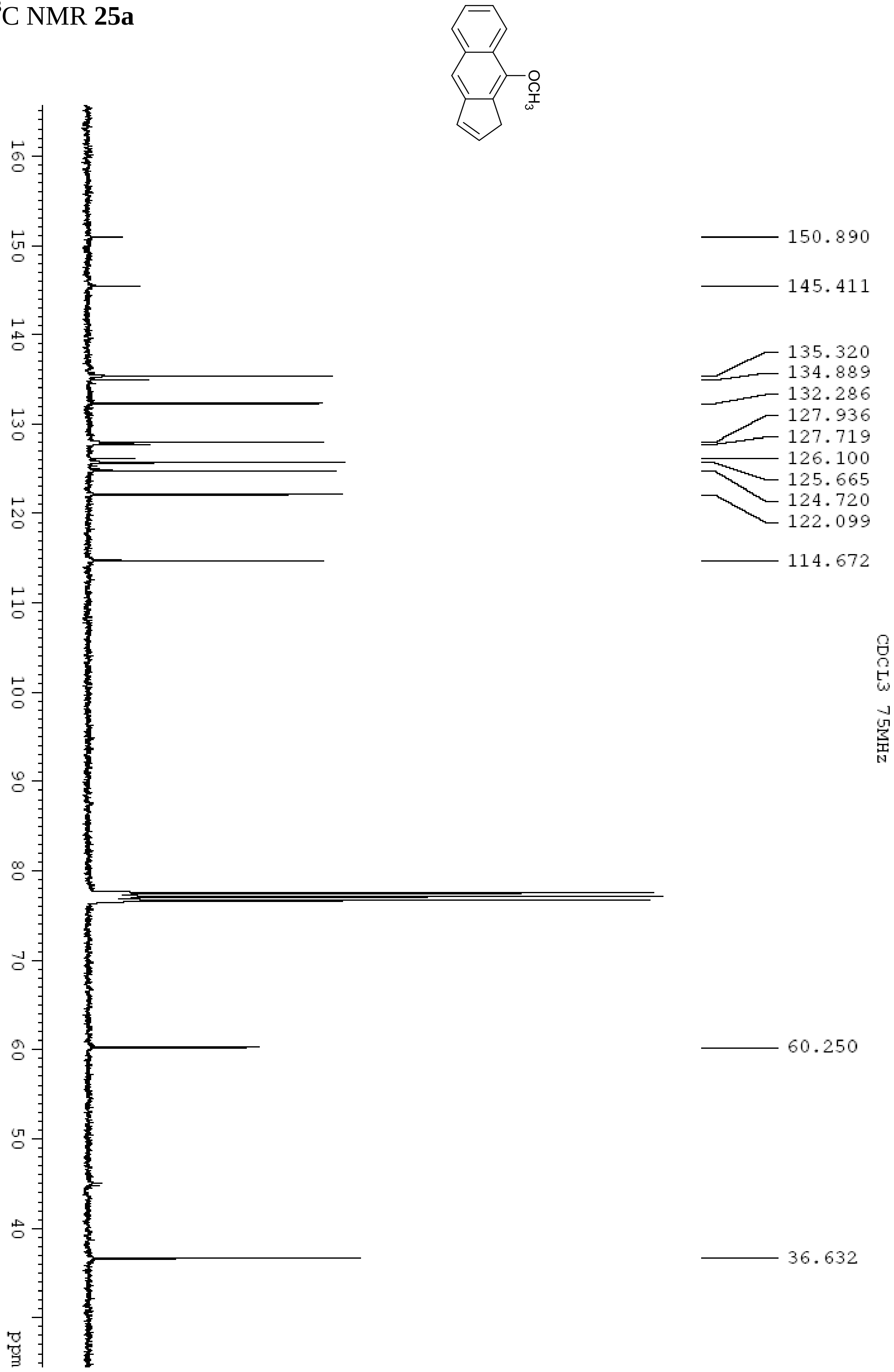
OCH3
150.890
145.411
135.320
134.889
132.286
127.936
127.719
126.100
125.665
124.720
122.099
114.672
60.250
36.632
CDCL3 75MHz
160
150
140
130
120
110
100
90
80
70
60
50
40
ppm

$^{1}$H NMR **25a** + **26a**

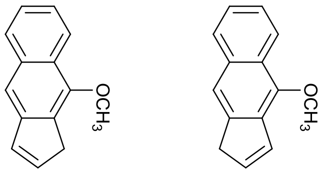


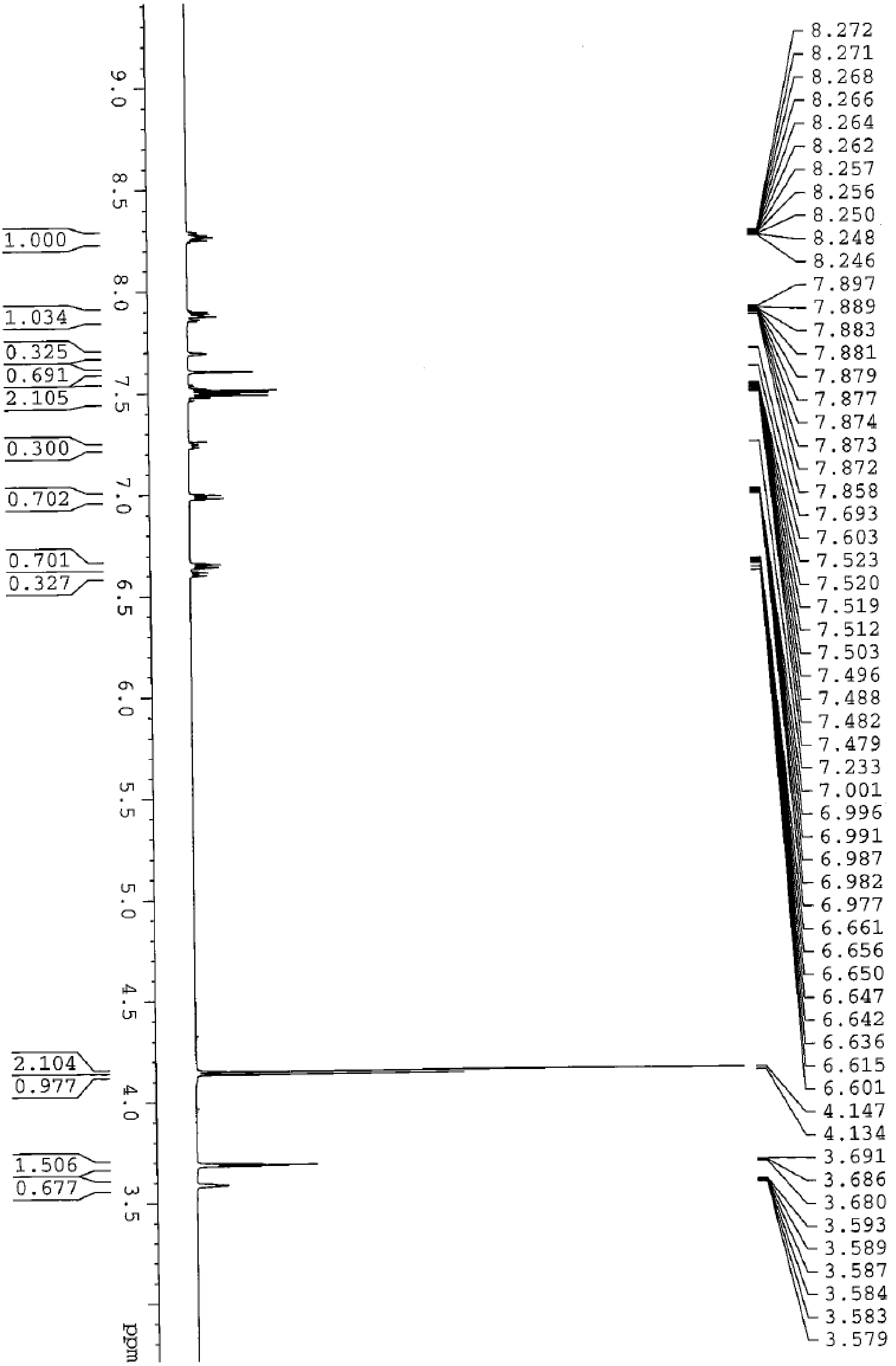

$^{13}$C NMR **25a** + **26a**

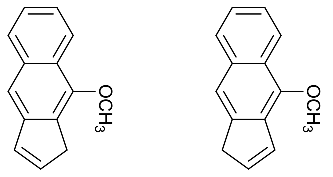


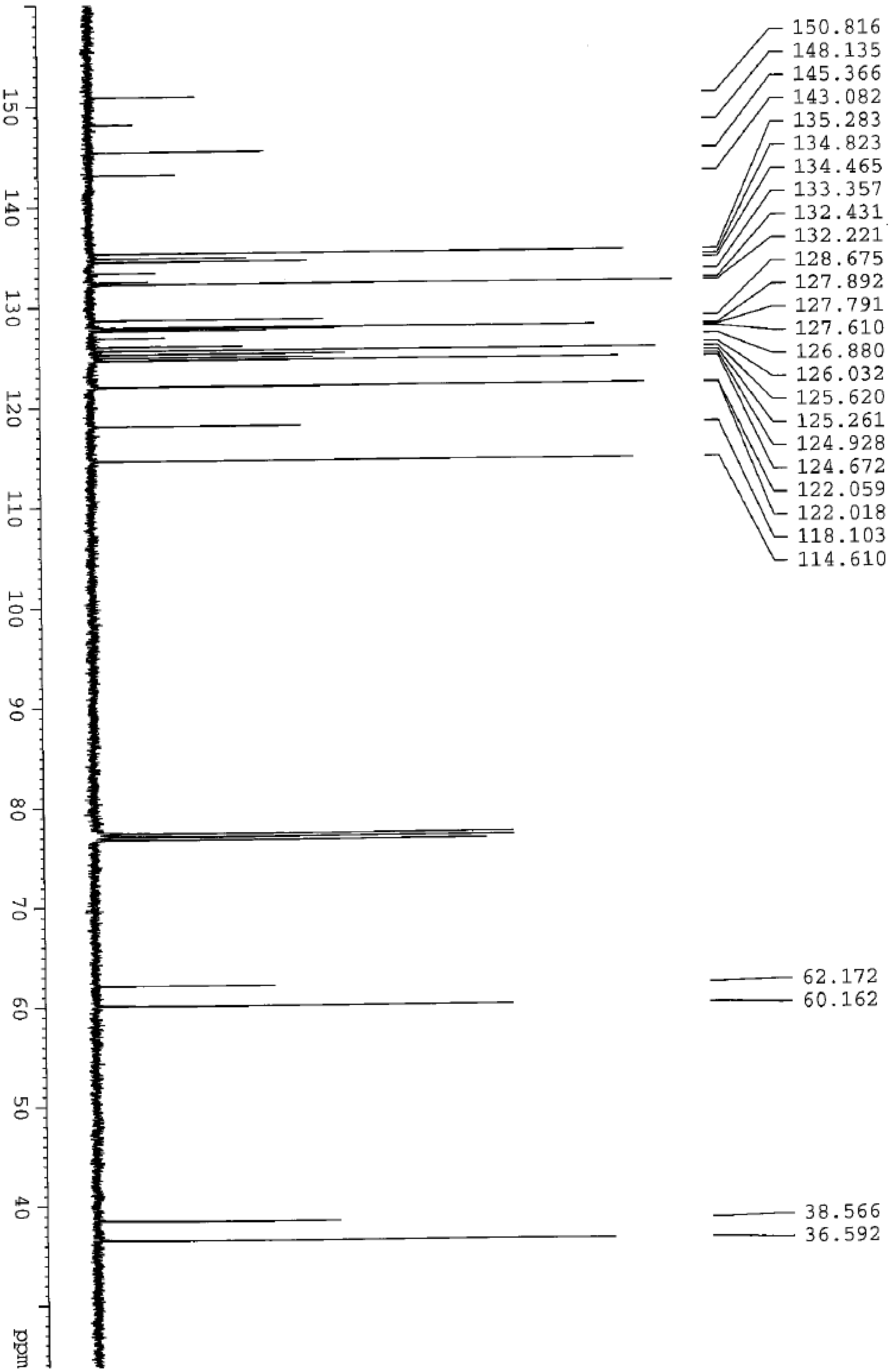

$^{1}$H NMR **25b**

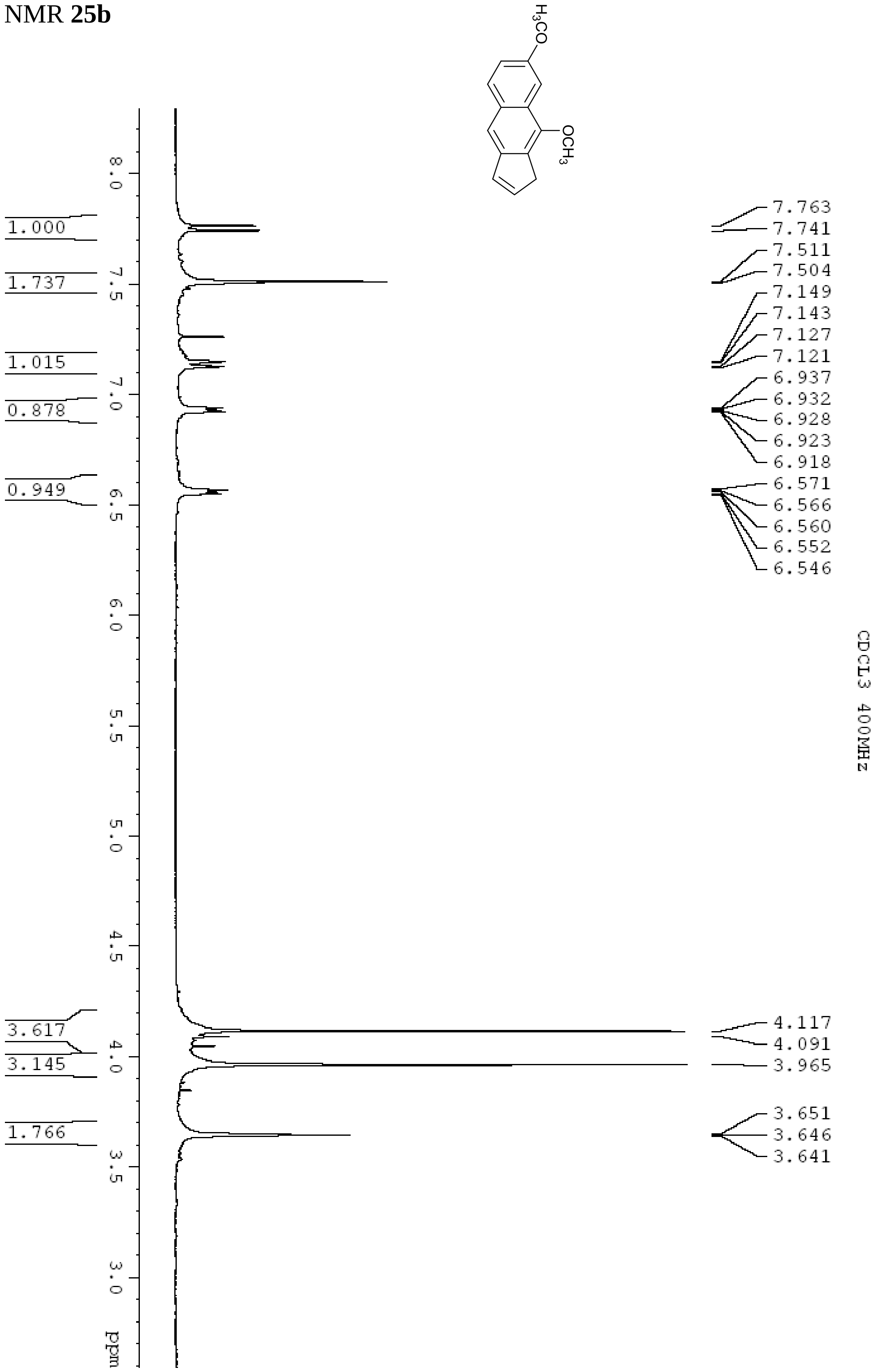
H3CO
OCH3
7.763
7.741
7.511
7.504
7.149
7.143
7.127
7.121
6.937
6.932
6.928
6.923
6.918
6.571
6.566
6.560
6.552
6.546
4.117
4.091
3.965
3.651
3.646
3.641
1.000
1.737
1.015
0.878
0.949
3.617
3.145
1.766
CDCL3 400MHz
ppm

$^{13}$C NMR **25b**

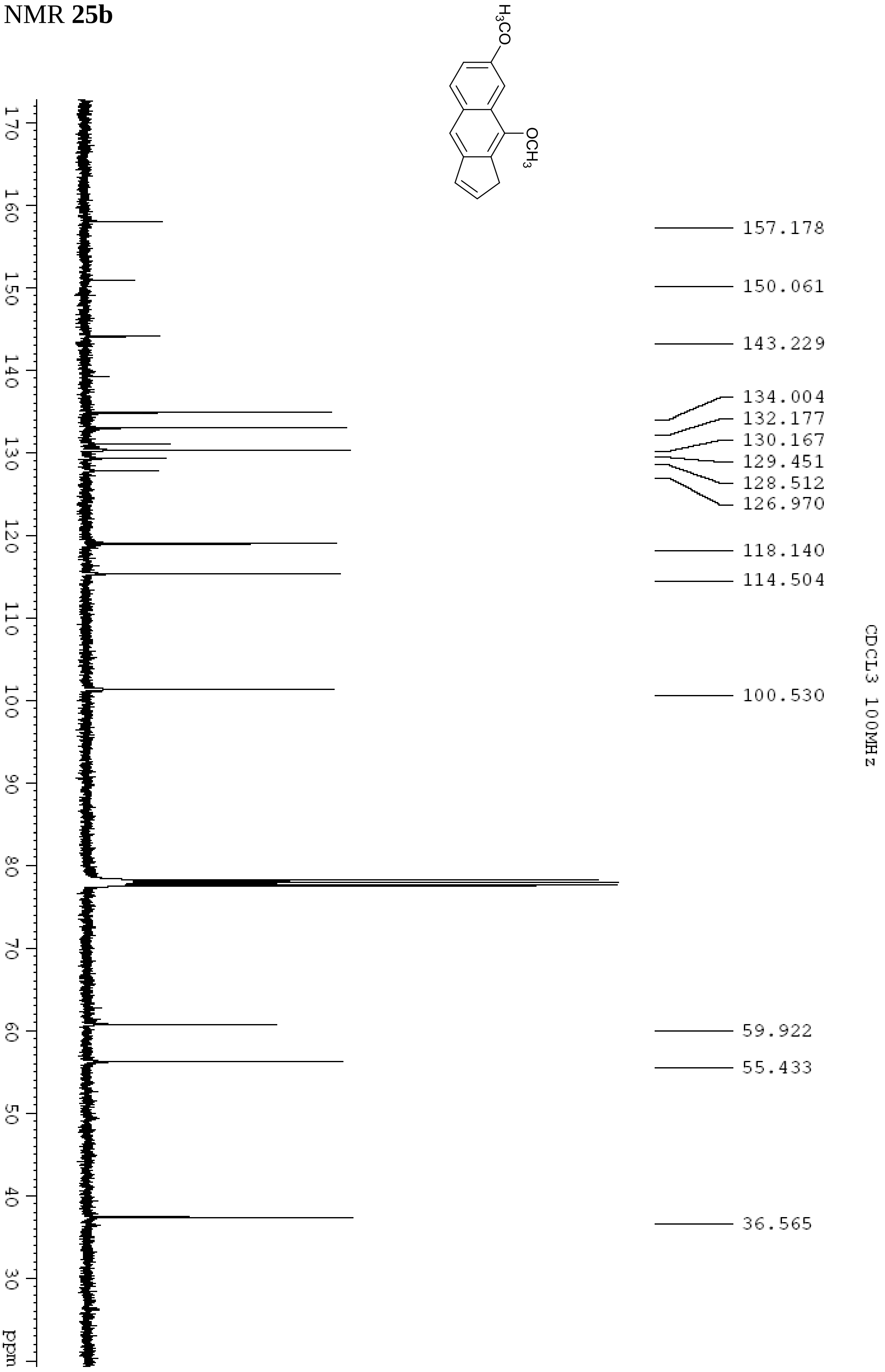
H3CO
OCH3
157.178
150.061
143.229
134.004
132.177
130.167
129.451
128.512
126.970
118.140
114.504
100.530
59.922
55.433
36.565
CDCL3 100MHz
ppm

$^{1}$H NMR **26b**

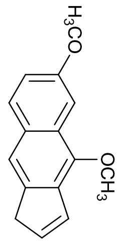

H3CO
OCH3


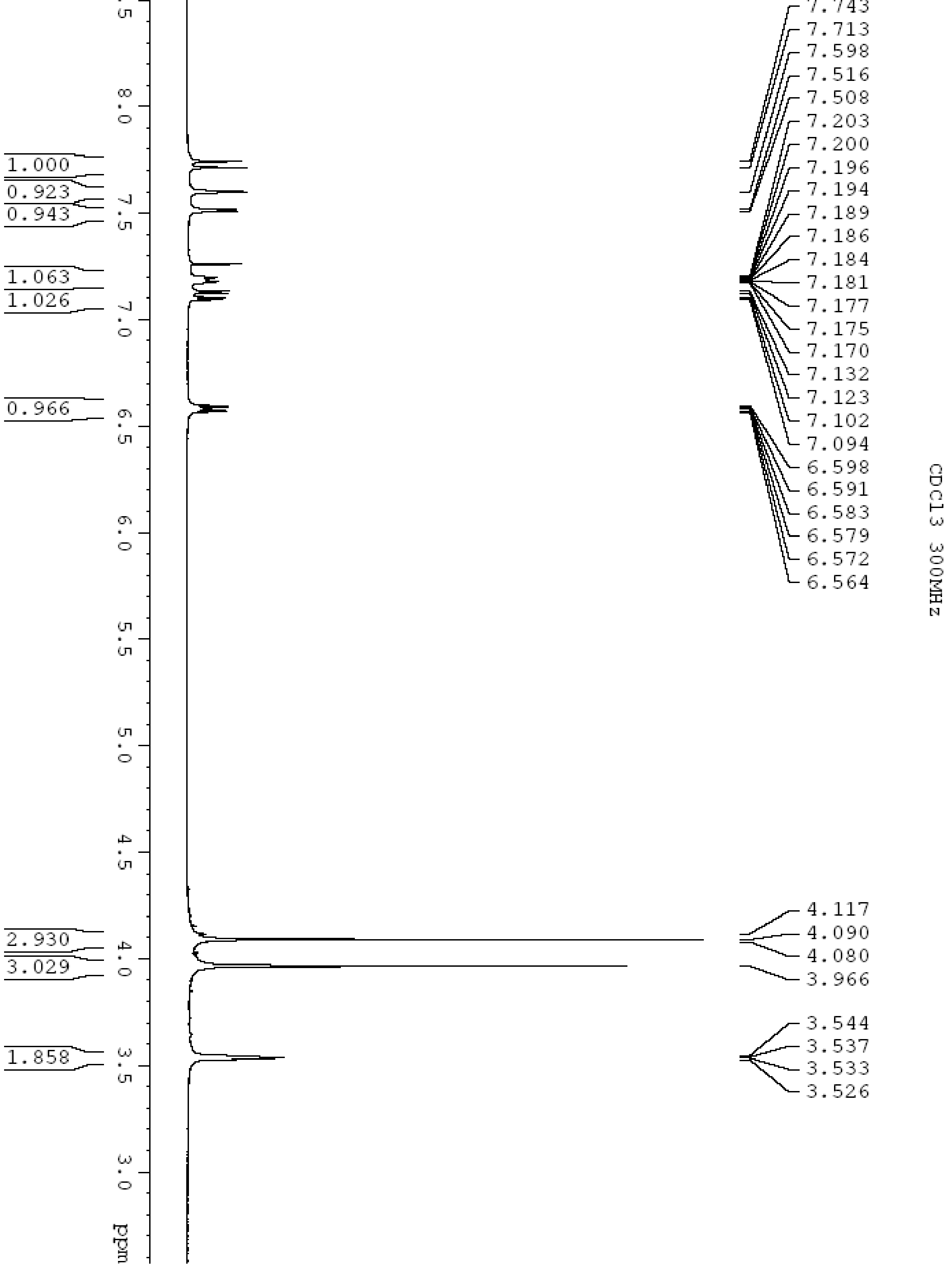

7.743
7.713
7.598
7.516
7.508
7.203
7.200
7.196
7.194
7.189
7.186
7.184
7.181
7.177
7.175
7.170
7.132
7.123
7.102
7.094
6.598
6.591
6.583
6.579
6.572
6.564
4.117
4.090
4.080
3.966
3.544
3.537
3.533
3.526
CDCl3 300MHz
1.000
0.923
0.943
1.063
1.026
0.966
2.930
3.029
1.858
8.5
8.0
7.5
7.0
6.5
6.0
5.5
5.0
4.5
4.0
3.5
3.0
ppm

$^{13}C$ NMR **26b**

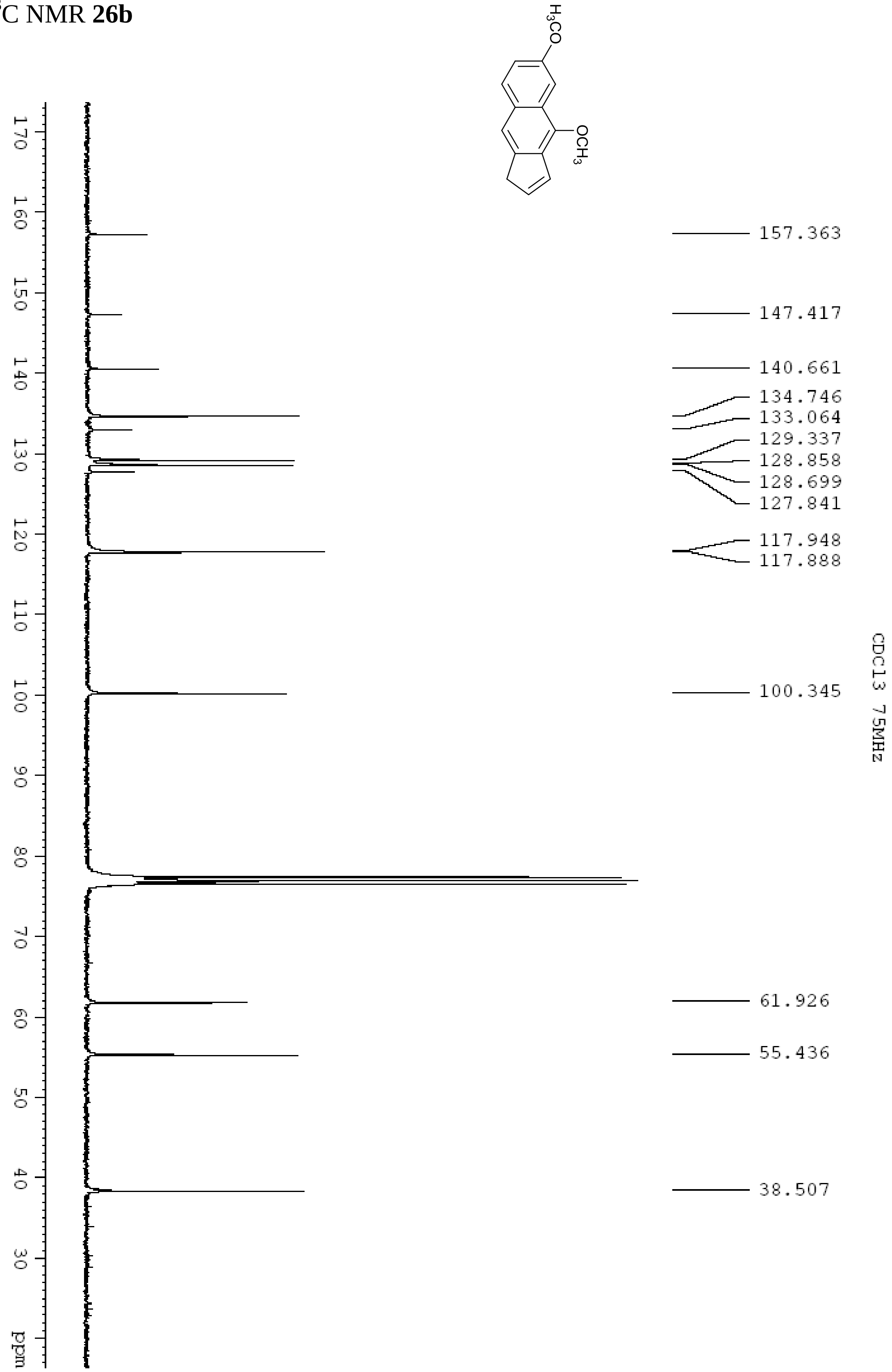
H3CO
OCH3
157.363
147.417
140.661
134.746
133.064
129.337
128.858
128.699
127.841
117.948
117.888
100.345
61.926
55.436
38.507
CDCl3 75MHz
170
160
150
140
130
120
110
100
90
80
70
60
50
40
30
ppm

$^{1}$H NMR **25c**

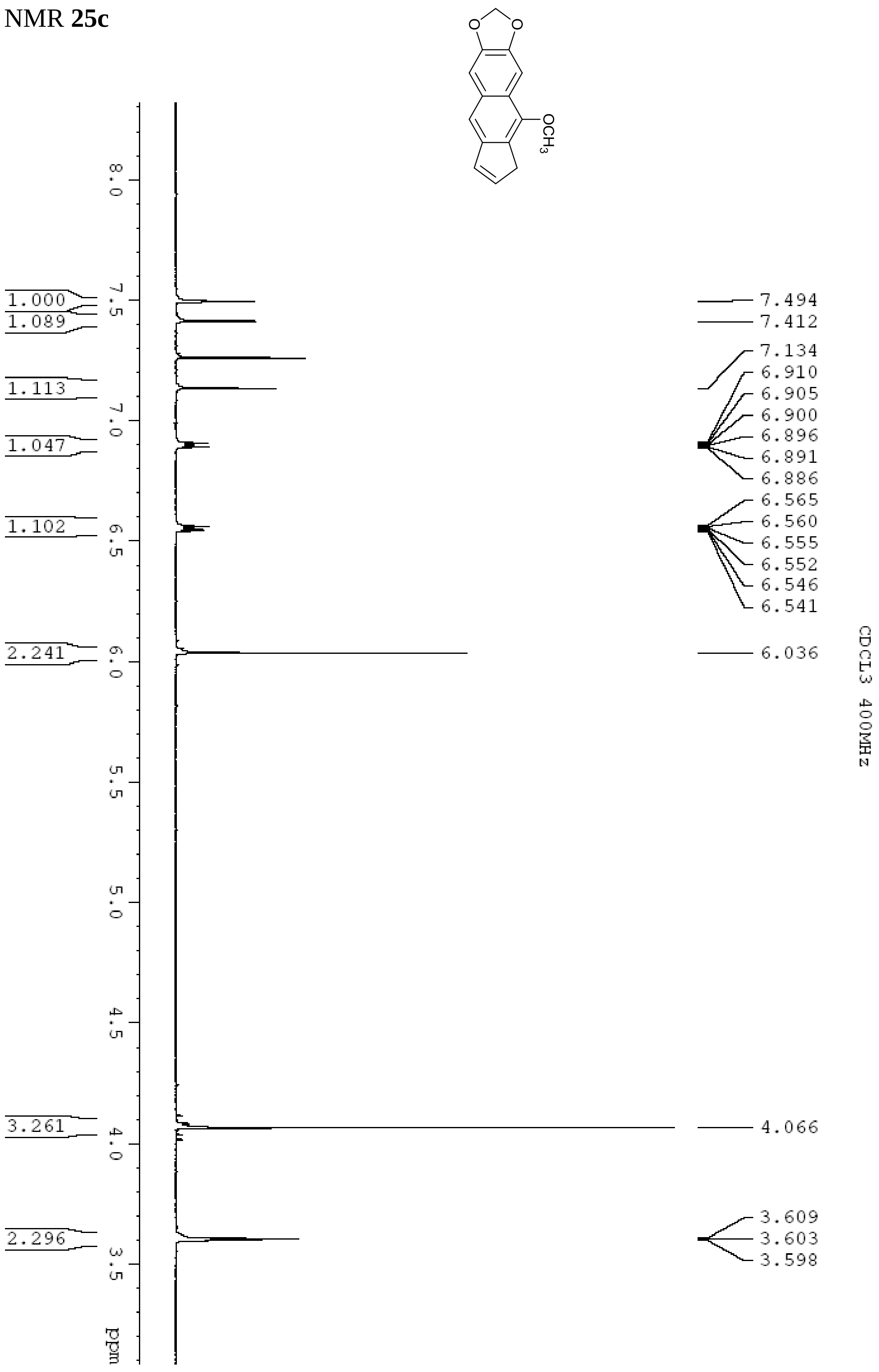
OCH3
CDCL3 400MHz
7.494
7.412
7.134
6.910
6.905
6.900
6.896
6.891
6.886
6.565
6.560
6.555
6.552
6.546
6.541
6.036
4.066
3.609
3.603
3.598
1.000
1.089
1.113
1.047
1.102
2.241
3.261
2.296
ppm

$^{13}$C NMR **25c**

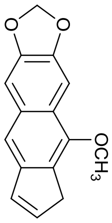

OCH3


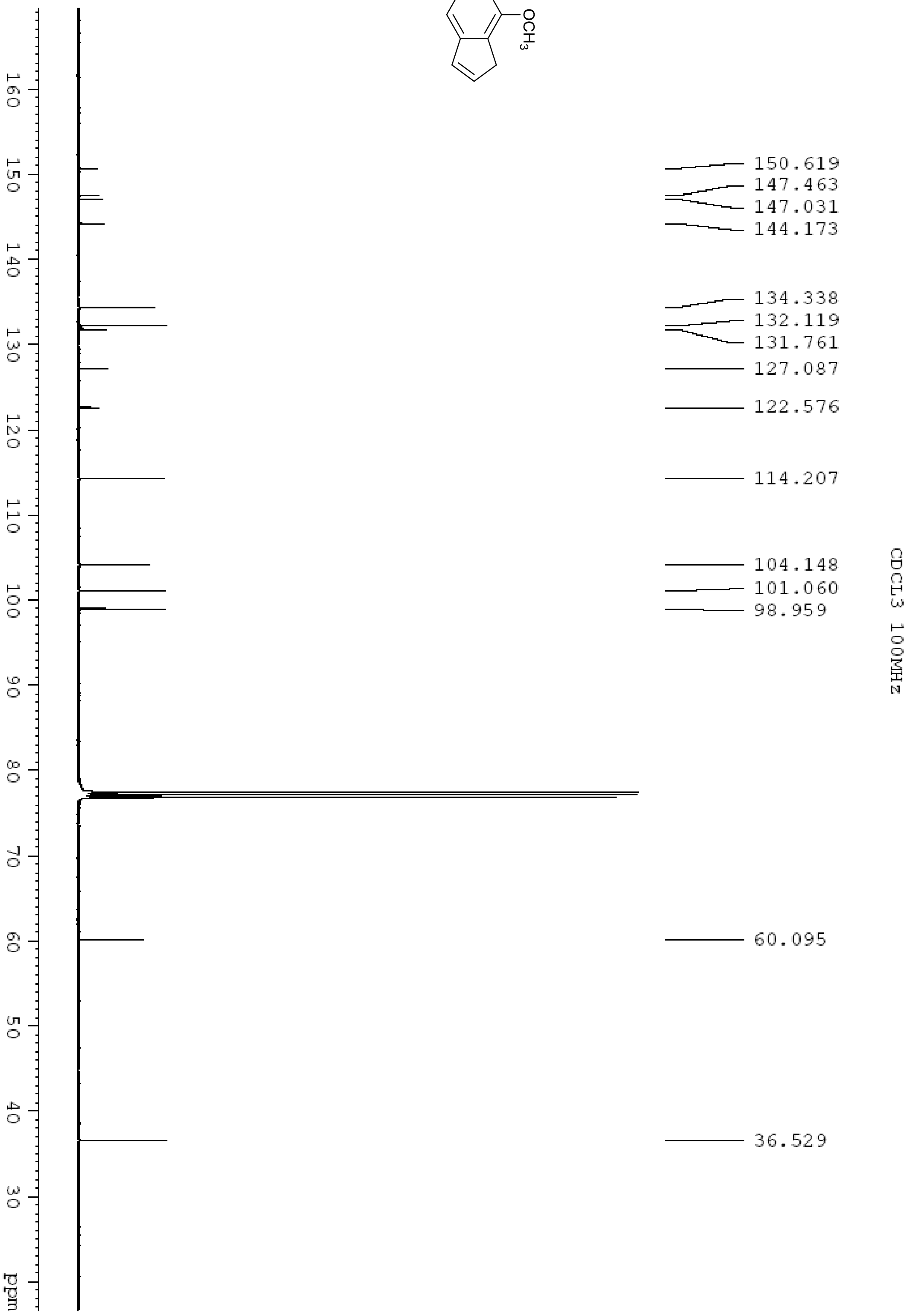

150.619
147.463
147.031
144.173
134.338
132.119
131.761
127.087
122.576
114.207
104.148
101.060
98.959
60.095
36.529
CDCL3 100MHz
ppm

$^{1}$H NMR **26c**

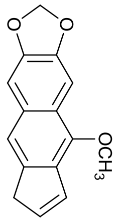

O
O
OCH3


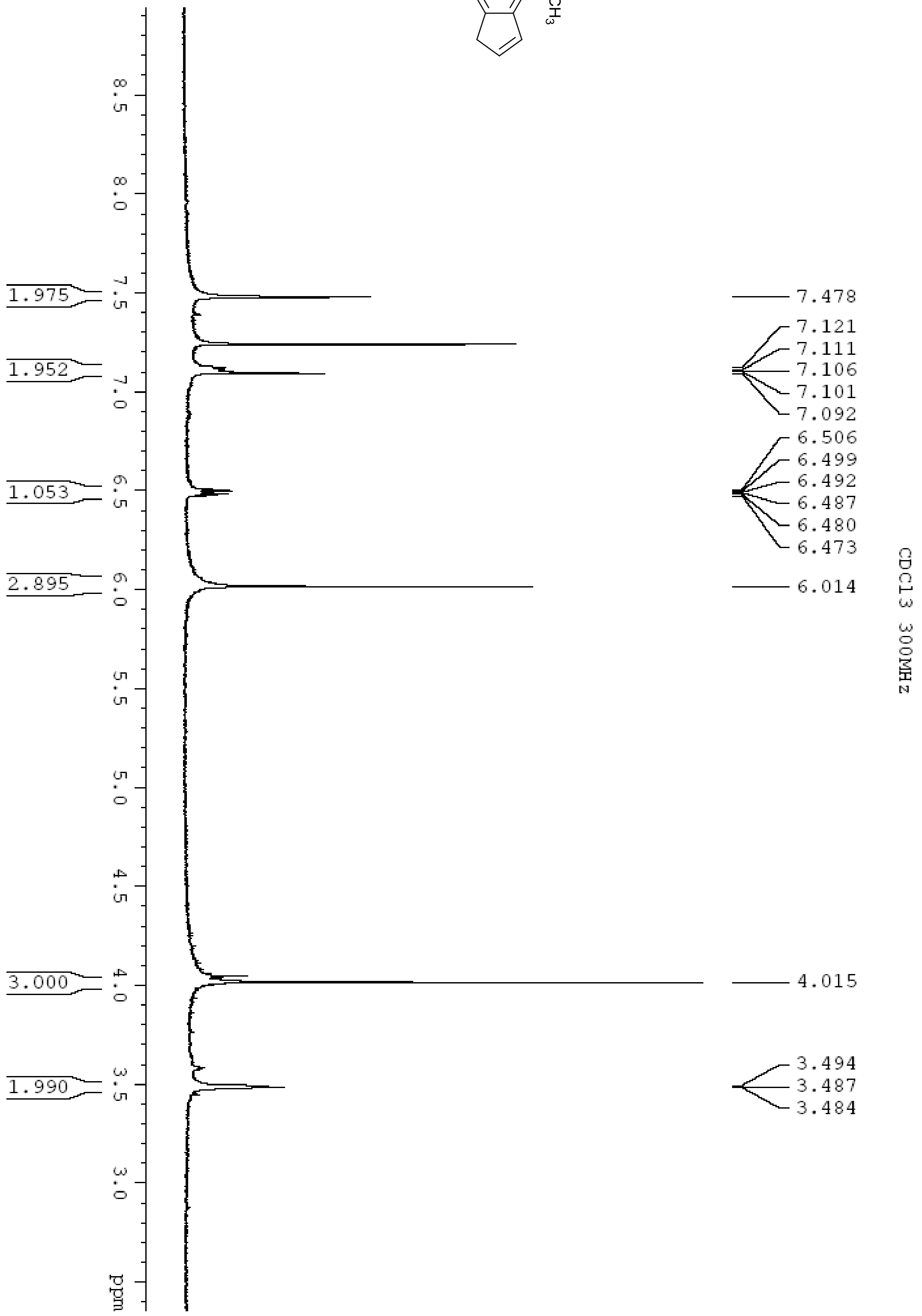

1.975
1.952
1.053
2.895
3.000
1.990
8.5
8.0
7.5
7.0
6.5
6.0
5.5
5.0
4.5
4.0
3.5
3.0
ppm
7.478
7.121
7.111
7.106
7.101
7.092
6.506
6.499
6.492
6.487
6.480
6.473
6.014
4.015
3.494
3.487
3.484
CDCl3 300MHz

$^{13}$C NMR **26c**

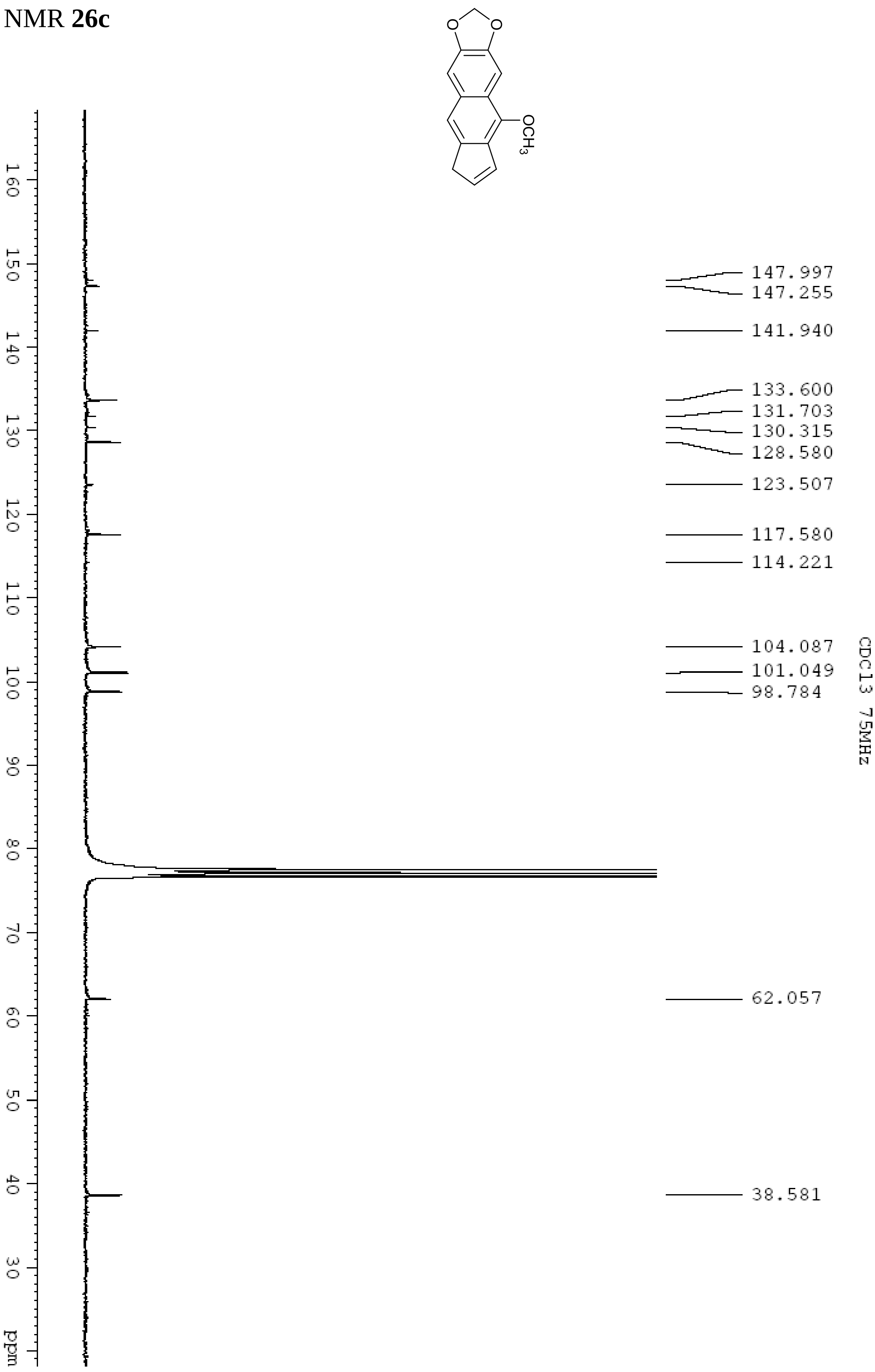
OCH3
147.997
147.255
141.940
133.600
131.703
130.315
128.580
123.507
117.580
114.221
104.087
101.049
98.784
62.057
38.581
CDCl3 75MHz
160
150
140
130
120
110
100
90
80
70
60
50
40
30
ppm

$^{1}$H NMR **25d** + **26d**

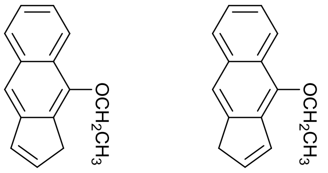


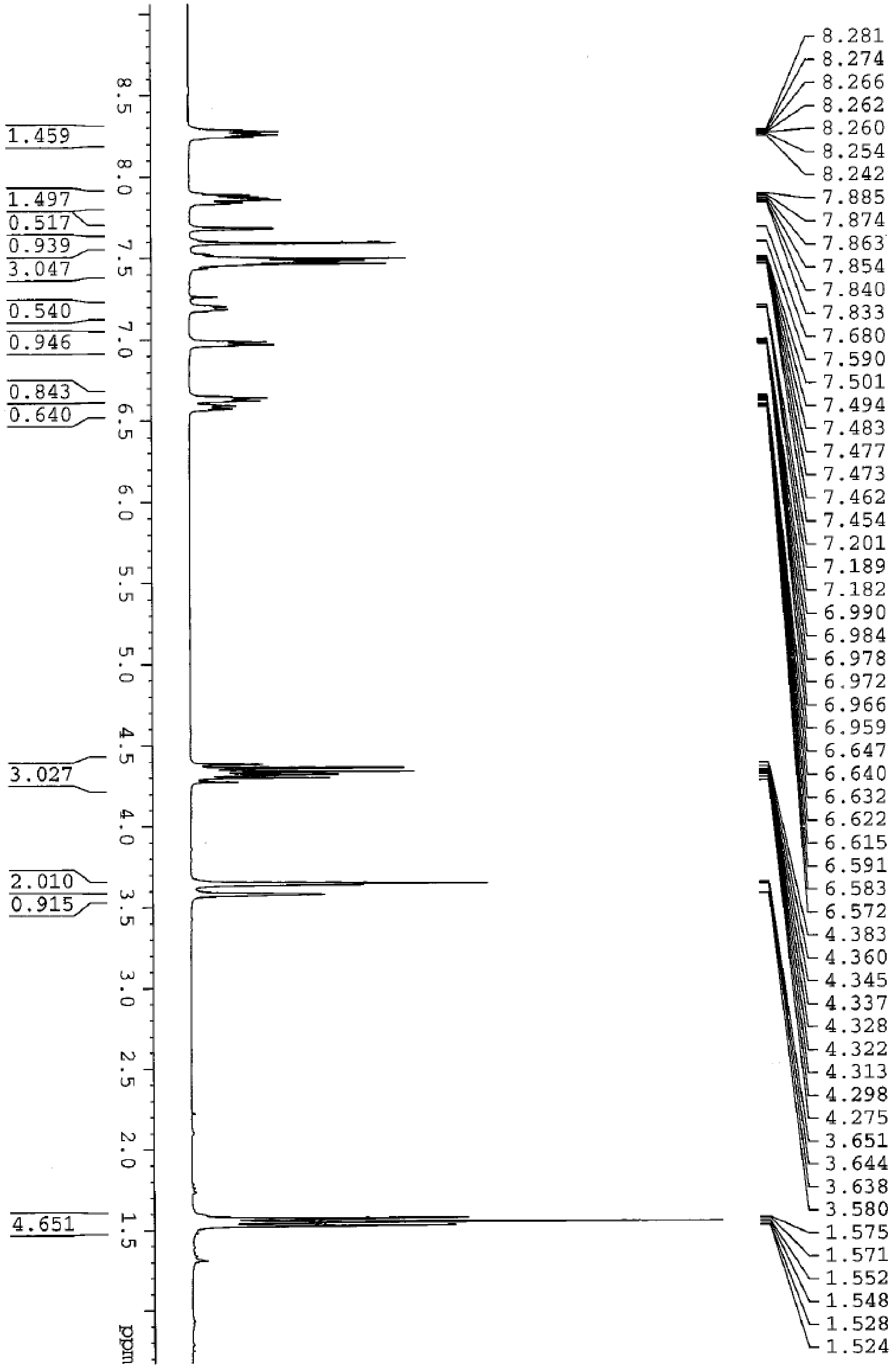

$^{13}$C NMR **25d** + **26d**

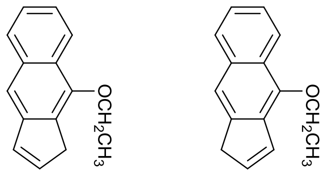


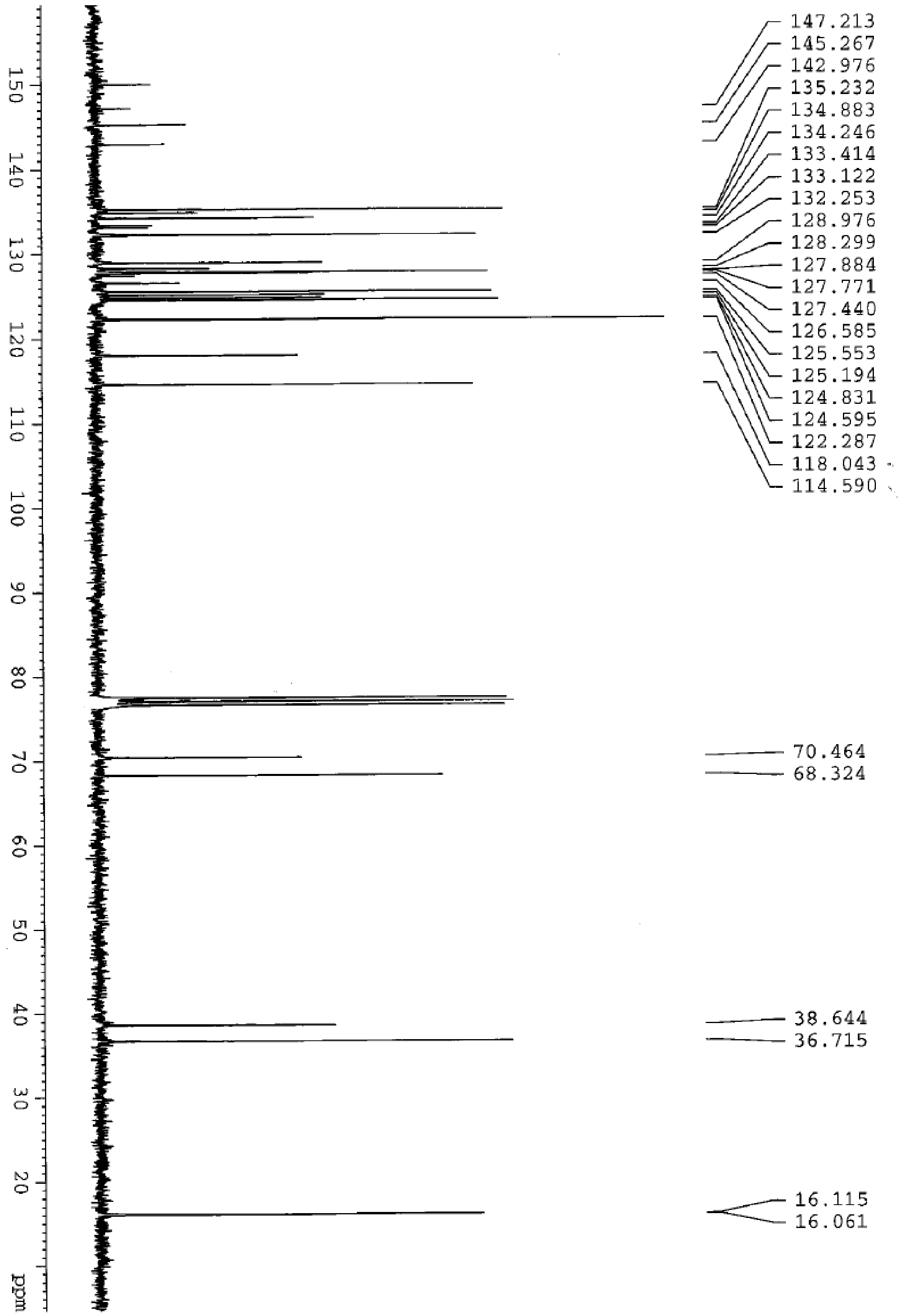

$^{1}$H NMR **18**

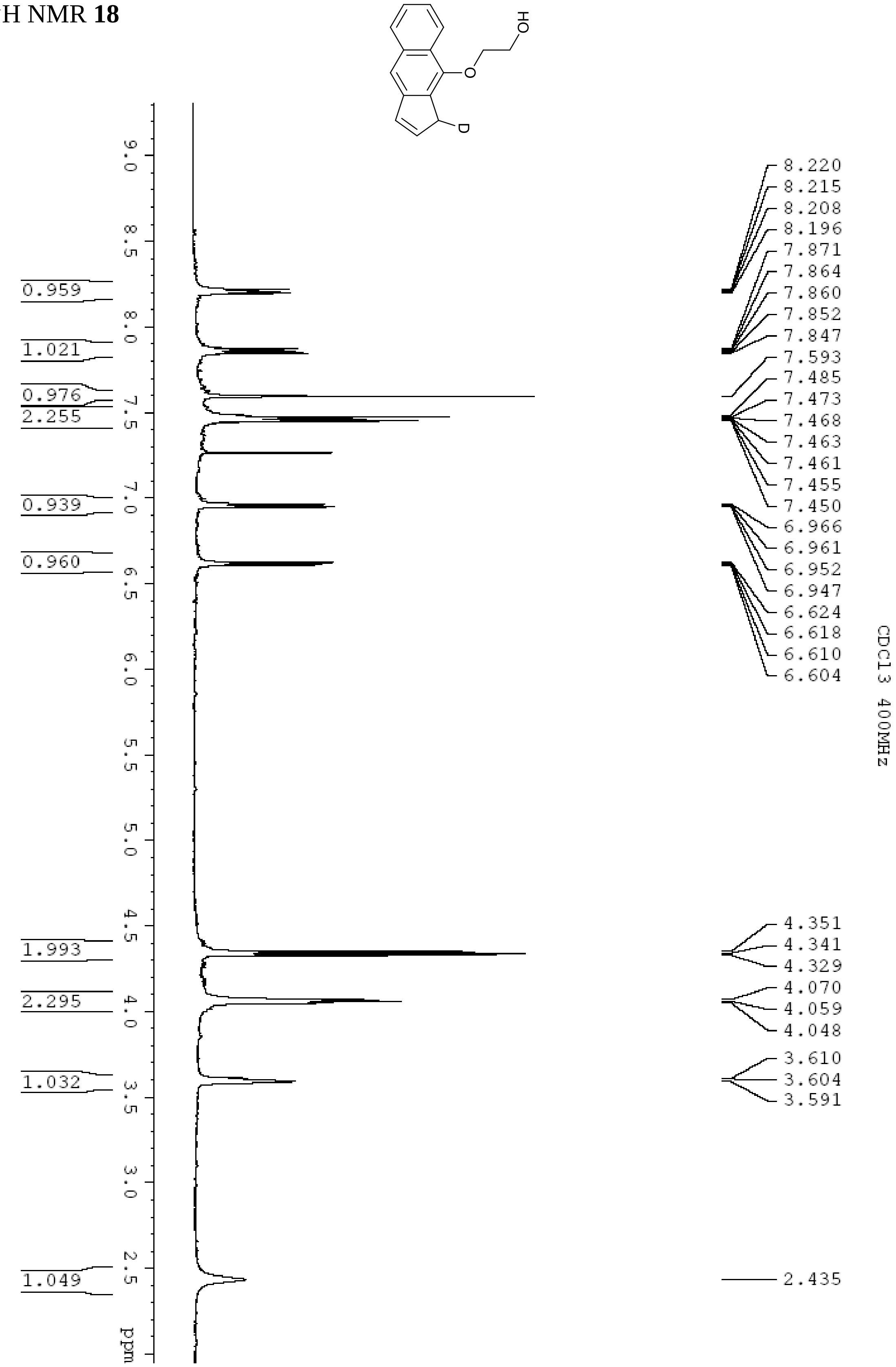
HO
O
D
8.220
8.215
8.208
8.196
7.871
7.864
7.860
7.852
7.847
7.593
7.485
7.473
7.468
7.463
7.461
7.455
7.450
6.966
6.961
6.952
6.947
6.624
6.618
6.610
6.604
4.351
4.341
4.329
4.070
4.059
4.048
3.610
3.604
3.591
2.435
CDCl3 400MHz
ppm

$^{13}$C NMR **18**

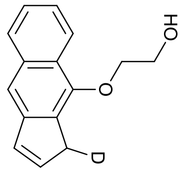

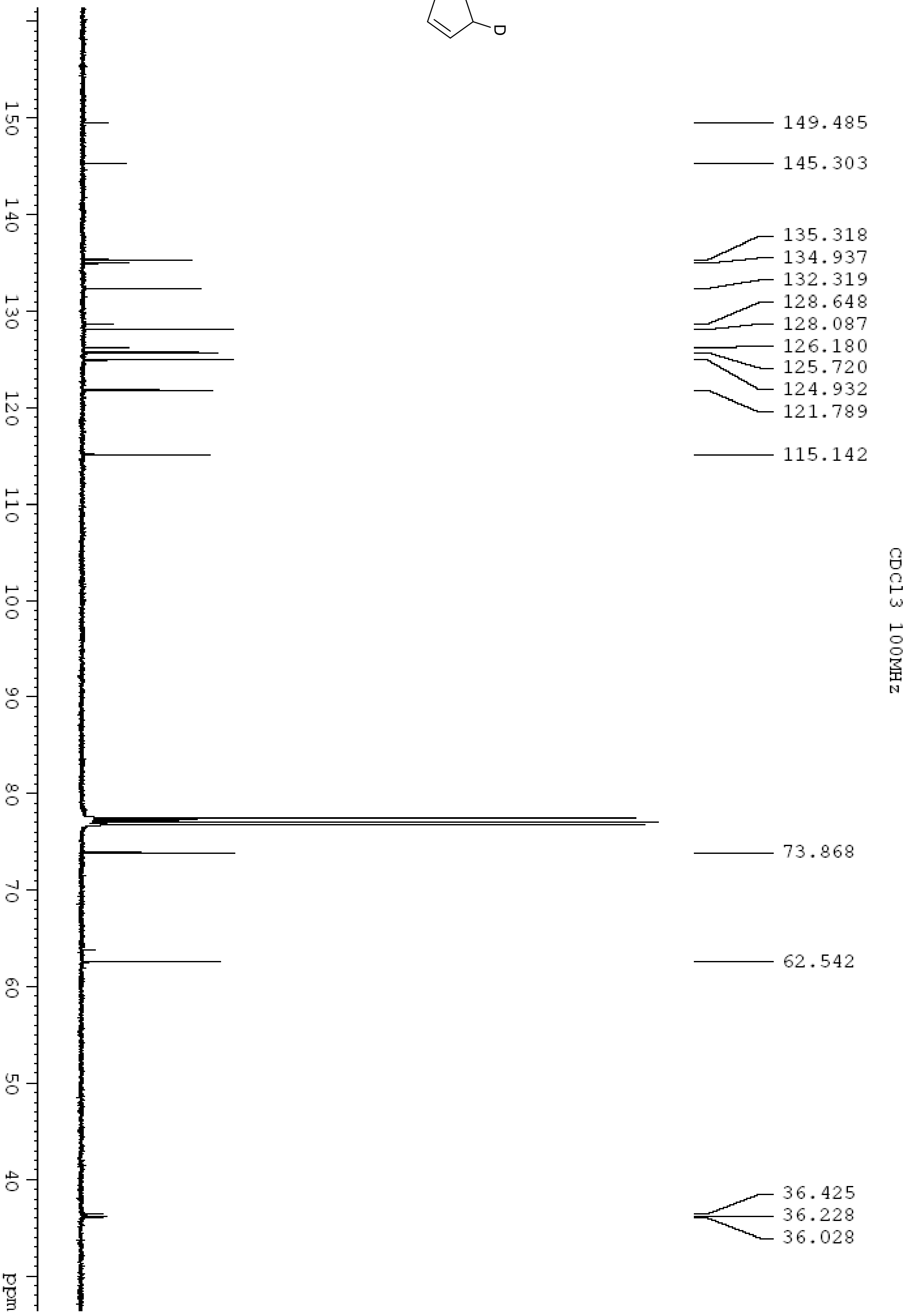
149.485
145.303
135.318
134.937
132.319
128.648
128.087
126.180
125.720
124.932
121.789
115.142
73.868
62.542
36.425
36.228
36.028
CDCl3 100MHz
ppm

$^{1}$H NMR **19**

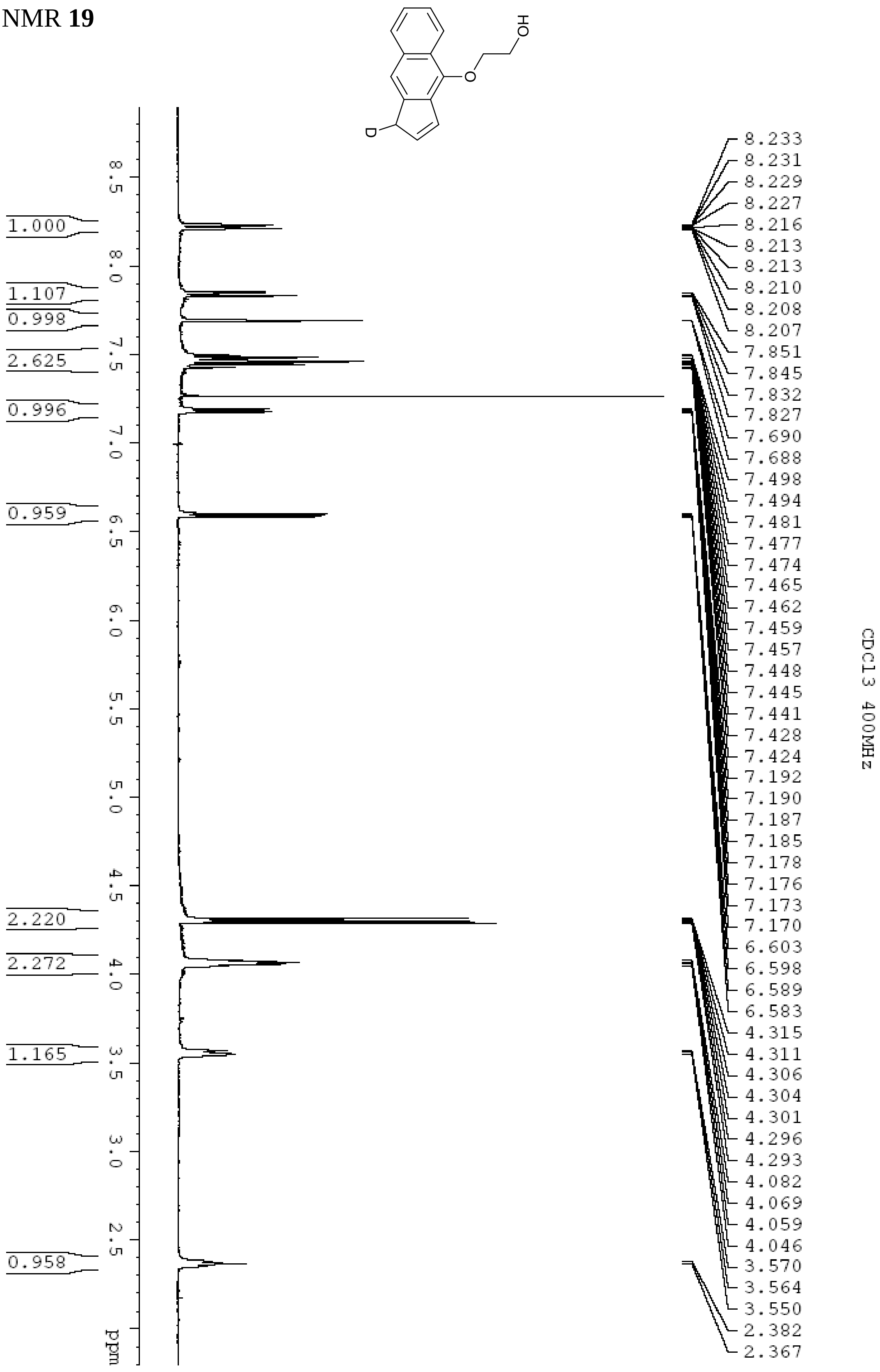

OH
O
D
8.5
8.0
7.5
7.0
6.5
6.0
5.5
5.0
4.5
4.0
3.5
3.0
2.5
ppm
1.000
1.107
0.998
2.625
0.996
0.959
2.220
2.272
1.165
0.958
8.233
8.231
8.229
8.227
8.216
8.213
8.213
8.210
8.208
8.207
7.851
7.845
7.832
7.827
7.690
7.688
7.498
7.494
7.481
7.477
7.474
7.465
7.462
7.459
7.457
7.448
7.445
7.441
7.428
7.424
7.192
7.190
7.187
7.185
7.178
7.176
7.173
7.170
6.603
6.598
6.589
6.583
4.315
4.311
4.306
4.304
4.301
4.296
4.293
4.082
4.069
4.059
4.046
3.570
3.564
3.550
2.382
2.367
CDCl3 400MHz

$^{13}$C NMR **19**

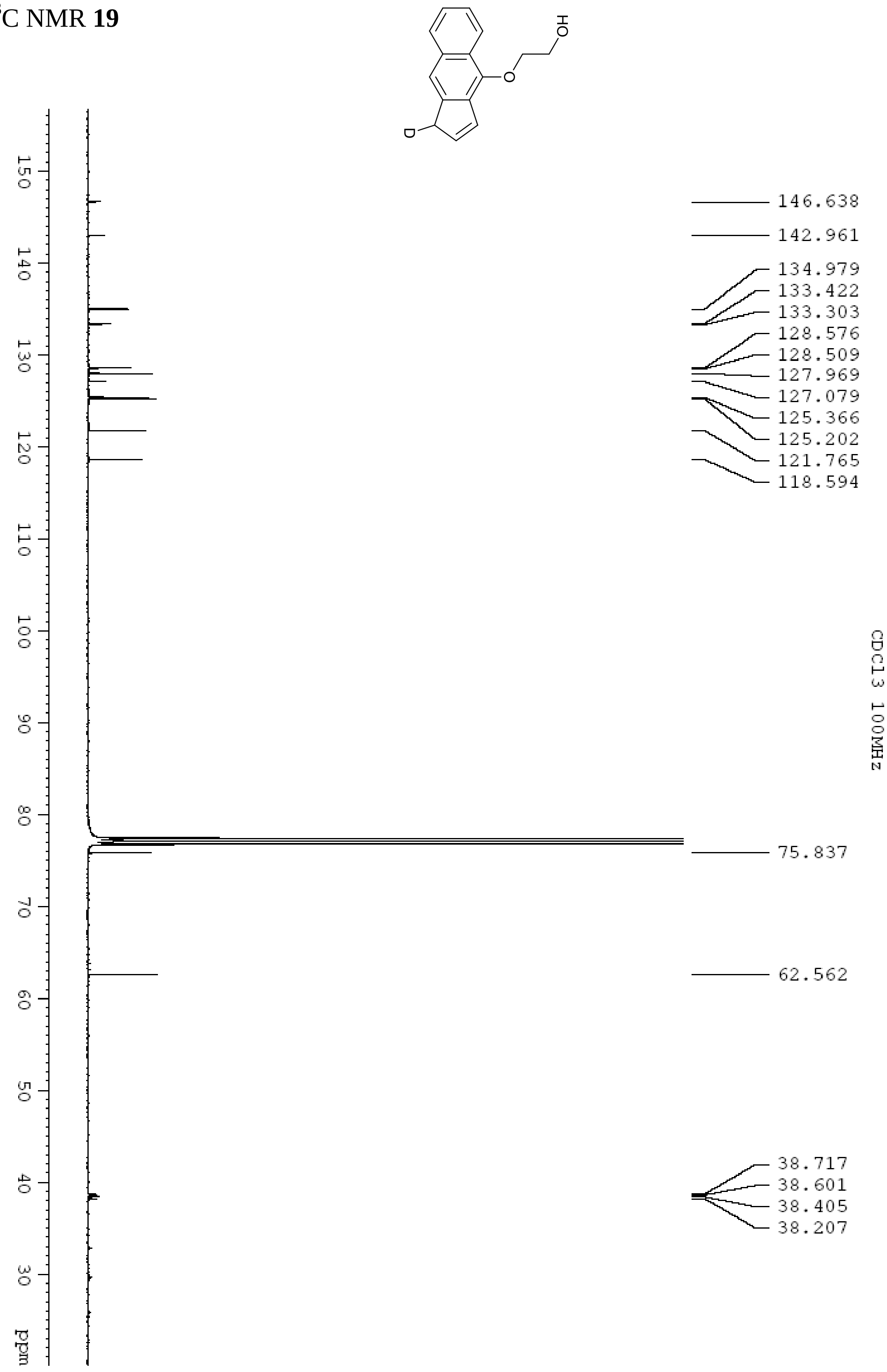

146.638
142.961
134.979
133.422
133.303
128.576
128.509
127.969
127.079
125.366
125.202
121.765
118.594
75.837
62.562
38.717
38.601
38.405
38.207
CDCl3 100MHz
ppm

$^{1}$H NMR **21**

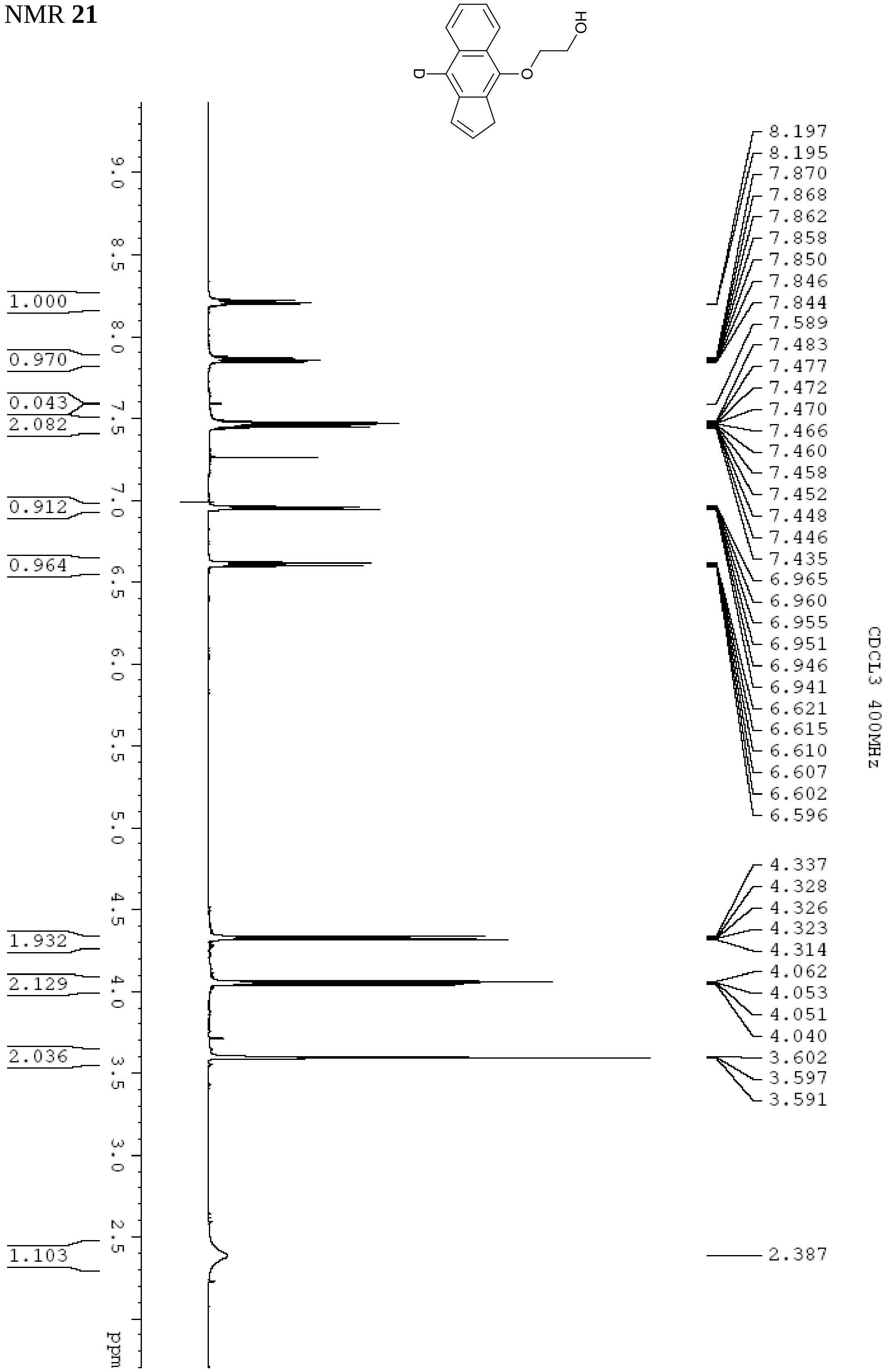

HO
O
D
8.197
8.195
7.870
7.868
7.862
7.858
7.850
7.846
7.844
7.589
7.483
7.477
7.472
7.470
7.466
7.460
7.458
7.452
7.448
7.446
7.435
6.965
6.960
6.955
6.951
6.946
6.941
6.621
6.615
6.610
6.607
6.602
6.596
4.337
4.328
4.326
4.323
4.314
4.062
4.053
4.051
4.040
3.602
3.597
3.591
2.387
CDCL3 400MHz
1.000
0.970
0.043
2.082
0.912
0.964
1.932
2.129
2.036
1.103
9.0
8.5
8.0
7.5
7.0
6.5
6.0
5.5
5.0
4.5
4.0
3.5
3.0
2.5
ppm

$^{13}$C NMR **21**

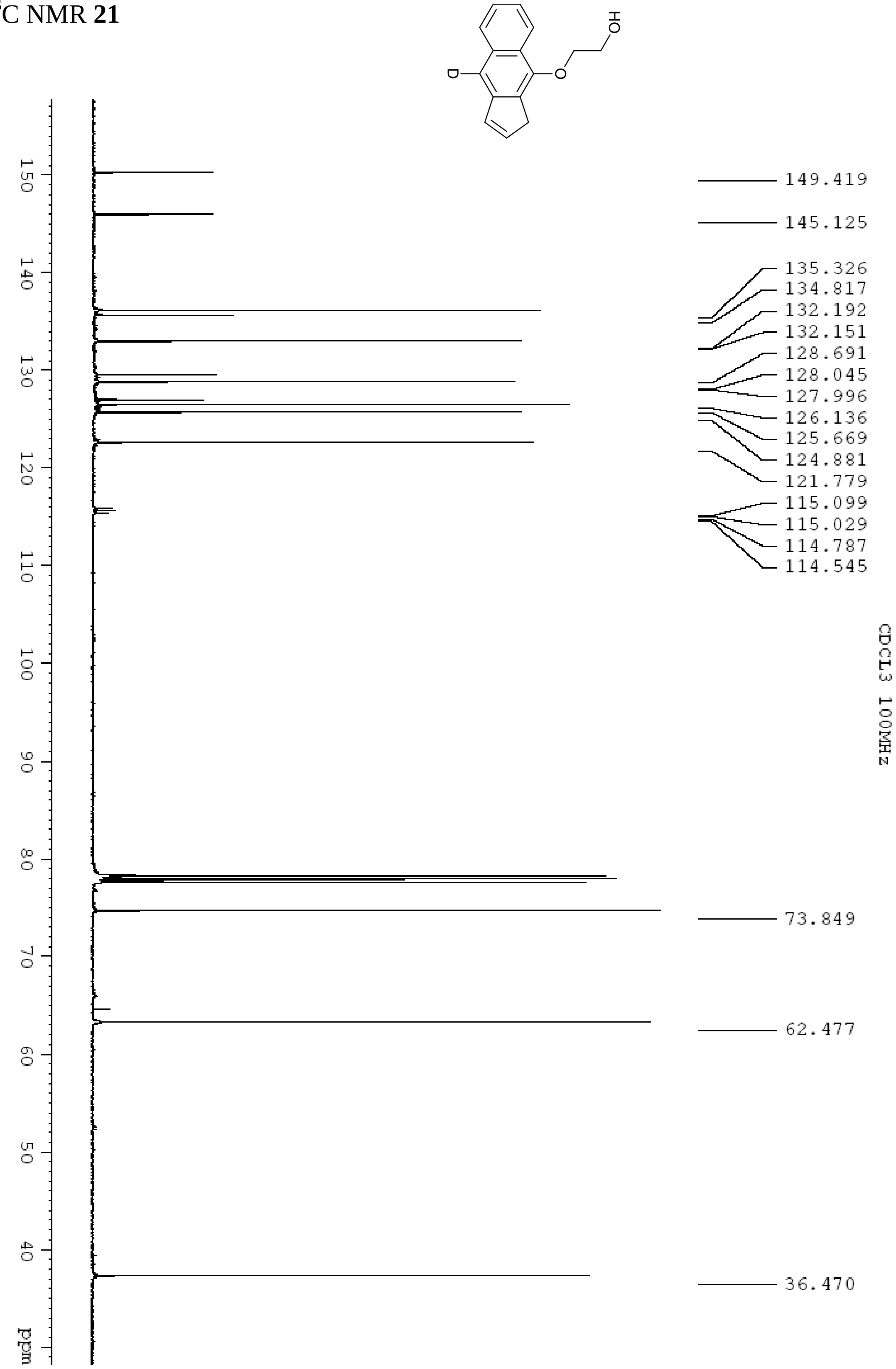
D
O
HO
149.419
145.125
135.326
134.817
132.192
132.151
128.691
128.045
127.996
126.136
125.669
124.881
121.779
115.099
115.029
114.787
114.545
73.849
62.477
36.470
CDCL3 100MHz
ppm

$^{1}$H NMR **22**

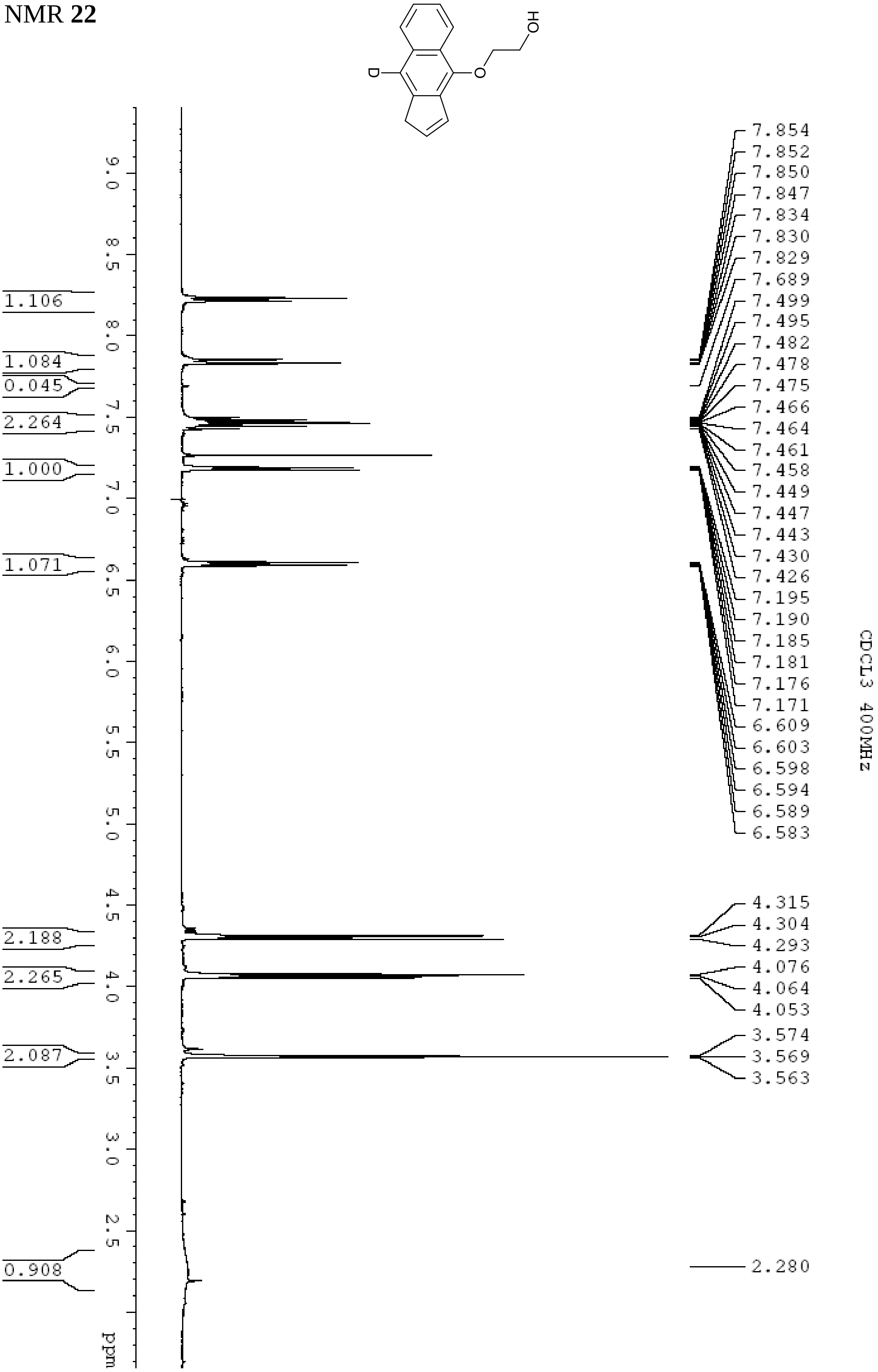
D
O
HO
7.854
7.852
7.850
7.847
7.834
7.830
7.829
7.689
7.499
7.495
7.482
7.478
7.475
7.466
7.464
7.461
7.458
7.449
7.447
7.443
7.430
7.426
7.195
7.190
7.185
7.181
7.176
7.171
6.609
6.603
6.598
6.594
6.589
6.583
4.315
4.304
4.293
4.076
4.064
4.053
3.574
3.569
3.563
2.280
CDCL3 400MHz
1.106
1.084
0.045
2.264
1.000
1.071
2.188
2.265
2.087
0.908
9.0
8.5
8.0
7.5
7.0
6.5
6.0
5.5
5.0
4.5
4.0
3.5
3.0
2.5
ppm

$^{13}$C NMR **22**

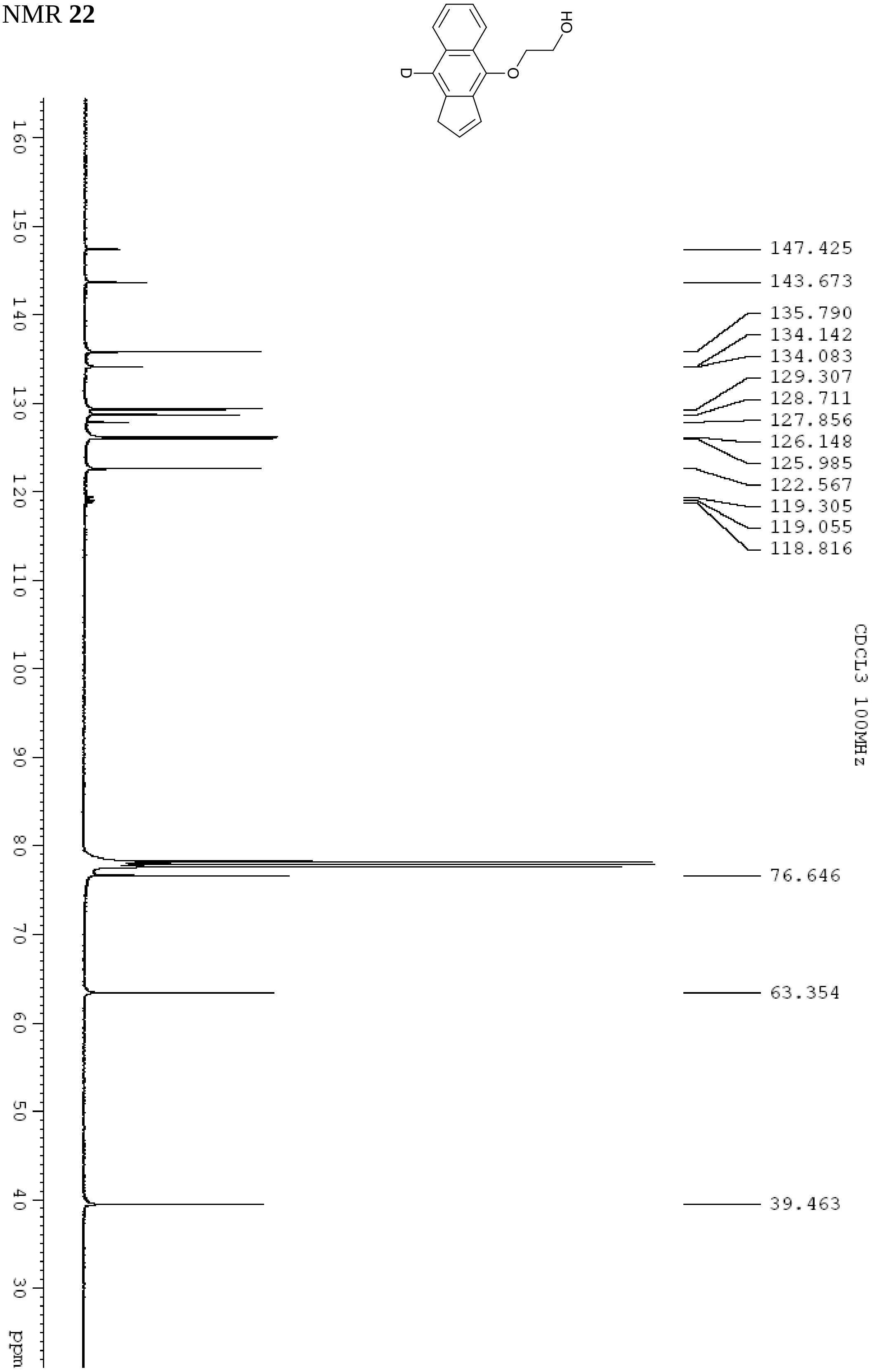
HO
O
D
147.425
143.673
135.790
134.142
134.083
129.307
128.711
127.856
126.148
125.985
122.567
119.305
119.055
118.816
CDCL3 100MHz
76.646
63.354
39.463
ppm

NOE experiment for **5b**

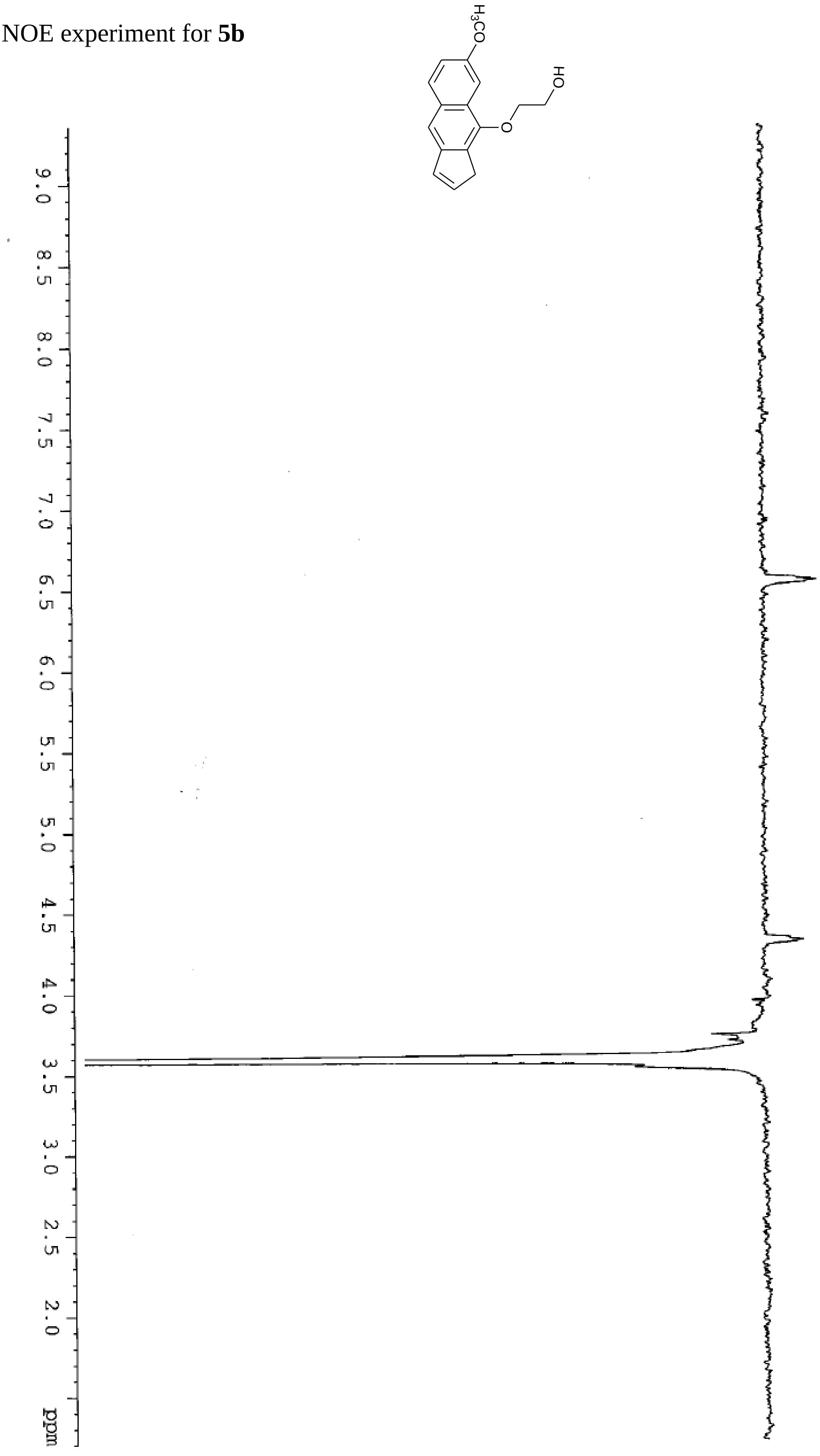

NOE experiment for **6b**

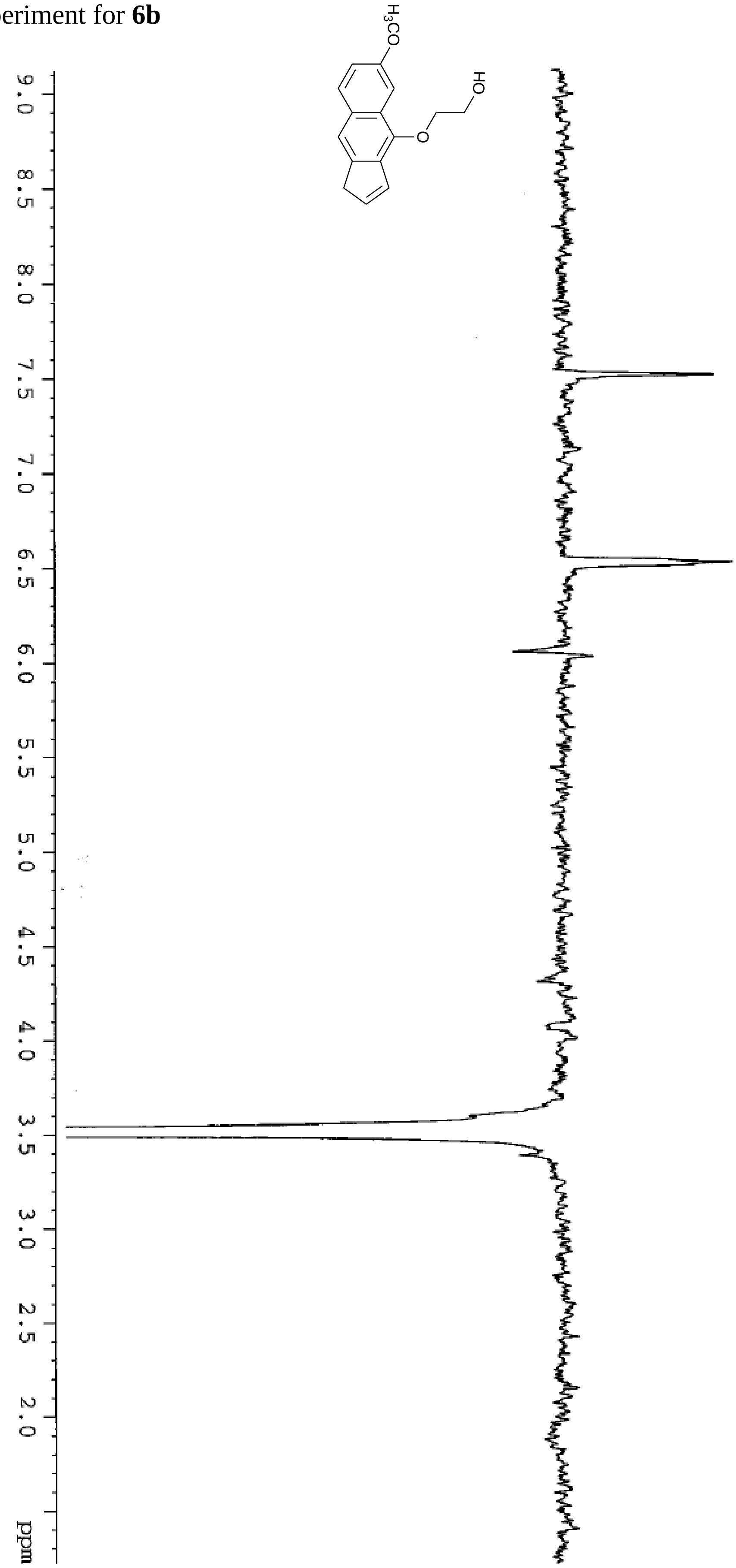

Supplement: File 1 — Experimental part. [file Beilstein_J_Org_Chem-12-260-s001.pdf]
