# Supplementary material for: Tandem processes promoted by a hydrogen shift in 6-arylfulvenes bearing acetalic units at ortho position: a combined experimental and computational study
Source: Beilstein J Org Chem. 2016 Feb 11;12:260–70. doi: 10.3762/bjoc.12.28 (PMC4778534; doi:10.3762/bjoc.12.28)
Supplement: File 2 — Computational part. [file Beilstein_J_Org_Chem-12-260-s002.pdf]

Supporting Information

for

Tandem processes promoted by a hydrogen shift in 6-arylfulvenes bearing acetalic units at *ortho* position: a combined experimental and computational study

Mateo Alajarin[1], Marta Marin-Luna[1], Pilar Sanchez-Andrada*[2] and Angel Vidal[1]

Address: [1]Departamento de Química Orgánica, Universidad de Murcia, Facultad de Química, Regional Campus of International Excellence "Campus Mare Nostrum", Espinardo, 30100 Murcia (Spain) and [2]University Centre of Defence at the Spanish Air Force Academy, Base Aerea de San Javier, C/ Coronel López Peña s/n, 30720, Santiago de la Ribera, Murcia, Spain

Email: Pilar Sanchez-Andrada - pilar.sanchez@cud.upct.es

*Corresponding author

Computational part

Contents	page
**Computational details**	**s2**
**Table S1.** Electronic energies of the stationary points found in the conversions **3a → 5a + 6a** and **5a → 27a → 6a** calculated at the B3LYP/6-31+G** and PCM-B3LYP/6-31+G** theoretical levels.	**s3**
**Table S2.** Relative electronic (*E*) and Gibbs (*G*) energies for the stationary points found in the conversions **3a → 5a + 6a** and **5a → 27a → 6a** calculated at B3LYP/6-31+G** and PCM-B3LYP/6-31+G** theoretical levels.	**s4**
**Cartesian coordinates and geometries** of the stationary points found in the transformations **3a → 5a + 6a** and **5a → 27a → 6a** optimized at the B3LYP/6-31+G** theoretical level.	**s5–s27**

Computational details

All calculations were carried out with the Gaussian09[1] suite of programs. An intensive characterization of the potential energy surface was done at the HF/6-31G*[2] theoretical level and then with the hybrid three-parameter functional customarily denoted as B3LYP[3] using the 6-31+G** basis set. All the reported stationary points were fully optimized by analytical gradient techniques. Harmonic frequency calculations at each level of theory verified the identity of each stationary point as a minimum or a transition state, and were used to provide an estimation of the zero-point vibrational energies (ZPVE), which were not scaled. The intrinsic reaction coordinates (IRC)[4] were followed to verify the energy profiles connecting each transition state to the correct local minima, by using the second-order Gonzalez–Schlegel integration method.[5]

To evaluate the influence of the DMSO solvent ($\varepsilon = 47$), we have optimized all the stationary points at the B3LYP/6-31+G** calculations using a Self-Consistency Reaction Field (SCRF)[6] method, based on the Polarized Continuum Model (PCM)[7] of Tomasi and co-workers.

---

[1] Gaussian 09, Revision A.02, M. J. Frisch, G. W. Trucks, H. B. Schlegel, G. E. Scuseria, M. A. Robb, J. R. Cheeseman, G. Scalmani, V. Barone, B. Mennucci, G. A. Petersson, H. Nakatsuji, M. Caricato, X. Li, H. P. Hratchian, A. F. Izmaylov, J. Bloino, G. Zheng, J. L. Sonnenberg, M. Hada, M. Ehara, K. Toyota, R. Fukuda, J. Hasegawa, M. Ishida, T. Nakajima, Y. Honda, O. Kitao, H. Nakai, T. Vreven, J. A. Montgomery, Jr., J. E. Peralta, F. Ogliaro, M. Bearpark, J. J. Heyd, E. Brothers, K. N. Kudin, V. N. Staroverov, R. Kobayashi, J. Normand, K. Raghavachari, A. Rendell, J. C. Burant, S. S. Iyengar, J. Tomasi, M. Cossi, N. Rega, J. M. Millam, M. Klene, J. E. Knox, J. B. Cross, V. Bakken, C. Adamo, J. Jaramillo, R. Gomperts, R. E. Stratmann, O. Yazyev, A. J. Austin, R. Cammi, C. Pomelli, J. W. Ochterski, R. L. Martin, K. Morokuma, V. G. Zakrzewski, G. A. Voth, P. Salvador, J. J. Dannenberg, S. Dapprich, A. D. Daniels, O. Farkas, J. B. Foresman, J. V. Ortiz, J. Cioslowski, and D. J. Fox, Gaussian, Inc., Wallingford CT, 2009.

[2] Hehre, W. J.; Radom, L.; Schleyer, P. v. R.; Pople, J. A. in *Ab Initio Molecular Orbital Theory*, Wiley, New York, **1986**, pp 71-82, and references cited therein.

[3] (a) Parr, R. G.; Yang, W. *Density-Functional Theory of Atoms and Molecules*, Oxford University Press, New York, 1989. (b) Bartolotti, L. J.; Fluchichk, K. in *Reviews in Computational Chemistry*, Lipkowitz, K. B.; Boyds, D. B. Eds. VCH Publishers, New York, **1996**, Vol. 7; pp 187-216. (c) Kohn, W.; Becke, A. D.; Parr, R. G. *J. Phys. Chem*. **1996**, *100*, 12974–12980. (d) Ziegler, T. *Chem. Rev*. **1991**, *91*, 651–667.

[4] (a) Fukui, K. *J. Phys. Chem*. **1970**, *74*, 4161-4162. (b) Fukui, K. *Acc. Chem. Res*. **1981**, *14*, 363-368.

[5] (a) Gonzalez, C.; Schlegel, H. B. *J. Phys. Chem.* **1990**, *94*, 5523-5527. (b) Gonzalez, C.; Schlegel, H. B. *J. Chem. Phys*. **1991**, *95*, 5853-5860.

[6] (a) Tomasi, J.; Persico, M. *Chem. Rev*. **1994**, *94*, 2027–2094; (b) Simkin, B. Y.; Sheikhet, I. *Quantum Chemical and Statistical Theory of Solutions: A Computational Approach*; Ellis Horwood: London, UK, **1995**, pp 78–101.

[7] (a) Miertus, S.; Scrocco, E.; Tomasi, J. *J. Chem. Phys*. **1981**, *55*, 117–129; (b) Cammi, R.; Tomasi, J. *J. Chem. Phys.* **1994**, *100*, 7495–7502; (c) Barone, V.; Cossi, M.; Tomasi, J. *J. Chem. Phys*. **1997**, *107*, 3210–3221.

Table S1. Electronic energies (hartrees) of the stationary points found in the conversions **3a** → **5a** + **6a** and **5a** → **27a** → **6a** calculated at the B3LYP/6-31+G** and PCM-B3LYP/6-31+G** (using dimethylsulfoxide as solvent) theoretical levels. The ZPVE corrections calculated at each level of calculation have been included and are not scaled.

3a → 5a + 6a<br>5a → 27a → 6a	B3LYP/6-31+G**	PCM-B3LYP/6-31+G**
**3a**	-730,19741	-730,18543
**TS1-A**	-730,12172	-730,10998
**9a**	-730,15407	-730,14395
**TS2**	-730,12292	-730,11360
**10a**	-730,16904	-730,15961
**TS3**	-730,12385	-730,11585
**10a'**	-730.16757	-730,15848
**TS4**	-730,15646	-730,14747
**TS5**	-730,12358	-730,11604
**11a**	-730,16464	-730,15593
**TS1-B**	-730,13145	-730,12184
**TS1-C**	-730,09494	-730,08414
**TS6**	-730,14252	-730,13296
**13a**	-730,22680	-730,21490
**TS7**	-730,14187	-730,13172
**12a**	-730,21712	-730,20582
**TS8**	-730,16042	-730,15631
**5a**	-730,24734	-730,23610
**TS9**	-730,15217	-730,14640
**6a**	-730,24639	-730,23467
**TS10**	-730,17849	-730,16872
**TS11**	-730,17843	-730,16925
**27a**	-730,19966	-730,18837

Table S2. Relative electronic (*E*) and Gibbs (*G*) energies in kcal·mol^{-1} of the stationary points found in the conversions **3a** → **5a** + **6a** and **5a** → **27a** → **6a** calculated at B3LYP/6-31+G** and PCM-B3LYP/6-31+G** (using dimethylsulfoxide as solvent) theoretical levels. The ZPVE corrections calculated at each level of calculation have been included and are not scaled.

3a → 5a + 6a<br>5a → 27a → 6a	*E*	*G*	$E_{(DMSO)}$	$G_{(DMSO)}$
**3a**	0.0	0.0	0.0	0.0
**TS1-A**	47.5	49.5	47.3	49.3
**9a**	27.2	27.9	26.0	26.9
**TS2**	46.7	47.5	45.1	46.2
**10a**	17.8	18.8	16.2	17.7
**TS3**	46.2	47.5	43.7	45.1
**10a'**	18.7	19.4	16.9	18.3
**TS4**	25.7	27.4	23.8	25.6
**TS5**	46.3	47.2	43.5	44.8
**11a**	20.6	21.5	18.5	18.9
**TS1-B**	41.4	43.5	39.9	42.2
**TS1-C**	64.3	66.4	63.6	65.9
**TS6**	34.4	36.0	32.9	34.8
**13a**	-18.4	-16.0	-18.5	-15.8
**TS7**	34.8	36.3	33.7	35.7
**12a**	-12.4	-10.0	-12.8	-10.7
**TS8**	23.2	25.8	18.3	20.9
**5a**	-31.3	-30.9	-31.8	-30.8
**TS9**	28.4	31.0	24.5	26.9
**6a**	-30.7	-30.2	-30.9	-29.8
**TS10**	11.9	12.3	10.5	11.9
**TS11**	11.9	12.4	10.2	11.3
**27a**	-1.4	-1.3	-1.8	-0.9

Cartesian coordinates and geometries of the stationary points found in the transformations **3a** → **5a** + **6a** and **5a** → **27a** → **6a** optimized at the B3LYP/6-31+G** theoretical level.

<table>
<tr>
<td rowspan="2">C,-1.740434,-0.631278,0.377553
C,-1.12297,0.721521,0.097166
C,0.242804,0.809986,-0.269938
C,1.055539,-0.403656,-0.43976
C,2.365788,-0.574187,-0.117599
C,3.130538,-1.799349,-0.409547
C,4.395964,-1.63001,0.054543
C,4.492665,-0.305013,0.684997
C,3.293256,0.328808,0.583864
C,0.785954,2.08377,-0.524534
C,0.013752,3.237314,-0.394702
C,-1.324334,3.138533,-0.009685
C,-1.886536,1.882355,0.231944
H,-1.09364,-1.22506,1.045999
H,0.540199,-1.257167,-0.876093
H,2.726751,-2.659079,-0.930002
H,5.209281,-2.342938,-0.013471
H,5.381493,0.083821,1.168704
H,3.036743,1.29812,0.987649
H,1.814087,2.156563,-0.861847
H,0.454557,4.20777,-0.603625
H,-1.932408,4.032224,0.095721
H,-2.923045,1.794201,0.536219
O,-1.922569,-1.374884,-0.84126
O,-3.021893,-0.513891,0.954365
C,-3.664606,-1.756936,0.673999
C,-3.160095,-2.094375,-0.738577
H,-3.356765,-2.517082,1.406507
H,-4.743682,-1.605482,0.739648
H,-2.981366,-3.165853,-0.879958
H,-3.840223,-1.73473,-1.518642</td>
<td>**3a**
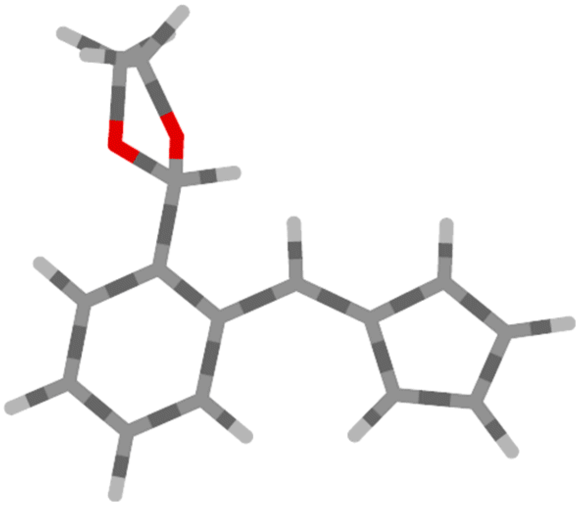</td>
</tr>
<tr>
<td>Freq: 30.5805
Sum of electronic and zero-point energies=-730.197405
Sum of electronic and thermal energies= -730.183279
Sum of electronic and thermal enthalpies= -730.182335
Sum of electronic and thermal Free energies= -730.240516</td>
</tr>
</table>

TS1-A

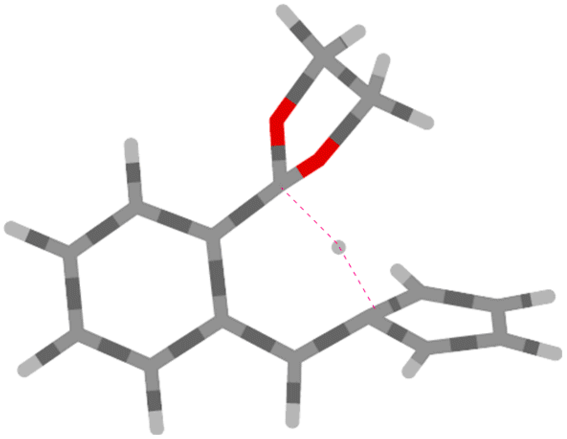

C,-0.2153356272,1.0570295615,0.1002966886
C,-1.3580063907,0.1926753112,-0.0016138736
C,-1.197999761,-1.2216044914,-0.2702944931
C,0.0274418064,-1.8652618213,-0.0463167735
C,1.2683524507,-1.1633564604,0.2031393049
C,2.2385573346,-1.6962264016,1.2027935857
C,3.5007942998,-1.5363311795,0.7292347032
C,3.4407456839,-0.9854975537,-0.6267321179
C,2.1455913642,-0.7531827935,-0.9561238231
C,-2.3989519087,-1.9981451108,-0.4267802881
C,-3.6399516496,-1.4553851277,-0.2142281211
C,-3.769373025,-0.1001248808,0.1965952965
C,-2.6461766438,0.6862464364,0.3356262137
H,0.7661582009,0.0042449218,0.5540566201
H,-0.0128623659,-2.9272137415,0.1841477945
H,1.9477245398,-2.0873833122,2.1704872778
H,4.416837137,-1.7875185707,1.253261223
H,4.2998344079,-0.8256014464,-1.2688266612
H,1.757719452,-0.394055302,-1.8977314436
H,-2.296431871,-3.0493300018,-0.6819203973
H,-4.528816606,-2.06812942,-0.3356853868
H,-4.7542195344,0.3231097309,0.3699454117
H,-2.7348326599,1.7256284117,0.6342494467
O,-0.286156528,2.1308168427,0.9788209401
O,0.4323649253,1.4729826609,-1.0538727034
C,1.3175494043,2.5261733674,-0.6491419038
C,0.4988652184,3.2139359081,0.444432905
H,2.2524703901,2.0975833585,-0.2655651728
H,1.5184452567,3.1497963928,-1.5212466813
H,1.1017504117,3.6378840782,1.2505554216
H,-0.1756927127,3.9777596331,0.0406470076

Freq: -1202.870
Sum of electronic and zero-point energies=
-730.121717
Sum of electronic and thermal energies=
-730.108449
Sum of electronic and thermal enthalpies=
-730.107505
Sum of electronic and thermal Free energies=
-730.161590

C,2.3599936349,2.4401253609,-0.3171201636
C,2.83372813,1.1921978337,-0.5910653403
C,2.1239105297,0.1963398175,-1.4370157896
C,1.3948851941,0.43286818,-2.5642811499
C,1.2375660056,1.7100180978,-3.3493649434
C,1.5682696314,1.5378293573,-4.8207729707
C,0.5097046641,1.9088762773,-5.5730858745
C,-0.5884197276,2.3287885069,-4.6894106199
C,-0.1894558463,2.2248502241,-3.4027009976
C,2.3467039429,-1.1978475921,-1.0438539438
C,3.3837656888,-1.5649563809,-0.2505008893
C,4.2938917016,-0.5714874651,0.281643523
C,4.0297183673,0.7496769049,0.1205861885
H,1.8958150909,2.4840338795,-2.9310467351
H,0.9145130017,-0.4300327332,-3.0235540597
H,2.5260381463,1.1720149711,-5.1719426149
H,0.4605932322,1.9055567777,-6.6569516753
H,-1.5614765126,2.6630530912,-5.0340280076
H,-0.7699756056,2.4358744482,-2.5140211425
H,1.7025823098,-1.9515680622,-1.4899578775
H,3.5568717223,-2.6151332193,-0.0321219227
H,5.1680786035,-0.8904758896,0.8412814456
H,4.6674790586,1.5014047536,0.5730313402
O,3.056103185,3.360046191,0.4156413925
O,1.1330385698,2.9215253266,-0.6537834246
C,1.0839730644,4.3158966065,-0.2986233342
C,2.1424053054,4.4038321775,0.7972571739
H,1.3379711148,4.9199960421,-1.1777810337
H,0.071038016,4.5465274804,0.0338005289
H,2.6911044835,5.3463976081,0.8189351062
H,1.7334545978,4.1821118285,1.7900416117

9a

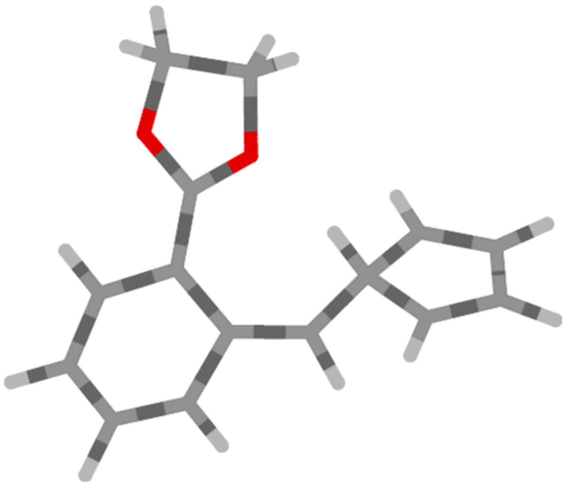

Freq: 51.6599
Sum of electronic and zero-point energies=
-730.154072
Sum of electronic and thermal energies=
-730.139871
Sum of electronic and thermal enthalpies=
-730.138926
Sum of electronic and thermal Free energies=
-730.196025

C,0.2261852839,2.0577941621,0.1137266532
C,-0.4899980372,1.2808353063,0.9807697072
C,-0.5889161146,-0.193395584,0.872388741
C,0.3621941869,-1.0669778196,0.408103296
C,1.7852686823,-0.8441817447,0.1397522698
C,2.7307359096,-0.0394933098,0.854865028
C,4.0212078223,-0.4096496185,0.4780565613
C,3.9677997971,-1.4382397219,-0.4868868702
C,2.6148325796,-1.7383036975,-0.7236798374
C,-1.8275006858,-0.7569221277,1.400049374
C,-2.6577170729,-0.0527215717,2.2128598208
C,-2.362467298,1.3188419892,2.560096459
C,-1.3241699209,1.9597209792,1.964030904
H,1.9997618947,-0.5949569696,-1.1026357142
H,0.0426614477,-2.1043732049,0.3161581031
H,2.4637345469,0.7196198366,1.5783487628
H,4.9317573545,0.0580558541,0.8373054564
H,4.8086921112,-1.881073173,-1.0046782114
H,2.1964205833,-2.5307020252,-1.3325833976
H,-2.026055025,-1.8056081592,1.1925644726
H,-3.5394292276,-0.5295150334,2.6320577377
H,-2.9948759962,1.8419219945,3.2708772603
H,-1.1310121925,3.0077632371,2.1661705966
O,0.3869060386,3.4014154151,0.2767414552
O,0.8318535628,1.6304025564,-1.0186624078
C,1.6157943831,2.7197184303,-1.5511534681
C,0.9148814325,3.9354042521,-0.9520538318
H,2.6487776854,2.6119066817,-1.2042095119
H,1.573101933,2.6655338532,-2.6396064468
H,1.5810338351,4.7626444636,-0.7043795068
H,0.0859143002,4.2904022494,-1.5751541553

TS2

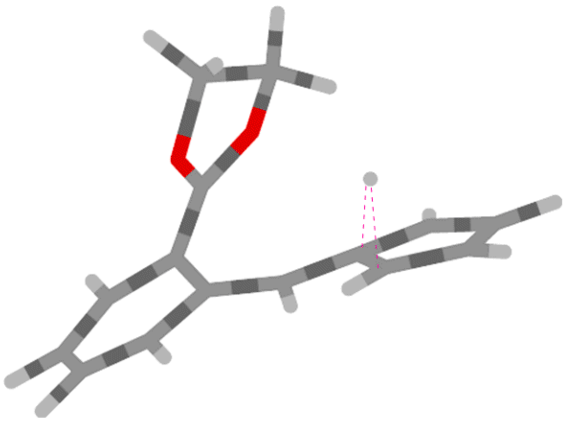

Freq: -1173.3070
Sum of electronic and zero-point energies=
-730.122915
Sum of electronic and thermal energies=
-730.108994
Sum of electronic and thermal enthalpies=
-730.108050
Sum of electronic and thermal Free energies=
-730.164782

C,-0.3842096184,1.3774335516,-0.2129167617
C,-1.1076757092,0.5557642607,0.6118946663
C,-1.1841183568,-0.9080639938,0.4415887207
C,-0.2182757836,-1.7762800829,-0.0490883394
C,1.1934339528,-1.6624897757,-0.2595567274
C,2.1131450724,-0.687466759,0.0580118525
C,3.4562440986,-1.146823567,-0.2564874181
C,3.4077173938,-2.3919783485,-0.7923871905
C,1.9723154753,-2.8343189927,-0.8450636253
C,-2.4187164021,-1.5057955381,0.9177990855
C,-3.2953509923,-0.8449243372,1.7252069713
C,-3.0417733644,0.5176766464,2.117060362
C,-1.9915663981,1.1921277443,1.577941766
H,1.6465459291,-3.0498125427,-1.8737805542
H,-0.5899038497,-2.7894572326,-0.2056588433
H,1.8958973079,0.262205561,0.528956584
H,4.3559338851,-0.5649009523,-0.0814596917
H,4.2453431745,-2.9875327162,-1.1349731522
H,1.8040012916,-3.7572058813,-0.2711904017
H,-2.5876208478,-2.5533344452,0.6801529776
H,-4.1756739621,-1.3562204795,2.1043151688
H,-3.7087325234,1.011632752,2.8172135652
H,-1.8253960625,2.2373244646,1.8150075129
O,-0.279144818,2.7238974133,-0.0222065983
O,0.2729916003,0.9874146579,-1.3235247056
C,0.9712104704,2.1265895388,-1.8687290576
C,0.2134714386,3.2980866088,-1.24742023
H,2.0182926051,2.0818548279,-1.5529675093
H,0.9056566623,2.0737924394,-2.9562179378
H,0.8388445195,4.1550247311,-0.9943274868
H,-0.6365021888,3.6194254469,-1.8605150019

10a

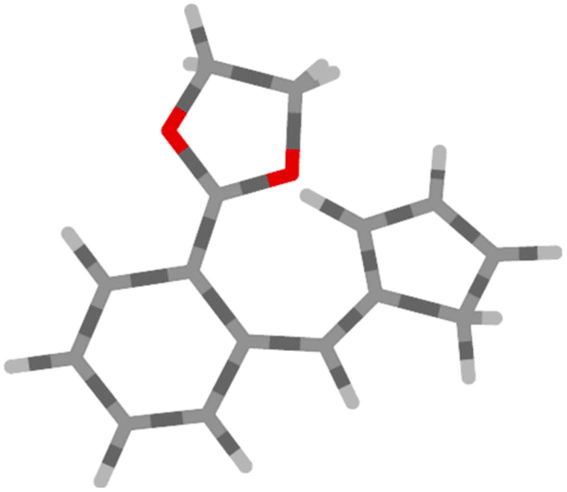

Freq: 49.6524
Sum of electronic and zero-point energies=
-730.169037
Sum of electronic and thermal energies=
-730.154889
Sum of electronic and thermal enthalpies=
-730.153945
Sum of electronic and thermal Free energies=
-730.210553

C,-0.7054466711,1.1791710151,0.0379104679
C,-1.2840305633,-0.0418555563,0.2259629765
C,-0.7712063742,-1.2929289069,-0.379838961
C,0.5343317901,-1.6493670576,-0.6179789082
C,1.806926986,-1.1099283134,-0.153640917
C,2.0835744945,-0.2597071778,1.0448732116
C,3.4656506971,-0.3028306927,1.2875118845
C,4.0663039867,-1.1287193819,0.3118003743
C,3.0909709089,-1.6151858266,-0.552128682
C,-1.8009072542,-2.278678537,-0.6896332551
C,-3.0496368361,-2.2170705162,-0.1566899964
C,-3.4204729302,-1.1306408576,0.7212959879
C,-2.5748206572,-0.084582318,0.905292258
H,1.8867557077,0.1708930698,-0.2282517583
H,0.6627481102,-2.5665171624,-1.1895177784
H,1.3095458367,0.1864394082,1.654258669
H,3.9775987761,0.2484261585,2.0663163634
H,5.1313244695,-1.3090193273,0.2184676557
H,3.2476018547,-2.2616198304,-1.4069820992
H,-1.5077368496,-3.1328081065,-1.2952140673
H,-3.7675952228,-3.0061989744,-0.3610359438
H,-4.3918459622,-1.1401401371,1.206572549
H,-2.8677936841,0.7666640798,1.5103328843
O,-1.1443207739,2.3256996183,0.6335597074
O,0.3455069586,1.4342921814,-0.789377366
C,0.7023266131,2.8283622729,-0.6545676608
C,-0.5795219741,3.4344554887,-0.0894650245
H,1.5487550511,2.9112051341,0.035765603
H,0.9813333492,3.2010996556,-1.6408526117
H,-0.4171199821,4.2520730601,0.6137248203
H,-1.2762899549,3.7458574375,-0.8765305829

TS3

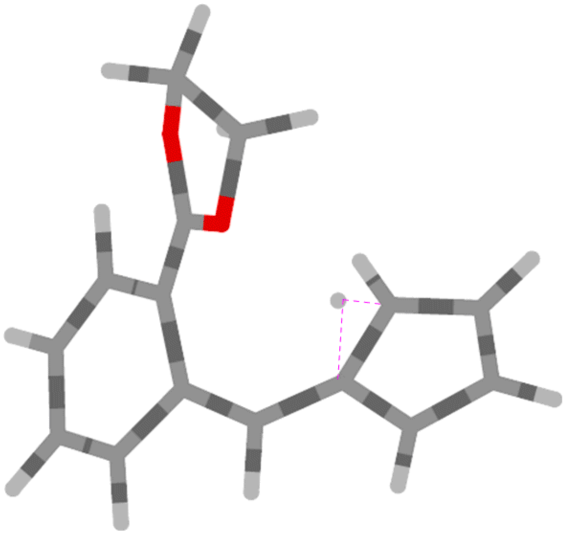

Freq: -1161.2006
Sum of electronic and zero-point energies=
-730.123846
Sum of electronic and thermal energies=
-730.110114
Sum of electronic and thermal enthalpies=
-730.109169
Sum of electronic and thermal Free energies=
-730.164763

C,0.7553278176,1.2296313723,0.0319872027<br>C,1.2834189423,0.0025179749,-0.2518763047<br>C,0.8051938833,-1.2518668141,0.369218696<br>C,-0.4921056809,-1.6252843205,0.6686352846<br>C,-1.7731853334,-1.1717736644,0.2096677124<br>C,-2.9653420109,-1.7476886451,0.5911268355<br>C,-4.0634578341,-1.2016679053,-0.1845635225<br>C,-3.5839806204,-0.2877355075,-1.0648260735<br>C,-2.0959440057,-0.1579096602,-0.8744517252<br>C,1.8511076321,-2.2297506858,0.6215859393<br>C,3.0694261193,-2.1673459198,0.0158122217<br>C,3.3840135994,-1.0840574097,-0.8846622546<br>C,2.5278493413,-0.0369012489,-1.0129624079<br>H,-1.5245883771,-0.3442355835,-1.7940104709<br>H,-0.5621766617,-2.5303332977,1.2729327017<br>H,-3.066152053,-2.5261076061,1.3405727106<br>H,-5.1016706601,-1.4953242781,-0.0705805608<br>H,-4.1569699805,0.2942313051,-1.7771623699<br>H,-1.8456908306,0.8582305974,-0.5528057169<br>H,1.594428318,-3.088197753,1.2379406891<br>H,3.7978090351,-2.9569944316,0.1766992747<br>H,4.321858987,-1.0943288261,-1.4320716398<br>H,2.7827503963,0.8161331409,-1.6325532578<br>O,1.2104162418,2.3879099708,-0.5272156365<br>O,-0.2281133074,1.480128199,0.9334440651<br>C,-0.4891464222,2.8975387037,0.9494137335<br>C,0.7726465702,3.4738220656,0.3096297723<br>H,-1.3904171742,3.1005182148,0.3595246824<br>H,-0.647267695,3.2024090219,1.9849461864<br>H,0.5983322208,4.3438582992,-0.3245073638<br>H,1.5511755423,3.6960646917,1.048849597	**10a'**<br>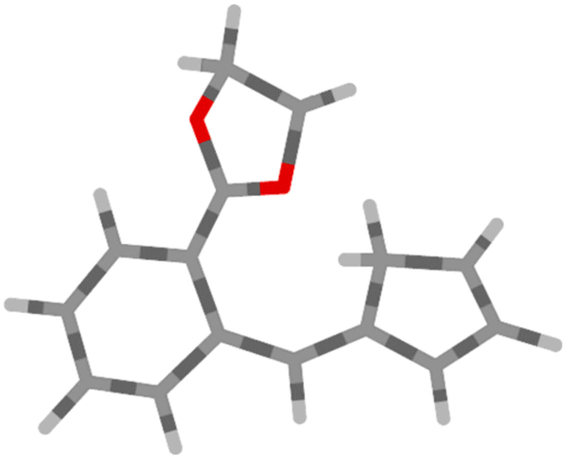<br><br>Freq: 36.4026<br>Sum of electronic and zero-point energies=<br>-730.167567<br>Sum of electronic and thermal energies=<br>-730.153309<br>Sum of electronic and thermal enthalpies=<br>-730.152365<br>Sum of electronic and thermal Free energies=<br>-730.209564

C,-0.8983133394,1.1848077637,0.0094666893
C,-1.2060126907,-0.1290027439,0.2246856745
C,-0.6248428435,-1.2393281448,-0.5563654736
C,0.6908317879,-1.3968992352,-0.9312551266
C,1.9134001267,-0.8805819692,-0.3353097836
C,2.8909554199,-1.8720704983,0.2912453036
C,3.9509713427,-1.0030508613,0.9088334928
C,3.6270385035,0.2929236691,0.6976559325
C,2.3720019414,0.3735533258,-0.0479555665
C,-1.5553102747,-2.3242948263,-0.8399189547
C,-2.6890663197,-2.5015981846,-0.1081509793
C,-3.039209712,-1.5764975369,0.9444992408
C,-2.3267184157,-0.4298618818,1.1064286772
H,2.3751517803,-2.4877041799,1.0416027818
H,0.8618684469,-2.312518024,-1.502783334
H,3.3043931732,-2.5742182735,-0.448646811
H,4.8223053948,-1.3815624965,1.4300670868
H,4.2004218374,1.1551135332,1.0231005662
H,1.911378268,1.2954073632,-0.3650539228
H,-1.2569496848,-3.065448779,-1.577522896
H,-3.3207751282,-3.3670944847,-0.2880526774
H,-3.8984924546,-1.7813221007,1.5759395523
H,-2.6260737286,0.3117782941,1.8390495262
O,-1.4121694589,2.1970747685,0.764566861
O,-0.1975061722,1.6759619553,-1.0460696352
C,-0.0988264646,3.1042276309,-0.8974854716
C,-1.2807169623,3.4189305146,0.0156600356
H,0.8629616125,3.3538056429,-0.4326769634
H,-0.1619483897,3.5525092591,-1.8897532054
H,-1.1018826166,4.2326742757,0.7196510566
H,-2.2053949792,3.6007232244,-0.5444296762

TS4

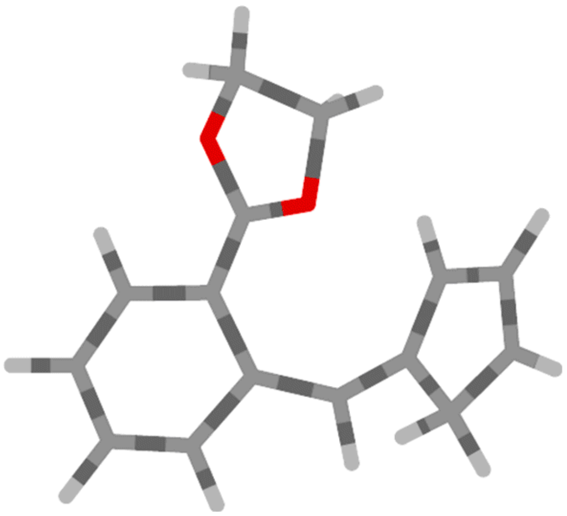

Freq: -41.2599
Sum of electronic and zero-point energies=
-730.156455
Sum of electronic and thermal energies=
-730.143037
Sum of electronic and thermal enthalpies=
-730.142093
Sum of electronic and thermal Free energies=
-730.196915

C,0.1990293086,1.2039842565,0.0241666191
C,0.7245557261,-0.0311315149,0.2673913442
C,0.1675962832,-1.2858187758,-0.2918894755
C,-1.1527101934,-1.6260404375,-0.4887835406
C,-2.3997854859,-1.0968945408,0.0158841516
C,-2.6369303541,-0.1477976686,1.0451305281
C,-4.1085457607,-0.0443719919,1.2307506136
C,-4.6993094182,-0.9471545241,0.3084958428
C,-3.6762281274,-1.5696192061,-0.4031143763
C,1.1689300644,-2.2968159932,-0.6049615751
C,2.4343274991,-2.2516710123,-0.107440777
C,2.8474168805,-1.1578637799,0.7414039518
C,2.0277627071,-0.0904992819,0.922699839
H,-1.9089635252,0.3164043076,1.6965784797
H,-1.2937909144,-2.5509965052,-1.0482172463
H,-3.413133104,0.8192342732,0.5671200726
H,-4.579930903,0.4748539289,2.0559975784
H,-5.762978903,-1.0847382231,0.164260461
H,-3.8201583302,-2.2811121405,-1.2095429223
H,0.8423332212,-3.1588007614,-1.1821888429
H,3.1298853477,-3.0601504833,-0.3137675125
H,3.8272510811,-1.1808018071,1.2092620508
H,2.3518015133,0.7653014975,1.5054500745
O,-0.9278528357,1.4663827786,-0.6825083269
O,0.7971793331,2.3649717746,0.4405856351
C,-0.0614285336,3.4624183785,0.0854188401
C,-0.9073353402,2.8634625474,-1.0349554097
H,-0.6624631455,3.7497536169,0.9564956723
H,0.5645262348,4.3002063791,-0.2263545491
H,-1.9373231457,3.2216048687,-1.0662942525
H,-0.4402397799,2.9779073399,-2.019967448

TS5

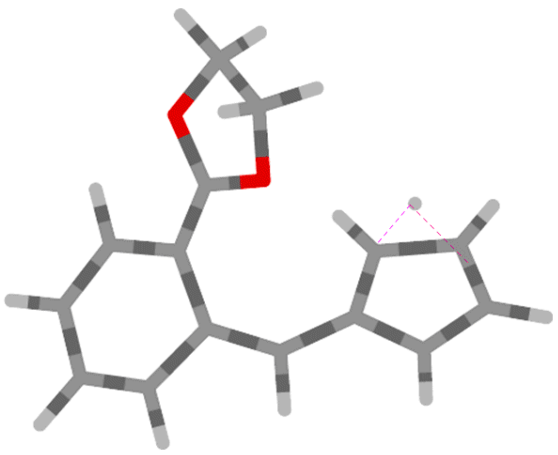

Freq: -1261.6447
Sum of electronic and zero-point energies=
-730.123580
Sum of electronic and thermal energies=
-730.109626
Sum of electronic and thermal enthalpies=
-730.108682
Sum of electronic and thermal Free energies=
-730.165325

C,0.63178139,1.3225239254,0.0527224986
C,1.3832254962,0.1870270371,0.1960214548
C,1.0365489293,-1.1044355272,-0.4328420226
C,-0.2141071514,-1.639109431,-0.67553937
C,-1.5361137716,-1.3488662005,-0.1650480758
C,-2.7148204395,-2.0836715593,-0.6857958461
C,-3.8255504162,-1.7440000571,-0.003037138
C,-3.4583406614,-0.7389177373,1.0570666237
C,-1.9803724505,-0.5473843418,0.8531519737
C,2.1853787712,-1.9273289813,-0.7812775088
C,3.4321022525,-1.6961699326,-0.2857710483
C,3.6761974713,-0.5788383607,0.5923494541
C,2.6910818656,0.3291863094,0.8200884436
H,-4.0266363443,0.1991245528,0.9491331328
H,-0.1911529162,-2.5417021124,-1.2863225185
H,-2.65881132,-2.7918184477,-1.5071147922
H,-4.829614085,-2.1170914278,-0.1680470199
H,-3.6893668159,-1.1105837824,2.068363928
H,-1.3748048562,0.0944798943,1.4781979665
H,1.9987086216,-2.8088777525,-1.389736497
H,4.2452682802,-2.3760165826,-0.524186109
H,4.6555601146,-0.4504331423,1.0432688876
H,2.8786209623,1.2097431776,1.4251477602
O,-0.5305294781,1.4141899234,-0.6208235807
O,1.0116486842,2.5342337154,0.557033988
C,-0.0659012328,3.4648287378,0.3399601066
C,-0.8370493022,2.8109498552,-0.8043805448
H,-0.6610135633,3.5450770265,1.2567165056
H,0.3662188383,4.4350253132,0.090056563
H,-1.9195704858,2.928813979,-0.745836537
H,-0.4762033869,3.1252969292,-1.7901386782

11a

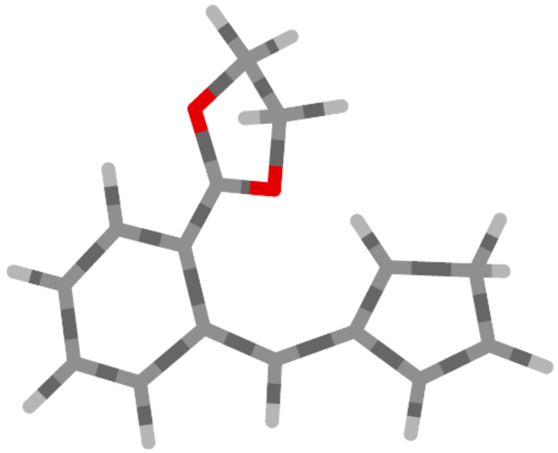

Freq: 47.1729
Sum of electronic and zero-point energies=
-730.164637
Sum of electronic and thermal energies=
-730.150450
Sum of electronic and thermal enthalpies=
-730.149505
Sum of electronic and thermal Free energies=
-730.206328

C,-0.5174123413,0.0088909056,2.3964906379
C,-0.7941736041,0.6583888738,1.1349951643
C,0.173051553,0.7491560142,0.0699327932
C,1.2911264551,-0.1145008962,-0.1599881705
C,1.7027518637,-1.2790791597,0.475654071
C,1.3876056578,-1.7316456562,1.8807071421
C,1.7027727005,-3.1250000883,1.9156447767
C,2.2149663899,-3.5028199148,0.6626281747
C,2.2728312807,-2.3936850871,-0.1937812685
C,-0.1569387303,1.6157983387,-1.0211910028
C,-1.3797666643,2.238214621,-1.1330266818
C,-2.3713544816,2.0092518243,-0.1526554919
C,-2.0836924931,1.2201105135,0.9430358693
H,0.1886010065,-1.2610567618,2.0534244361
H,1.7454194467,0.0361063543,-1.139050422
H,1.7993346481,-1.1032196984,2.6748409136
H,1.6693639658,-3.7505846216,2.7994487611
H,2.532293857,-4.5062531738,0.3967507118
H,2.5369972105,-2.4146585649,-1.2447803996
H,0.5780000699,1.7329625669,-1.812975256
H,-1.5906673259,2.8754799948,-1.9866513155
H,-3.3461412282,2.4801801063,-0.2396986928
H,-2.8255704635,1.0695742927,1.7185914477
O,-1.6043919833,-0.3164569277,3.2064259855
O,0.4778044847,0.5857882976,3.1925018887
C,0.2238925993,0.1562994652,4.5372395525
C,-1.2995531336,0.066292218,4.557804166
H,0.6914890499,-0.8197993726,4.7236694962
H,0.6428255999,0.9046725962,5.2116686683
H,-1.6940462348,-0.6969164572,5.2317473394
H,-1.7641614552,1.0357638975,4.7762272053

TS1-B

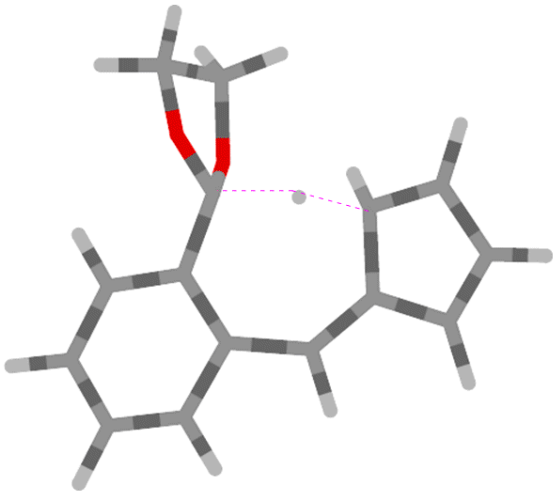

Freq: -1368.8674
Sum of electronic and zero-point energies=
-730.131449
Sum of electronic and thermal energies=
-730.118349
Sum of electronic and thermal enthalpies=
-730.117405
Sum of electronic and thermal Free energies=
-730.171194

C,-2.0556279963,-0.1302475727,-0.8323151399<br>C,-1.3204842942,1.0336095024,-0.3970066445<br>C,0.1081854573,1.2274938596,-0.6286874867<br>C,1.0253098079,0.1557877698,-0.8173415134<br>C,0.7819736674,-1.0616797659,-0.1401038239<br>C,0.0466936787,-1.1037560086,1.0489546436<br>C,-0.9190025127,-2.1460877213,0.8793561516<br>C,-0.402145727,-3.0097035162,-0.2248458698<br>C,0.6437575757,-2.3715385049,-0.8095283637<br>C,0.5844778759,2.5767031839,-0.6204127909<br>C,-0.1927893346,3.6162740701,-0.1652816314<br>C,-1.5173558537,3.3694777172,0.2798700822<br>C,-2.0649989057,2.1095892981,0.1658975783<br>H,-1.7244539353,-1.2944385528,0.2236584716<br>H,1.8721433107,0.2930159519,-1.489653147<br>H,-0.0791369063,-0.2566224992,1.7108360018<br>H,-1.5276998899,-2.5592815763,1.6834602651<br>H,-0.8455445209,-3.9503770015,-0.53002001<br>H,1.1716086689,-2.6721573543,-1.7072846465<br>H,1.6252196979,2.7479326346,-0.8827834315<br>H,0.2187301057,4.6199611074,-0.1094149493<br>H,-2.1205775614,4.1869429501,0.6634973458<br>H,-3.1041333929,1.9440906088,0.4253962398<br>O,-1.6398344965,-0.7779491359,-1.9899794388<br>O,-3.4452035948,-0.0214404947,-0.854256771<br>C,-3.9094436023,-1.155468956,-1.5911961351<br>C,-2.7858097952,-1.3813060459,-2.6109715848<br>H,-4.0279805463,-2.0137509408,-0.9165435392<br>H,-4.8717464043,-0.8991306772,-2.0371524026<br>H,-2.5759158679,-2.43907095,-2.7962764072<br>H,-2.9722361081,-0.8705545798,-3.5621095523	**TS1-C**<br>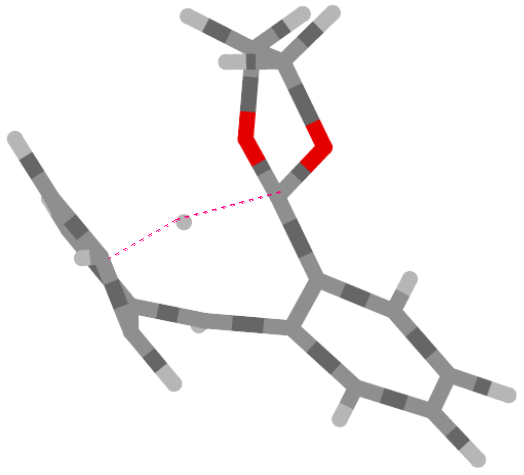<br>Freq: -1454.4433<br>Sum of electronic and zero-point energies=<br>-730.094944<br>Sum of electronic and thermal energies=<br>-730.081690<br>Sum of electronic and thermal enthalpies=<br>-730.080746<br>Sum of electronic and thermal Free energies=<br>-730.134720

C,-0.5093510587,0.8834100016,-0.3720861449
C,-0.9114484337,-0.4754458641,-0.3619692205
C,-0.3425964528,-1.4315217269,0.5549730919
C,1.0035433045,-1.4079250736,0.9920203391
C,2.0237903549,-0.6087435224,0.4503366891
C,3.3562205362,-1.1592827241,0.1328788079
C,4.0400653967,-0.3054647108,-0.6547873571
C,3.2279875505,0.9452708685,-0.8873239627
C,1.9532515518,0.6898246467,-0.1014039599
C,-1.0971520172,-2.6333544277,0.7677363161
C,-2.1792502586,-2.9591205017,-0.0146396086
C,-2.5841553028,-2.0997072202,-1.070265562
C,-1.952470812,-0.8881897004,-1.2445290328
H,3.7452308781,1.8504244887,-0.5353655911
H,1.3246112539,-2.3043177749,1.520378268
H,3.6783833042,-2.1524485512,0.430255944
H,4.9989531336,-0.4986367994,-1.1234882955
H,3.0477251859,1.100710516,-1.9632106541
H,1.5172700636,1.5005189822,0.4541650116
H,-0.7387630626,-3.3333244381,1.5182110712
H,-2.705673804,-3.8948501688,0.1519774204
H,-3.4212740406,-2.3724380651,-1.7057548621
H,-2.30030761,-0.1918617779,-1.9996167458
O,-0.5045405846,1.6096641105,0.7955192342
O,-0.8530144784,1.6801945206,-1.4241441134
C,-0.6114033713,3.0419601179,-1.0430683639
C,-0.740737586,2.99558491,0.4828546597
H,0.3941779116,3.3316600448,-1.369568726
H,-1.3563947206,3.6698763681,-1.534215491
H,-0.0008263285,3.6095604688,1.0028642845
H,-1.7452532031,3.2586148028,0.8310430536

TS6

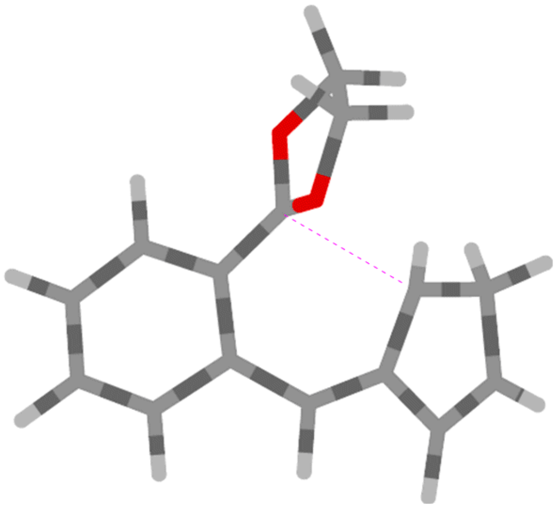

Freq: -303.5786
Sum of electronic and zero-point energies=
-730.142524
Sum of electronic and thermal energies=
-730.129170
Sum of electronic and thermal enthalpies=
-730.128225
Sum of electronic and thermal Free energies=
-730.183082

C,-0.3893995568,0.6633275763,0.3006035843
C,1.0404705697,0.1069697333,0.3273719551
C,1.2596284146,-1.2202574374,-0.1274802219
C,0.1224672933,-2.0743616561,-0.4677364112
C,-1.1245805529,-1.7054094163,-0.107743502
C,-2.4191319567,-2.3133556122,-0.3667007565
C,-3.4147175363,-1.4797786543,0.0023150037
C,-2.8919227395,-0.1633885111,0.5350147149
C,-1.3736753721,-0.4336475377,0.6901916303
C,2.5807545937,-1.6886703586,-0.2270534649
C,3.6602738536,-0.8837167285,0.1383994408
C,3.4344982445,0.407986172,0.6189127987
C,2.1253148958,0.8955872898,0.7087766067
H,-3.0778828215,0.6404173153,-0.1887714326
H,0.3082109721,-3.0013703839,-1.0048641066
H,-2.5485662308,-3.289859605,-0.8221790441
H,-4.4742635234,-1.6946517886,-0.0966140844
H,-3.3658755678,0.1333842327,1.4771474262
H,-1.1564582355,-0.6566939453,1.7455181011
H,2.7550906417,-2.7009020225,-0.5834386105
H,4.6731860541,-1.2684504667,0.0595228893
H,4.2682467267,1.0331973963,0.9248038322
H,1.9419771465,1.8916028631,1.0976175003
O,-0.7070371094,1.1417108697,-1.0136119749
O,-0.5104437912,1.7981118423,1.1685748964
C,-0.8149229522,2.9590690147,0.3868195221
C,-0.4203619635,2.5379324917,-1.0275166137
H,-1.8860204054,3.1889284201,0.4585069087
H,-0.2372121355,3.8041323458,0.7739735432
H,-1.0198681358,2.9994672741,-1.8156241927
H,0.6455411799,2.717545287,-1.2253539381

13a

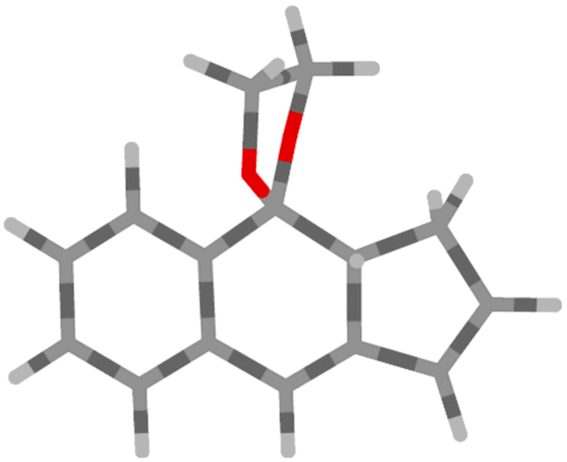

Freq: 55.0327
Sum of electronic and zero-point energies=
-730.226801
Sum of electronic and thermal energies=
-730.214029
Sum of electronic and thermal enthalpies=
-730.213085
Sum of electronic and thermal Free energies=
-730.266018

<table>
<tr>
<td rowspan="2">C,-0.3898812865,1.0328823163,0.2028197758
C,-1.1641756457,-0.1432451792,-0.0199909045
C,-0.7486198517,-1.4340174468,0.457994971
C,0.611878543,-1.8488752977,0.5615614942
C,1.7180600761,-1.156906944,0.0882462919
C,1.9024861625,0.243607394,-0.0574100734
C,3.010391201,0.4498390272,-0.9983436483
C,3.5624838181,-0.7308492309,-1.353779827
C,2.8573022598,-1.8629306338,-0.6501958868
C,-1.7531584814,-2.4516093453,0.50379695
C,-3.0010178898,-2.2718728276,-0.0503135862
C,-3.3203428298,-1.0555466872,-0.7017000947
C,-2.4098689256,-0.0189467931,-0.6966933004
H,2.4921289129,-2.6355169427,-1.3384013344
H,0.7376289166,-2.9218074461,0.7039373491
H,1.7610833625,0.9224152317,0.7649855537
H,3.3586099196,1.4258170659,-1.3229522142
H,4.4175550256,-0.8697771174,-2.0059947341
H,3.5371831405,-2.3677225879,0.055093756
H,-1.4823112136,-3.416149087,0.92566976
H,-3.7291921829,-3.0775601236,-0.018717412
H,-4.2944575008,-0.9205678291,-1.162223737
H,-2.6681052673,0.9386031818,-1.1352204061
O,-0.0708769714,1.3947091925,1.4983944962
O,-0.6543388298,2.1484401805,-0.5420607064
C,-0.0437388402,3.2676452334,0.1139699086
C,-0.03566706,2.8319710854,1.5812060376
H,0.967109025,3.411174332,-0.2861154483
H,-0.6505259552,4.152294825,-0.0856033574
H,0.8627532967,3.134928263,2.1248377021
H,-0.9251789283,3.1696291909,2.1246786252</td>
<td>TS7
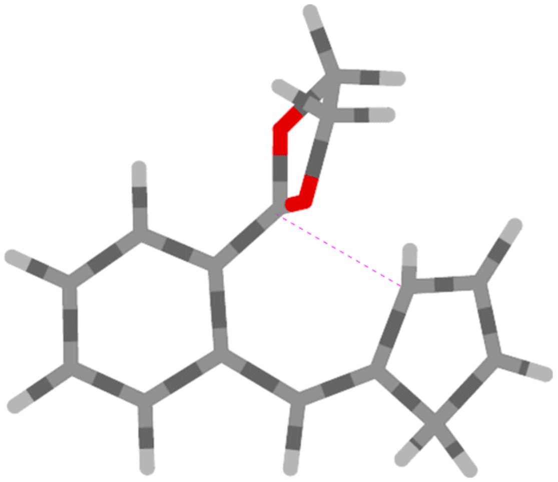</td>
</tr>
<tr>
<td>Freq: -310.5265
Sum of electronic and zero-point energies=
-730.141874
Sum of electronic and thermal energies=
-730.128512
Sum of electronic and thermal enthalpies=
-730.127568
Sum of electronic and thermal Free energies=
-730.182630</td>
</tr>
</table>

C,-0.4192124352,0.6568843835,0.356876695
C,1.0290461822,0.152855231,0.3599314235
C,1.2788741092,-1.1715737946,-0.0792255723
C,0.1526921386,-2.0591522028,-0.3975104766
C,-1.0874480676,-1.7478844746,0.005217042
C,-1.3541982278,-0.4732989254,0.7921451239
C,-2.8445183029,-0.2851233346,0.6213581134
C,-3.4146347298,-1.3563489416,0.0565853838
C,-2.406585882,-2.4333854645,-0.2946038127
C,2.6081112836,-1.6052508422,-0.1968567263
C,3.6713583665,-0.7638177197,0.1368590579
C,3.4169259639,0.5284786655,0.5996284087
C,2.0962998664,0.980694603,0.706134299
H,-2.557356595,-3.3349035005,0.3203744635
H,0.355919751,-2.9624058483,-0.9692610541
H,-1.1149921715,-0.6345500443,1.8582612138
H,-3.3712752665,0.5940639578,0.9766792929
H,-4.4794099817,-1.4743745491,-0.1203212545
H,-2.4758243836,-2.7578739316,-1.3411495345
H,2.8050193468,-2.6170369649,-0.5427490898
H,4.693324666,-1.1208515795,0.0458419696
H,4.2379068354,1.1819275329,0.8801746813
H,1.8910199031,1.9774396844,1.0820669628
O,-0.7870889966,1.0990391335,-0.9537044347
O,-0.564290507,1.7976797157,1.2118952553
C,-0.9438643596,2.9315175484,0.4224967824
C,-0.569960269,2.5067435704,-0.9963478413
H,-2.0219549937,3.1136586495,0.5209572391
H,-0.3947056364,3.8073908985,0.7815821532
H,-1.2147241257,2.9250046924,-1.7726869449
H,0.479511519,2.7362768515,-1.2286708194

12a

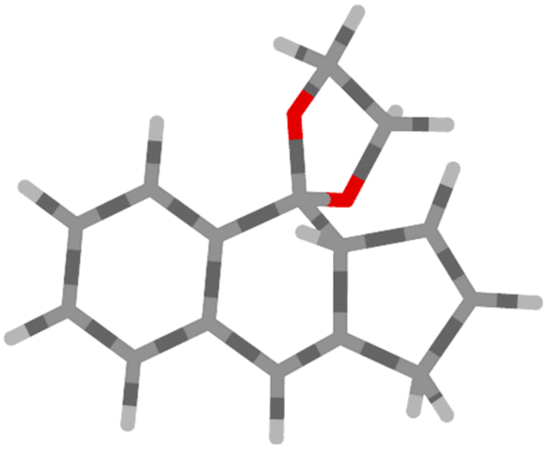

Freq: 58.6197
Sum of electronic and zero-point energies=
-730.217122
Sum of electronic and thermal energies=
-730.204253
Sum of electronic and thermal enthalpies=
-730.203309
Sum of electronic and thermal Free energies=-
730.256437

C,-0.5994834446,0.0127734248,0.2048800658<br>C,0.5926731831,-0.6002673842,0.7339070734<br>C,0.557267502,-1.9694434348,1.1512040739<br>C,-0.6725357517,-2.7172219731,1.1277414816<br>C,-1.8153228046,-2.1218607527,0.6902502909<br>C,-3.1988936611,-2.5546251131,0.8336631066<br>C,-4.0297605421,-1.5141570316,0.6211995851<br>C,-3.2772212009,-0.2481624586,0.2817654987<br>C,-1.8356695243,-0.7742727948,0.0726142968<br>C,1.7522383682,-2.5459171892,1.6384388865<br>C,2.9237402642,-1.8102535722,1.7190334145<br>C,2.9504759656,-0.4606514754,1.3141752079<br>C,1.7991432125,0.1317138547,0.8262089808<br>H,-3.6975074651,0.2445366756,-0.6021960685<br>H,-0.6839319185,-3.7199734409,1.5455638533<br>H,-3.4932491413,-3.5570596975,1.1248609524<br>H,-5.1121248634,-1.5481433379,0.6887887898<br>H,-3.3351868047,0.4756503185,1.1081296797<br>H,-1.6513777389,-0.8602920297,-1.1082281453<br>H,1.7384038755,-3.5848246052,1.956100016<br>H,3.8284844643,-2.2783201659,2.0966586161<br>H,3.8729261961,0.1082218701,1.3747865559<br>H,1.8061501118,1.1642995031,0.4975103496<br>O,-0.6113919169,-0.2889428327,-2.1199403211<br>O,-0.5426118284,1.3227111884,-0.0229615471<br>C,-1.23032594,1.8366455508,-1.200422808<br>C,-0.6961799368,1.0448385979,-2.4189346286<br>H,-2.3084963348,1.7136357643,-1.0732475038<br>H,-0.9887209634,2.9017981975,-1.2223414505<br>H,-1.3843706861,1.2813866022,-3.2567376444<br>H,0.2937504244,1.4513941416,-2.7089738583	**TS8**<br>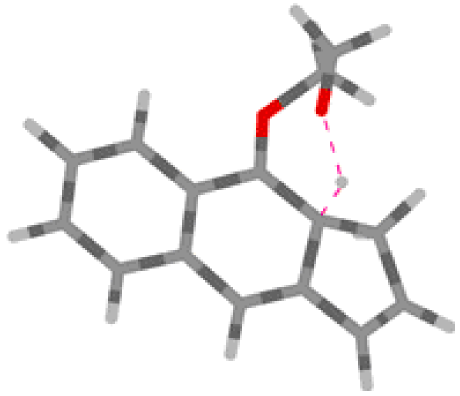<br><br>Freq: -1033.8935<br>Sum of electronic and zero-point energies=<br>-730.160420<br>Sum of electronic and thermal energies=<br>-730.147438<br>Sum of electronic and thermal enthalpies=<br>-730.146494<br>Sum of electronic and thermal Free energies=<br>-730.199341

C,0.22797,0.132669,-0.351356<br>C,-0.788108,-0.866113,-0.193639<br>C,-2.119046,-0.453843,0.162922<br>C,-2.411829,0.928233,0.335043<br>C,-1.412268,1.858842,0.151194<br>C,-1.426695,3.322239,0.230596<br>C,-0.20051,3.81032,-0.049564<br>C,0.778601,2.694699,-0.344881<br>C,-0.08047,1.459696,-0.192179<br>C,-3.116474,-1.455399,0.324515<br>C,-2.825897,-2.790243,0.138147<br>C,-1.517102,-3.189257,-0.226686<br>C,-0.522197,-2.246782,-0.389719<br>H,1.20622,2.772931,-1.354228<br>H,-3.422078,1.229918,0.59937<br>H,-2.305479,3.909324,0.474749<br>H,0.075214,4.858812,-0.067712<br>H,1.631072,2.708779,0.348457<br>H,5.495236,-1.386792,0.51294<br>H,-4.12269,-1.146373,0.596226<br>H,-3.602496,-3.538973,0.265386<br>H,-1.298202,-4.241609,-0.382872<br>H,0.47732,-2.546441,-0.685467<br>O,4.643527,-1.088268,0.853758<br>O,1.503808,-0.259346,-0.721025<br>C,2.412934,-0.471258,0.368621<br>C,3.737616,-0.9108,-0.234749<br>H,2.551395,0.453435,0.942956<br>H,2.028253,-1.243901,1.046381<br>H,4.094092,-0.140972,-0.932983<br>H,3.594308,-1.845243,-0.794658	**5a**<br>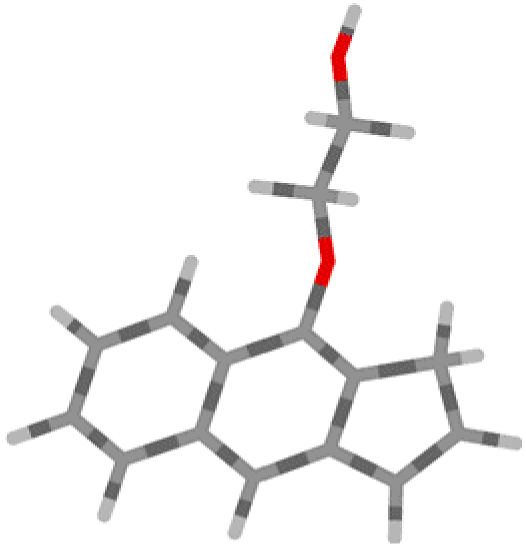<br>Freq: 29.0683<br>Sum of electronic and zero-point energies=<br>-730.247338<br>Sum of electronic and thermal energies=<br>-730.232850<br>Sum of electronic and thermal enthalpies=<br>-730.231906<br>Sum of electronic and thermal Free energies=<br>-730.289776

C,-0.0197419769,0.3335836826,-0.3201158159<br>C,1.2453842451,-0.1669662926,0.16105685<br>C,1.3308052337,-1.5128604993,0.6330733604<br>C,0.1524163567,-2.3490977044,0.7095888296<br>C,-1.0477890629,-1.8734279026,0.3100270089<br>C,-1.2078490122,-0.5385319028,-0.3095004433<br>C,-2.620329167,-0.1464480197,0.0376771153<br>C,-3.3049571978,-1.21310438,0.4783673372<br>C,-2.4286601363,-2.4482951294,0.5514567543<br>C,2.5846896644,-1.981318902,1.0789329126<br>C,3.703362758,-1.1605298801,1.0692923204<br>C,3.6099756588,0.1680620763,0.6137714956<br>C,2.3938764174,0.6553569319,0.1646883679<br>H,-2.4954589267,-2.975120436,1.5108346631<br>H,0.2451959407,-3.3280720108,1.1729503792<br>H,-1.1011367765,-0.6309076227,-1.5166240386<br>H,-3.0376524661,0.8411362063,-0.1168777484<br>H,-4.362946269,-1.2212897106,0.7170269164<br>H,-2.709397939,-3.1761329882,-0.2268864973<br>H,2.6632751688,-3.0036071265,1.4386638511<br>H,4.657494101,-1.5467179108,1.416766836<br>H,4.4892008509,0.8046631908,0.6052097727<br>H,2.3062810197,1.6725125561,-0.1991781122<br>O,-0.0919608529,-0.0871036446,-2.5388685694<br>O,-0.0913840679,1.6411670304,-0.581936908<br>C,-0.8467571282,2.0358353252,-1.762415994<br>C,-0.2752203403,1.2143237013,-2.9394563795<br>H,-1.9091061894,1.8317470824,-1.6058877<br>H,-0.6940361011,3.1136090988,-1.8514411737<br>H,-0.9890449077,1.3262729209,-3.7785070578<br>H,0.6789217027,1.6649406598,-3.2748460326	**TS9**<br>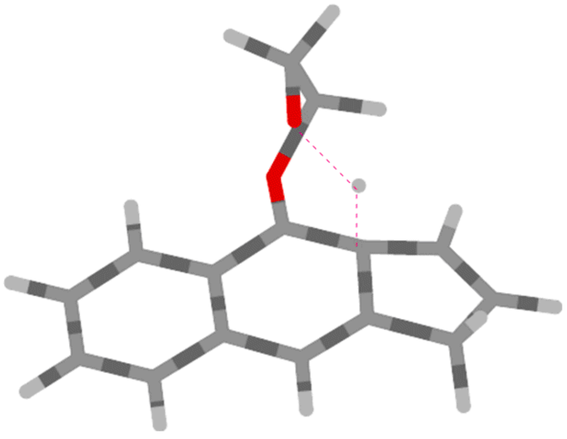
	Freq: -1159.3763<br>Sum of electronic and zero-point energies=<br>-730.152169<br>Sum of electronic and thermal energies=<br>-730.139110<br>Sum of electronic and thermal enthalpies=<br>-730.138166<br>Sum of electronic and thermal Free energies=<br>-730.191098

C,-0.3822507412,0.4182821483,0.0105846201
C,0.9129016356,-0.0858469285,0.3406836351
C,1.0148964363,-1.2720476935,1.1470270991
C,-0.1695845638,-1.9189902542,1.6108811378
C,-1.395719188,-1.3962940903,1.2914097671
C,-1.5115041727,-0.2158253915,0.4887752292
C,-2.9342408476,0.0989633444,0.3448520783
C,-3.6724928559,-0.8206406989,1.0015885716
C,-2.7913379686,-1.858526242,1.6611450154
C,2.3092290909,-1.7647839706,1.4650992018
C,3.4464628697,-1.122963466,1.0202109789
C,3.3401106304,0.0514413801,0.2353787055
C,2.1016560144,0.5607469022,-0.0969636421
H,-2.9428501941,-1.8894088589,2.7493100487
H,-0.0770070315,-2.8151617503,2.2201184502
H,1.4519196019,2.2390845278,-3.476143059
H,-3.3194198976,0.9555517298,-0.1956360265
H,-4.7545430554,-0.8312145309,1.0700159062
H,-3.0035012148,-2.8716958454,1.2911985216
H,2.3891550611,-2.6616921441,2.0743910048
H,4.4269554046,-1.5138607693,1.2767604971
H,4.2409963169,0.5577693761,-0.0995233758
H,2.0114808769,1.4699531797,-0.6822018535
O,1.0100702732,2.9170381388,-2.9480989265
O,-0.469163961,1.5696273492,-0.7497733078
C,-0.7345320846,1.3676185771,-2.1429201027
C,-0.385634452,2.6368449884,-2.9029736571
H,-0.1424161943,0.5190300815,-2.5167254219
H,-1.7979882133,1.1290033591,-2.2961843857
H,-0.8085990638,2.5642490286,-3.9168119526
H,-0.8442175115,3.4959025232,-2.4053507573

6a

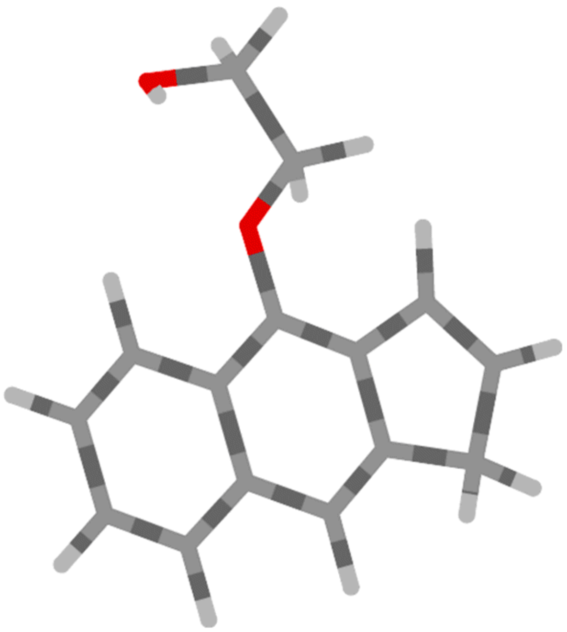

Freq: 28.0509
Sum of electronic and zero-point energies=
-730.246393
Sum of electronic and thermal energies=
-730.232093
Sum of electronic and thermal enthalpies=
-730.231149
Sum of electronic and thermal Free energies=
-730.288664

C,0.237020808,0.1029678943,-0.3360339284<br>C,-0.7783984916,-0.8521940226,-0.1936787435<br>C,-2.1271021447,-0.4155475031,0.1468859282<br>C,-2.4159638201,0.9382921117,0.3280090253<br>C,-1.3961295324,1.900033855,0.1885279833<br>C,-1.4140890222,3.308265069,0.2781289062<br>C,-0.1108797517,3.7857490711,-0.0268824517<br>C,0.7738041619,2.6132145088,-0.263847947<br>C,-0.0478445135,1.4694605291,-0.1542880856<br>C,-3.1414186399,-1.4241738438,0.2871272337<br>C,-2.8621354925,-2.7506579585,0.1023801351<br>C,-1.5388332778,-3.1714122146,-0.2437391563<br>C,-0.5339763464,-2.2527531954,-0.3864410199<br>H,0.1382716911,3.5140344627,-1.1988687986<br>H,-3.4286157692,1.2425991348,0.579192601<br>H,-2.2739530224,3.9300923154,0.4873294163<br>H,0.2629966501,4.7874938901,0.1554169703<br>H,1.8338120585,2.6704768853,-0.4663147773<br>H,5.529149883,-1.3114631602,0.5579613307<br>H,-4.1481033909,-1.1045938811,0.544315991<br>H,-3.6470437875,-3.4933449441,0.2136082467<br>H,-1.3370956244,-4.2274286997,-0.3982434125<br>H,0.4664160291,-2.5654468708,-0.6650208974<br>O,4.6788720759,-1.0013817854,0.8920070889<br>O,1.5137654605,-0.2888768977,-0.6906892191<br>C,2.431031925,-0.4535634966,0.4023868017<br>C,3.7631170964,-0.8778059896,-0.1956265731<br>H,2.5475844981,0.4871868086,0.9547403194<br>H,2.0634812715,-1.2194304685,1.0968940159<br>H,4.0977378735,-0.1203491923,-0.9181143449<br>H,3.6399082445,-1.8309845116,-0.7278841385	**TS10**<br>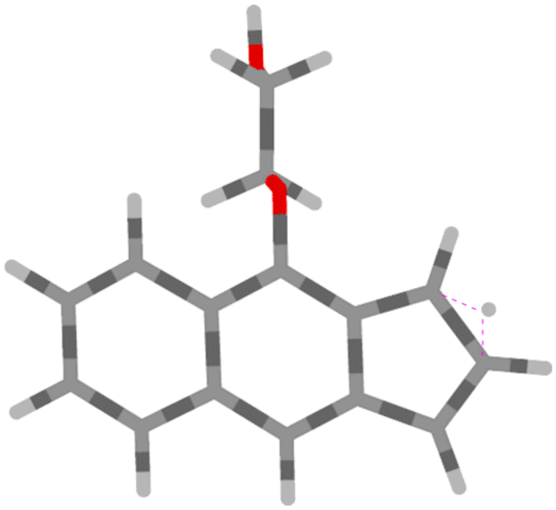<br><br>Freq: -1170.3313<br>Sum of electronic and zero-point energies=<br>-730.178492<br>Sum of electronic and thermal energies=<br>-730.164175<br>Sum of electronic and thermal enthalpies=<br>-730.163231<br>Sum of electronic and thermal Free energies=<br>-730.220837

C,0.2336317817,0.038686151,-0.3408017125
C,-0.8607713636,-0.8219870412,-0.1863244229
C,-2.1682826102,-0.2760680329,0.1568651125
C,-2.3393735793,1.1003886038,0.3170832216
C,-1.2382327277,1.9587665532,0.161530926
C,-1.1313613539,3.3662667223,0.2542940258
C,0.2738540242,3.7097269303,-0.0926077808
C,0.9868013757,2.5001031494,-0.3167299131
C,0.0767287875,1.4327135239,-0.1753480679
C,-3.2623251287,-1.1925696435,0.3174551107
C,-3.0967140749,-2.5399899367,0.1471308168
C,-1.8140090407,-3.0705764403,-0.2011814133
C,-0.7368191433,-2.2416638071,-0.3628452442
H,-0.5564904789,3.8406015619,-0.9859013086
H,-3.3196834444,1.496830332,0.5686342931
H,-1.8890926837,4.0843365837,0.537939731
H,0.7123176708,4.6859684532,0.0839727636
H,2.0245262359,2.4325133065,-0.609985766
H,5.4673900131,-1.6165644383,0.4773979505
H,-4.2366856638,-0.7861104961,0.5770403372
H,-3.9391748135,-3.2141415405,0.271782584
H,-1.7003816823,-4.1418254686,-0.3410914652
H,0.2329773685,-2.640176419,-0.6393930025
O,4.6395579866,-1.2629350575,0.8242438609
O,1.463294708,-0.4775252986,-0.7015825905
C,2.4110838214,-0.6107088419,0.3694271432
C,3.6988260309,-1.1390627293,-0.2424043751
H,2.590537855,0.3568917298,0.8527267457
H,2.0329637243,-1.3119569118,1.1244560461
H,4.0533003488,-0.4380858899,-1.010789068
H,3.5064230565,-2.1085936082,-0.7222655381

TS11

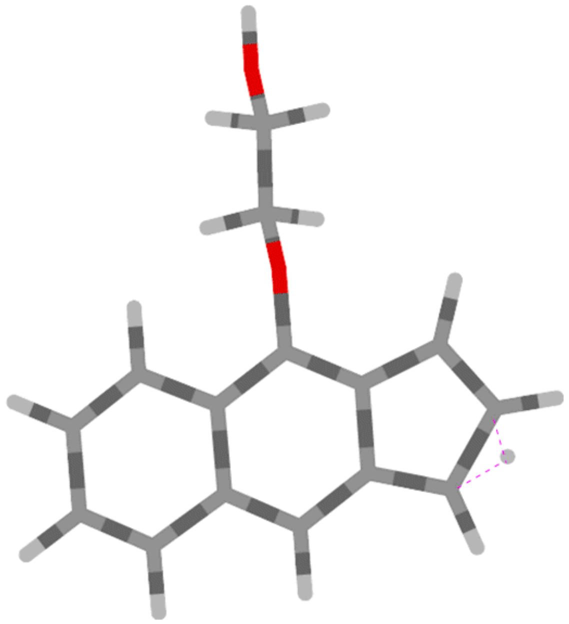

Freq: -1174.9072
Sum of electronic and zero-point energies=
-730.178430
Sum of electronic and thermal energies=
-730.164117
Sum of electronic and thermal enthalpies=
-730.163173
Sum of electronic and thermal Free energies=
-730.220808

C,0.2502218348,0.0298223216,-0.3373121936<br>C,-0.7799149544,-0.8848873721,-0.1996559786<br>C,-2.1345709,-0.4232859447,0.1599671556<br>C,-2.402296563,0.915312812,0.3591994384<br>C,-1.363321975,1.8828974553,0.1989287592<br>C,-1.363647653,3.2505134069,0.2986395495<br>C,0.0125161984,3.7554847606,-0.0087192425<br>C,0.818943851,2.5253787089,-0.2959835432<br>C,0.0026119041,1.4336156755,-0.1662922613<br>C,-3.1662247289,-1.4259976524,0.3031373662<br>C,-2.9090768208,-2.7492081065,0.1103182575<br>C,-1.5858856564,-3.193373793,-0.2500088373<br>C,-0.5684172696,-2.2995747173,-0.3987589435<br>H,0.0103410838,4.4480378258,-0.8662228909<br>H,-3.4051606763,1.2339977646,0.6307352369<br>H,-2.2074312903,3.8827776288,0.5464413514<br>H,0.4357140672,4.3333302816,0.8291792196<br>H,1.8634619238,2.5307589742,-0.5780681533<br>H,5.6236944878,-1.1363513603,0.4102403565<br>H,-4.1651831123,-1.0907271422,0.5698445509<br>H,-3.7028344453,-3.4819795101,0.2233322016<br>H,-1.4071491148,-4.2531282838,-0.4070826395<br>H,0.4247824655,-2.6291040533,-0.6824442769<br>O,4.769179393,-0.8707531956,0.7707724587<br>O,1.5080129854,-0.3969847458,-0.7032921277<br>C,2.4828611765,-0.437274382,0.3528356517<br>C,3.8070596146,-0.8299193123,-0.2826384613<br>H,2.570445681,0.5405389743,0.839524931<br>H,2.1881465648,-1.1779534303,1.107171412<br>H,4.0798745663,-0.0898652252,-1.0476535332<br>H,3.7041153619,-1.8089153632,-0.7706838137	**27a**<br>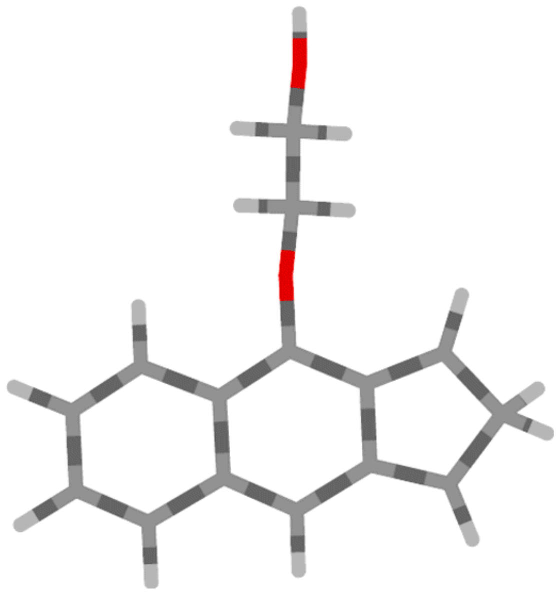<br>Freq: 27.5925<br>Sum of electronic and zero-point energies=<br>-730.199660<br>Sum of electronic and thermal energies=<br>-730.184898<br>Sum of electronic and thermal enthalpies=<br>-730.183954<br>Sum of electronic and thermal Free energies=<br>-730.242526
